# Evolving genomic landscape of pediatric pneumococcus in two Canadian urban centers following conjugate vaccination

**DOI:** 10.3389/fmicb.2025.1642658

**Published:** 2025-08-18

**Authors:** Sarah Teatero, Allison McGeer, Agron Plevneshi, Leah J. Ricketson, James D. Kellner, Nahuel Fittipaldi

**Affiliations:** ^1^Public Health Ontario, Toronto, ON, Canada; ^2^Department of Microbiology, Sinai Health System, Toronto, ON, Canada; ^3^Department of Laboratory Medicine and Pathobiology, Faculty of Medicine, University of Toronto, Toronto, ON, Canada; ^4^Department of Pediatrics, Cumming School of Medicine, University of Calgary, Calgary, AB, Canada; ^5^Alberta Children's Hospital Research Institute, Calgary, AB, Canada; ^6^Department of Community Health Sciences, University of Calgary, Calgary, AB, Canada; ^7^Department of Microbiology, Immunology, and Infectious Diseases, University of Calgary, Calgary, AB, Canada; ^8^Alberta Health Services, Calgary, AB, Canada; ^9^GREMIP and CRIPA, Faculty of Veterinary Medicine, University of Montreal, Saint-Hyacinthe, QC, Canada

**Keywords:** *Streptococcus pneumoniae*, pneumococcal conjugate vaccine (PCV), serotype replacement, antimicrobial resistance, whole-genome sequencing, genomic epidemiology, Canada (Calgary, Alberta, Toronto, Ontario), vaccine escape

## Abstract

**Background and aim:**

Pneumococcal conjugate vaccines (PCVs) have significantly reduced pediatric invasive pneumococcal disease (IPD). However, vaccine escape variants, the emergence of non-vaccine serotypes (NVTs), and antimicrobial resistance (AMR) remain ongoing concerns. We aimed to characterize long-term trends in serotype distribution, lineage composition, and AMR patterns among pediatric IPD cases following PCV introduction in two major Canadian urban centers: Calgary, Alberta, and Toronto, Ontario.

**Methods:**

We combined whole-genome sequencing with epidemiologic data from 818 pediatric IPD isolates collected through population-based, active surveillance in Calgary (1998–2016) and Toronto (2009–2016). Phylogenetic analyses, serotype characterization, and AMR profiling were performed to assess temporal trends across three vaccine eras.

**Results:**

PCV implementation reduced vaccine type serotypes but was followed by expansion of NVTs, including serotypes 22F, 33F, and 15B, with regional differences in prevalence. Serotypes 19A and 3 persisted despite PCV13 inclusion. Several pre-vaccine-associated lineages re-emerged under NVT capsules, indicating capsular switching. Macrolide resistance increased in Calgary (7.4–29.3%), distributed across multiple lineages; penicillin resistance remained infrequent.

**Conclusion:**

Our findings highlight sustained pneumococcal adaptation through serotype replacement, vaccine escape, and AMR dissemination. Ongoing genomic surveillance is essential to monitor these trends and inform vaccination policies.

## Introduction

*Streptococcus pneumoniae*, commonly known as the pneumococcus, remains a leading cause of serious infections worldwide, particularly among young children, despite widespread vaccination efforts. The introduction of pneumococcal conjugate vaccines (PCVs) has dramatically reduced the burden of pediatric invasive pneumococcal disease (IPD), with significant declines in meningitis, bacteremia, and pneumonia reported across many settings, including Canada (Centers for Disease Control and Prevention, 2021; Duke and Avci, 2023; National Advisory Committee on Immunization, 2002; Scheifele et al., 2000). However, the ability of the pneumococcus to adapt to vaccine-induced selective pressures continues to challenge disease prevention efforts.

Understanding how pneumococcal populations evolve under vaccine pressure remains essential to sustaining and improving the impact of PCV programs. The continuous effectiveness of these public health successes depends on the adaptability of PCVs to a rapidly evolving pneumococcal landscape. The pneumococcus is a highly diverse organism, with more than 100 distinct capsular types, or serotypes, that differ in disease severity, antimicrobial susceptibility, and global distribution. The pneumococcal polysaccharide capsule is a key virulence factor that protects the bacterium against phagocytosis (Bentley et al., 2006; Ganaie et al., 2020; Hackel et al., 2013) and is also the target of commercially available PCVs (Mostowy and Holt, 2018; Sari et al., 2024). Because of this vast diversity of serotypes, initial and subsequent PCV formulations were designed to provide protection against the pneumococcal serotypes most commonly responsible for pediatric IPD at different time periods (Pilishvili et al., 2010).

The National Advisory Committee on Immunization (NACI) provides guidelines for vaccine use in Canada, but provinces implement these recommendations on their own timelines. The heptavalent vaccine PCV7 (Pneu-C-7, marketed as Prevnar®, Wyeth-Ayerst, Canada), protecting against serotypes 4, 6B, 9V, 14, 18C, 19F, and 23F, was authorized in 2002 for routine immunization of children and was introduced across all Canadian provinces and territories between 2002 and 2006 (Rennels et al., 1998; National Advisory Committee on Immunization, 2002). In parts of Canada, PCV7 was replaced by the 10-valent vaccine PCV10 (Pneu-C-10, Synflorix®, GlaxoSmithKline Biologicals SA), which became available in 2009 and protected against all PCV7 serotypes plus serotypes 1, 5, and 7F (Desai et al., 2010). In 2010–2011, the 13-valent vaccine PCV13 (Pneu-C-13, Prevnar 13®, Wyeth/Pfizer Vaccines), comprising all PCV7 serotypes plus serotypes 1, 3, 5, 6A, 7F, and 19A, was incorporated into the routine immunization schedule in Alberta and Ontario, replacing PCV7 and PCV10 (Desai et al., 2010). In 2024, NACI recommended the use of the 15-valent vaccine (Pneu-C-15, Vaxneuvance®, Merck) adding serotypes 22F and 33F to PCV13, or the 20-valent vaccine (Pneu-C-20, Prevnar 20®, Pfizer), adding serotypes 8, 10A, 11A, 12F, 15B, 22F, and 33F to PCV13 (National Advisory Committee on Immunization, 2024).

As mentioned, the advent of pediatric pneumococcal vaccination programs in Canada led to a reduction of the incidence of IPD caused by most pneumococcal serotypes included in the different vaccines (henceforth the vaccine types, or VTs) (Croucher et al., 2013; Kellner et al., 2009; Leal et al., 2012; Ricketson et al., 2018; Yildirim et al., 2017). However, the use of PCVs also led to the persistence and often to the expansion of non-vaccine serotypes (NVT), resulting in an increase of invasive disease caused by these serotypes (Balsells et al., 2017; Weinberger et al., 2011). The pneumococcus is naturally competent, i.e., capable of efficiently acquiring and incorporating exogenous DNA. This ability endows the pathogen with the capacity for rapid adaptation to selective pressures such as vaccination (Croucher et al., 2011). For instance, genomic lineages associated with VT serotypes prior to vaccine introduction can persist by acquiring NVT capsules through a mechanism known as capsular switching (Wyres et al., 2013). The remarkable genomic plasticity of the organism, defined as its ability to evolve rapidly through recombination, capsular switching, and horizontal gene transfer, has made whole genome sequencing (WGS) the preferred molecular tool for genotyping this species. In 2019, the Global Pneumococcal Sequencing Consortium introduced a genome-based genotyping framework, known as Global Pneumococcal Sequence Clusters (GPSCs), which enables high-resolution surveillance of pneumococcal population dynamics and global tracking of its evolutionary patterns (Gladstone et al., 2019).

This genetic plasticity also contributes to the emergence and spread of antimicrobial resistance (AMR) in pneumococcal populations. Throughout the 20th century, resistance to commonly used *β*-lactams steadily increased, and by the year 2000, up to 40% of pneumococcal infections were caused by bacteria resistant to at least one antibiotic.^1^ Following the introduction of PCVs for pediatric immunization, some regions, including parts of Canada, reported a decrease in non-susceptibility to certain antibiotics (Tyrrell et al., 2009). This decline likely reflects the reduction of specific resistant clones targeted by the vaccine. However, in some settings, resistance persisted or even increased, largely driven by the expansion of resistant NVT clones, highlighting the ongoing challenge of controlling pneumococcal disease in the face of continued selective pressure from antibiotics and the pathogen’s ability to adapt (Hanage et al., 2007; Kim et al., 2016).

To better understand pneumococcal adaptation to vaccination, we combined whole-genome sequencing of isolates causing pediatric IPD with clinical data from population-based, active surveillance programs in Calgary and Toronto. This approach enabled us to investigate the evolutionary dynamics of pneumococcal populations, by examining how phylogenetic structure, serotype distribution and AMR shifted over time in response to vaccine implementation, with particular focus on genotype–serotype associations, serotype replacement, the emergence of vaccine escape variants, and temporal trends in resistance across three distinct vaccine eras.

## Materials and methods

### Study population, epidemiological data collection, and other definitions

A pediatric IPD case was defined as an acute illness occurring in a person 17 years old or younger from whom pneumococcus had been isolated from a normally sterile body site (e.g., blood, cerebrospinal fluid, synovial fluid, pericardial fluid, pleural fluid, lung tissue, or peritoneal fluid). Cases were identified through two population-based, active, laboratory-based surveillance programs: the Calgary Area *Streptococcus pneumoniae* Epidemiology Research (CASPER) and the Toronto Invasive Bacterial Diseases Network (TIBDN). Both programs use standardized methods and forms to collect clinical data, including demographic information, disease manifestations, and underlying medical conditions, for all patients with IPD. CASPER catchment area includes the city of Calgary, Alberta, and surrounding communities (henceforth, for simplicity, referred to as “Calgary”), while TIBDN covers the city of Toronto and Peel region, Ontario (henceforth referred to as “Toronto”).

All but 10 pediatric IPD cases recorded by CASPER between 1998 and 2016 were included (*N* = 338); exclusions were due to technical issues (see Supplementary Methods for details). The population of the catchment area of CASPER increased from 0.89 to 1.4 million people during the study period (1998–2016). We also included all 480 pediatric cases recorded by TIBDN between 2009 and 2016. During this period, the population within the TIBDN active surveillance area increased from 3.9 to 4.3 million. Data collection and usage were approved by the Research Ethics Boards of participating CASPER and TIBDN institutions.

**Figure 1:**
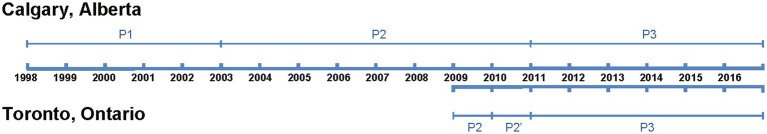
Timeline of study period and pneumococcal conjugate vaccine (PCV) implementation in Alberta and Ontario, Canada. The study period for each province is indicated by a thick blue line. Thin blue lines represent the periods during which different PCV formulations were in use. In Calgary, Alberta, the study spanned from 1998 to 2016, with three defined periods: P1 (pre-PCV), P2 (PCV7 period), and P3 (PCV13 period). In Toronto, Ontario, the study period was from 2009 to 2016, encompassing three periods: P2 (PCV7 period), P2’ (PCV10 period; 1 year only), and P3 (PCV13 period). Calgary (Alberta) provided the primary dataset used for longitudinal analysis, as it includes full coverage across P1 to P3. Toronto (Ontario) was used for supporting comparisons, particularly in the PCV13 period.

We divided the study into three distinct periods based on the timing of PCV introduction: P1, or the pre-PCV period, covers the years before a routine, publicly funded pediatric PCV was implemented, and includes the year of the first PCV introduction. For Alberta, this is before January 1, 2003, and for Ontario, it is before January 1, 2006; P2, or the PCV7 period, represents the time when PCV7 was in use, starting from the year after introduction of routine publicly funded PCV7 until the first year of introduction of PCV10 (Ontario only) or PCV13 introduction (Alberta). Thus, in Alberta, P2 spans from January 1, 2003, to December 31, 2010. In Ontario, it spans from January 1, 2006, to December 31, 2009. P2’, or the PCV10 period is a specific period for Ontario, covering the time PCV10 was in use. It runs from the year after PCV10 introduction until the year after PCV13 introduction, i.e., from January 1, 2010, to December 31, 2010. P3 or the PCV13 period, includes the years when PCV13 was in use, starting from the first year after its introduction until the end of the study period. In both Alberta and Ontario, P3 spans from January 1, 2011, to December 31, 2016. In summary, the Calgary dataset provides complete coverage of all three pediatric vaccine eras (P1–P3) and is used for all longitudinal analyses. The Toronto dataset, which begins in the late PCV7 era, is used in a supporting role to evaluate whether PCV13-era trends observed in Calgary were consistent in a second major urban center. Figure 1 provides a graphical representation of the vaccine implementation timeline and study periods.

### Bacterial isolates

We included a single pneumococcal isolate for each of the pediatric IPD cases defined above, for a total of 818 pediatric isolates (Supplementary Tables 1, 2). Irrespective of source, pneumococcal isolates were identified as per standard methods (Facklam and Breiman, 1991) and serotyped using the Quellung reaction (Austrian, 1976) with serotype specific antisera from the Statens Serum Institute (Copenhagen, Denmark). We grouped serotypes based on inclusion in PCV13, the highest-valent vaccine implemented in pediatric programs during the study period into the following two categories: VT included serotypes 1, 3, 4, 5, 6A, 6B, 7F, 9V, 14, 18C, 19A, 19F, and 23F; NVT included all serotypes not included in PCV13 as well as nontypeable (NT) isolates. For comparison purposes, we additionally included 261 adult IPD isolates (that is, bacteria isolated from normally sterile body sites of persons aged 18 years or older) recovered by CASPER’s active surveillance from 2000 to 2016 (Supplementary Tables 1, 2). These CASPER adult IPD isolates were selected to be representative of the serotype distribution observed among all adult IPD Calgary during the period.

### DNA extraction and genome sequencing

Bacterial isolates from the TIBDN and CASPER collections were cultured, and DNA extraction performed, as previously described (Duvvuri et al., 2016). Genomic libraries were prepared using Nextera XT Library Prep Kits (Illumina, CA, USA), and sequenced as paired end reads (125 bp + 125 bp) on an Illumina HiSeq4000 instrument at the sequencing facilities of Génome Québec (Montreal, QC, Canada). More detailed methods are provided in the Supplementary Methods. Sequence reads have been deposited in NCBI’s Sequence Read Archive under the accession numbers provided in Supplementary Table 2.

### Genome assemblies, annotations, and phylogenetic analysis

*de novo* assemblies were generated with the A5-miseq pipeline (Coil et al., 2015) using genome data for 1,287 isolates, i.e., the 1,079 newly sequenced genomes from CASPER and TIBDN pediatric and adult isolates, as well as previously published (Deng et al., 2016) genome data for 208 adult IPD isolates recovered by Public Health Ontario (PHO, passive surveillance system) in the province of Ontario during 2009–2013 (Supplementary Tables 1, 2). Resulting contigs were annotated with Prokka (v. 1.12) (Seemann, 2014). Phylogenetic analysis was performed using PopPUNK (v.2.7.0) (Lees et al., 2019). GPSCs were assigned using PopPUNK and the GPSC reference database v9.^2^ Alternate ortholog analysis, and core- and accessory genome determinations were carried out with Roary (v.3.12.0) (Page et al., 2015). Maximum likelihood phylogenies were inferred from the core genome alignments generated by Roary using FastTreeMP (v.2.1.8) (Price et al., 2010), after accounting for recombination, which was examined with Gubbins (Croucher et al., 2015). Further details can be found in the Supplementary Methods.

### Antimicrobial susceptibility testing

Antimicrobial susceptibility testing was performed by broth microdilution or E-test following Clinical and Laboratory Standards Institute (CLSI) guidelines and interpretive criteria (breakpoints) to define susceptibility categories (CLSI, 2019). Amoxicillin testing was performed only on samples with a minimum inhibitory concentration (MIC) for penicillin > 0.12 μg/mL.

### Statistical analyses

Differences across vaccine periods for categorical variables (e.g., serotype distribution, AMR profiles) were evaluated using the chi-square test or Fisher’s exact test, as appropriate, based on expected cell counts. Analyses were stratified by vaccine era, age group, region, and genetic lineage where applicable. Serotype replacement, resistance emergence, and capsule switching events were assessed using these comparisons to highlight significant shifts in population structure over time. All statistical analyses were performed using R version 4.2.3 (R Core Team, 2023). Analyses related to genomic clustering, phylogenetic reconstruction, and capsule switching are described within their respective bioinformatics sections in the Supplementary Methods.

## Results

### Evolution of the population structure of pneumococcal pediatric IPD isolates in Calgary

**Figure 2:**
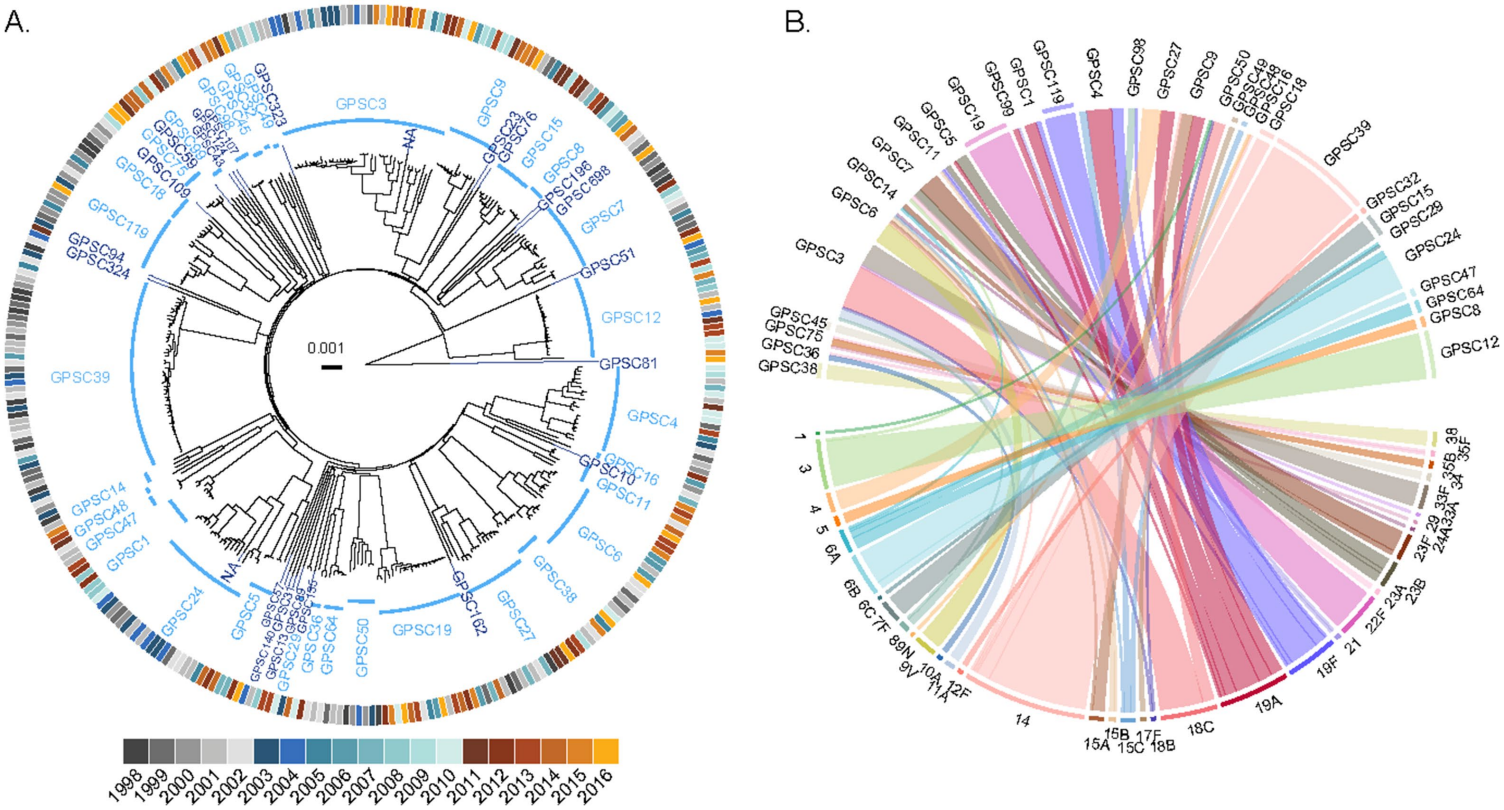
Complex population structure of pediatric IPD isolates from the Calgary area. **(A)** Phylogenetic relationships between 338 pediatric pneumococcus isolates causing pediatric IPD in Calgary between 1998 and 2016. The phylogenetic tree was constructed using PopPUNK and rooted on the outlier strain CSPN0301 (a non-typable GPSC81 isolate). GPSCs are shown in blue, with multi-isolate clusters in light blue and singletons in dark blue. The outer ring indicates the year of isolation, colored by vaccine period: P1 (pre-PCV period, shades of gray), P2 (PCV7 period, shades of blue), and P3 (PCV13 period, shades of yellow). **(B)** Relationship between GPSCs and serotypes among pediatric IPD isolates. The upper half of the plot displays GPSCs with two or more genomes (singletons are not represented), and the lower half shows their associated serotypes. Ribbons connect each GPSC to its corresponding serotype(s) and are colored according to serotype. The outer ring segments indicates whether each GPSC is associated with a single serotype (colored according to serotype). No outer ring segment is displayed for GPSCs associated with multiple serotypes.

We sequenced the genomes of a collection of 338 pneumococcal isolates recovered from pediatric patients in the Calgary area, Alberta, from 1998 to 2016, and representing virtually all pediatric IPD cases during this period. We next used PopPUNK (Lees et al., 2019) to define the genotypes of the isolates and investigate the phylogenetic relationships between them. We identified a total of 58 GPSCs among this pediatric population (Figure 2A). Of them, 24 were represented by a single isolate. Among the GPSCs with at least two isolates, 14 consisted entirely of isolates of a single serotype, representing 44% (138/314) of the genomes (Supplementary Table 3). However, there were complex associations of serotypes with some GPSCs: 12 GPSCs, representing 21% or 67/314 genomes, contained 2 serotypes, while 8 other GPSCs, representing 35% or 109/314 genomes, contained 3 or more serotypes (Figure 2B). Some serotypes were broadly represented throughout the population, with serotypes 19A and 19F found in 6 and 5 different GPSCs, respectively (Figure 2B and Supplementary Table 3).

To complement PopPUNK clustering and investigate fine-scale phylogenetic relationships, we used Roary (Page et al., 2015) to define the core genome of the 338 pediatric IPD strains from the Calgary area. The resulting core genome alignment contained 44,793 variant positions, with a median pairwise single-nucleotide polymorphism (SNP) distance between all 338 strains of 5,531 (IQR 5,196-5,872). The core genome phylogeny was largely consistent with the PopPUNK-based clusters (Supplementary Figure 1), although some differences in the arrangement of lineages were observed, reflecting methodological differences. Upon further inspection of the core genome, we found that the pairwise SNP distance between all strains increased over time. Among the 136 strains isolated pre-vaccine introduction (P1), the median SNP distance was 5,340 (IQR 5,026–5,713); among the 103 strains collected during the use of PCV7 (P2), the median SNP distance was 5,471 (IQR 5,156–5,768), while among the 99 isolates collected after the introduction of PCV13 (P3), the median SNP distance was 5,600 (IQR 5,374–5,934). While these differences are small, they are consistent with a gradual diversification of the pneumococcal population over the study period.

**Figure 3:**
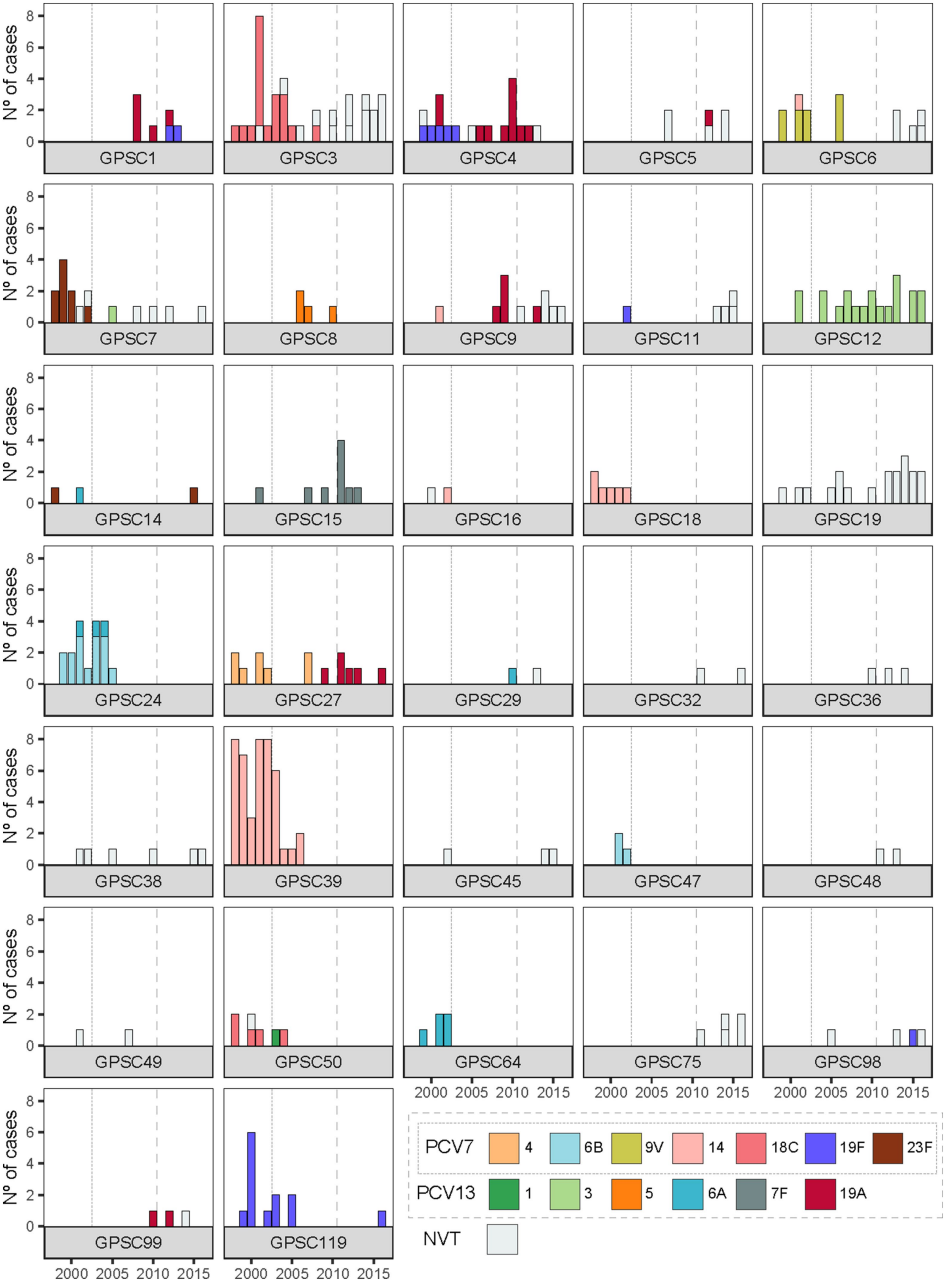
Temporal dynamics of serotype composition within GPSCs in Calgary pediatric IPD isolates. Each panel shows the serotype distribution over time within GPSCs comprising two or more genomes. Vaccine-included serotypes (PCV7 serotypes 4, 6B, 9 V, 14, 18C, 19F, and 23F; additional PCV13 serotypes 1, 3, 5, 6A, 7F, and 19A) are shown in color, while non-vaccine serotypes (NVTs) are shown in grey. Vertical dotted and dashed lines indicate the introduction of PCV7 and PCV13 in Alberta, respectively.

This trend prompted us to examine how the composition of GPSCs changed over time, revealing notable shifts in their distribution (Figure 3). Some GPSCs, such as GPSC8 and GPSC18, which contained only VT isolates, disappeared from the population after the introduction of PCV7 and/or PCV13 (Figure 3). Some other GPSCs, which contained VT isolates pre-vaccine, persisted after the introduction of PCV7 or PCV13, likely due to capsular switching to NVTs. For example, GPSC6 which included isolates of serotype 9V and 14 in P1 and P2, persisted and was represented in P3 by isolates of NVTs 15A, 15B, 15C, and 24A. Other GPSCs, which consisted exclusively of NVT isolates before vaccine introduction, experienced an expansion over time. For example, GPSC19, which consists only of serotype 22F isolates, showed increased prevalence by P3. Some GPSCs showed expansion with persistence of VT isolates despite the introduction of the vaccines. Clear examples were GPSC12 isolates of serotype 3 and GPSC15 isolates of serotype 7F (Figure 3). Supplementary Figure 2 presents PopPUNK-based subtrees for selected GPSCs, providing a complementary visual perspective on the lineage-specific changes. The VT/NVT split over time for each GPSC is presented in Supplementary Table 4.

### Divergent dynamics of serotypes 19A and 19F across vaccine eras

Before vaccination, isolates of serotype 19F belonged to three distinct genetic lineages: GPSC4, GPSC11, and GPSC119 (Figure 3). With the introduction of PCV7 and PCV13, both targeting serotype 19F, substantial changes occurred: isolates of serotype 19F belonging to GPSC11 were eliminated from the population. GPSC11 re-emerged after the introduction of PCV13 but under NVT serotypes, indicating possible capsular switching or, more likely, the introduction of new strains of this lineage into Calgary from elsewhere. GPSC4 experienced a replacement of serotype 19F isolates by, mostly, isolates of serotype 19A after PCV7 introduction. Serotype 19F isolates belonging to GPSC119 disappeared after 2005 but were observed again in 2016. Furthermore, serotype 19F isolates of genetic backgrounds GPSC1 and GPSC98 were observed in Calgary for the first time after introduction of PCV13, suggesting capsular exchange events and/or novel introductions to the community.

Serotype 19A displayed different dynamics. Before vaccination, this serotype was found infrequently, and in only one genetic background (GPSC4). However, after the introduction of PCV7, which does not target serotype 19A, it emerged in five additional clusters: GPSC1, GPSC5, GPSC9, GPSC27, and GPSC99 (Figure 3). The emergence of serotype 19A across multiple GPSCs was likely due to capsular switching events, as exemplified by GPSC27, where serotype 4 isolates were completely replaced by isolates of serotype 19A. Similarly, GPSC9 saw a replacement of serotype 14 by serotype 19A and NVT isolates. These examples suggest that it is likely that the pressure exerted by PCV7 on the serotypes it targeted may have facilitated the spread of serotype 19A. Interestingly, the introduction of PCV13, which targets serotype 19A, did not eliminate this serotype from any of these 5 genetic backgrounds. Indeed, serotype 19A persisted in multiple GPSCs well after PCV13 introduction (Figure 3). Consistent with this, when we compared serotype 19A SNP diversity across the vaccine eras in Calgary pediatric isolates, we observed that the median SNP distance rose from 205 SNPs in the pre-vaccine era to 5,388 SNPs in the PCV7 era, and remained high at 5,468 SNPs in the PCV13 era.

### Post-PCV13 changes in serotype distribution in Calgary and Toronto

**Figure 4:**
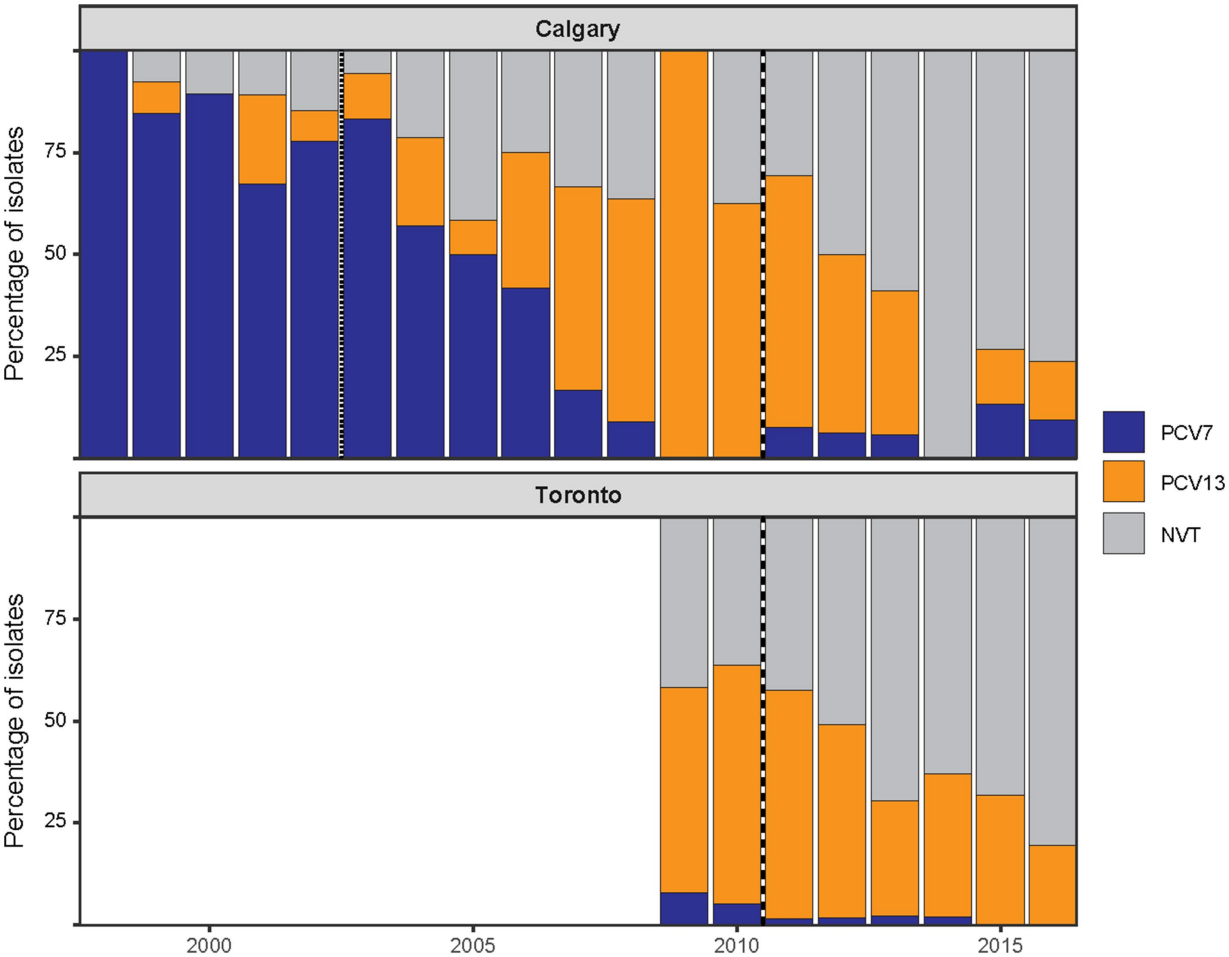
Post-PCV13 changes in serotype composition of pediatric IPD in Calgary and Toronto. The annual distribution of pediatric IPD isolates by vaccine-type (VT) status in Calgary (CASPER, 1998–2016) and Toronto (TIBDN, 2009–2016). Isolates are grouped according to serotype inclusion in PCV7 (serotypes 4, 6B, 9V, 14, 18C, 19F, and 23F; shown in blue), inclusion in the additional six serotypes of PCV13 (serotypes 1, 3, 5, 6A, 7F, and 19A; shown in orange), or as non-vaccine serotypes (NVT; shown in grey). Vertical dotted or dashed lines mark the introduction of PCV7 (dotted) and PCV13 (dashed) in Alberta and PCV13 in Ontario (dashed). No data were included in this study for Toronto prior to 2009. Despite regional differences in vaccine implementation, similar post-PCV13 trends were observed in both cities, characterized by a decline in VTs and a corresponding increase in NVTs. Overall VT/NVT proportions during overlapping years were not significantly different between regions.

To determine whether the post-PCV13 patterns observed in Calgary were unique or part of a broader trend, we analyzed an additional collection of 480 pneumococcal pediatric IPD isolates from Toronto. The overall serotype distribution was slightly different: Calgary isolates encompassed 26 serotypes plus one NT isolate, while the Toronto collection comprised 33 serotypes plus two NT isolates (Supplementary Table 5). When serotypes were grouped as VT and NVT, there were no statistically significant differences in the overall proportion of VT and NVT between the two regions during the overlapping study time period (2009–2016, *χ*^2^ = 1.29, *p* = 0.26). Although some year-to-year variation is apparent, particularly in the Calgary data, the aggregate distributions were comparable (Figure 4).

Similar to Calgary, in Toronto most PCV7 and PCV13 VTs declined after P2 or P3, respectively, but serotypes 3 and 19A did not show a significant decrease in prevalence following the introduction of PCV13 (Table 1 and Supplementary Figure 3). Moreover, when we compared the proportion of serotypes with more than five isolates between the two geographical areas, we found that serotype 19A was significantly more prevalent in Toronto than in Calgary (Supplementary Table 5). To further explore the persistence of serotype 19A despite vaccination, we examined the vaccination status of patients with these infections. We identified IPD cases caused by serotype 19A in both Calgary and Toronto among individuals who had received at least one dose of PCV13. In Calgary, five such cases were identified, attributed to GPSC4, GPSC9, GPSC27 (*n* = 2), and GPSC99, and two of them were children who had completed the full four-dose series of PCV-13 (GPSC27 and GPSC99). In Toronto, there were 22 cases belonging to GPSC1 (*n* = 5), GPSC4 (*n* = 4), GPSC27 (*n* = 11), GPSC99 (*n* = 1) and GPSC135 (*n* = 1), of which four were found in children with the complete PCV13 schedule (three GPSC27 and one GPSC1). These findings suggest that serotype 19A has persisted across different genetic backgrounds through capsule switching despite vaccination efforts, highlighting the need for ongoing surveillance and potentially updated vaccine formulations.

**Table 1:** Persistence of vaccine-type serotypes in pediatric IPD cases in Calgary and Toronto.

Urban center	PCV7 serotypes	P1 (1998–2002)—N° (%)^a^	P2 and P3 (2003–2016)—N° (%)	*p-*value^b^
Calgary	4	7 (5.2)	2 (1.0)	0.033
	6B	12 (8.8)	8 (4.0)	
	9V	6 (4.4)	3 (1.5)	
	14	43 (31.6)	10 (5.0)	<0.001
	18C	17 (12.5)	9 (4.5)	0.012
	19F	14 (10.3)	9 (4.5)	
	23F	10 (7.4)	3 (1.5)	0.0082
	NVT	27 (19.9)	158 (78.2)	<0.001
	Total	136 (100)	202 (100)	

Urban center	PCV13 serotypes	P1 and P2 (1998–2010)—N° (%)	P3 (2011–2016)—N° (%)	*p-*value^b^
Calgary	1^c^	2 (0.8)	0 (0)	
	3^c^	13 (5.4)	9 (9.1)	
	4	9 (3.8)	0 (0)	
	5^c^	4 (1.7)	0 (0)	
	6A^c^	11 (4.6)	0 (0)	0.038
	6B	20 (8.3)	0 (0)	<0.01
	7F^c^	3 (1.3)	6 (6.1)	0.021
	9V	9 (3.8)	0 (0)	
	14	53 (22.2)	0 (0)	<0.001
	18C	26 (10.9)	0 (0)	<0.001
	19A^c^	21 (8.8)	11 (11.1)	
	19F	19 (7.9)	4 (4.0)	
	23F	10 (4.2)	3 (3.0)	
	NVT	39 (16.3)	66 (66.7)	<0.001
	Total	239 (100)	99 (100)	

Urban center	PCV13 serotypes	P2 and P2’ (2009–2010)—N° (%)	P3 (2011–2016)—N° (%)	*p-*value^b^
Toronto	3^d^	12 (6.7)	25 (8.3)	
	4	1 (0.6)	0 (0)	
	5	0 (0)	1 (0.3)	
	6B	1 (0.6)	1 (0.3)	
	7F	28 (15.6)	14 (4.7)	<0.001
	9V	0 (0)	2 (0.7)	
	18C	2 (1.1)	0 (0)	
	19A^d^	57 (31.7)	76 (25.3)	
	19F	5 (2.8)	1 (0.3)	0.03
	23F	3 (1.7)	0 (0)	
	NVT	71 (39.4)	180 (60.0)	<0.001
	Total	180 (100)	300 (100)	

^a^Percent of total per time period. ^b^χ2 test for statistically significant difference between time periods calculated for groups of >5 per cell, Fisher’s exact test if any cell <5. Only statistically significant values are shown. ^c^Serotypes found in PCV13 but not in PCV7. ^d^Serotypes found in PCV13 but not in PCV7 or PCV10.

### Characteristics of pediatric IPD in two Canadian urban centers

In Calgary, a total of 338 pediatric IPD cases were recorded between 1998 and 2016, distributed as follows: 146 cases (43%) in children under 2 years old, 105 cases (31%) in children aged 2–4 years, and 87 cases (26%) in children aged 5–17 years (Table 2). In Toronto, 480 pediatric IPD cases were documented between 2009 and 2016, with 134 cases (28%) in children under 2 years old, 192 cases (40%) in children aged 2 to 4 years, and 154 cases (32%) in children aged 5–17 years (Table 2). In Calgary, children under 2 years of age accounted for the highest proportion of pediatric IPD cases, consistent with their increased susceptibility to pneumococcal infection. In Toronto, cases were more evenly distributed across age groups, with the highest proportion among children aged 2–4 years. This likely reflects differences in the time frames analyzed: the Toronto data begin in 2009, after several years of PCV7 use, and when vaccine-related declines in IPD among infants were already established.

**Table 2:** Distribution of IPD cases by age group, vaccine era, and predominant GPSCs.

Age group, years	Total *N*° (%)^a^	P1^b^ N° (%)^a^	P1—Top 3 GPSCs (N°)	P2^c^ N° (%)^a^	P2—Top 3 GPSCs (N°)	P2’^d^ N° (%)	P2’—Top 3 GPSCs (N°)	P3^e^ N° (%)^a^	P3—Top 3 GPSCs (N°)
Calgary									
< 2	146 (43.2)	73 (53.7)	39 (*n* = 23); 7, 24 (*n* = 6)	42 (40.8)	12 (*n* = 5); 4, 39 (*n* = 4)	–	–	31 (31.3)	3 (*n* = 8); 6, 19 (*n* = 3)
2–4	105 (31.1)	39 (28.7)	39 (*n* = 7); 3 (*n* = 6); 7, 18, 27 (*n* = 3)	34 (33.0)	3 (*n* = 6); 24 (*n* = 5); 4, 12, 39 (*n* = 3)	–	–	32 (32.3)	12 (*n* = 4); 5, 11, 15, 19 (*n* = 3)
5–17	87 (25.7)	24 (17.6)	39 (*n* = 4); 50 (*n* = 3); 3, 6, 7, 24, 27 (*n* = 2)	27 (26.2)	3 (*n* = 4); 7, 39 (*n* = 3)	–	–	36 (36.4)	19 (*n* = 5); 12 (*n* = 4); 9, 27 (*n* = 3)
18–64	203 (77.8)	19 (50.0)	27 (*n* = 7); 39 (*n* = 6); 15, 98 (*n* = 2)	120 (85.1)	8 (*n* = 50); 98 (*n* = 15); 12 (*n* = 13)	–	–	64 (78.0)	27 (*n* = 23); 12 (*n* = 15); 98 (*n* = 8)
65–84	48 (18.4)	15 (39.5)	39 (*n* = 7); 12 (*n* = 3); 27 (*n* = 2)	17 (12.1)	12 (*n* = 5); 98 (*n* = 4); 15 (*n* = 3)	–	–	16 (19.5)	12 (*n* = 7); 98 (*n* = 4); 9 (*n* = 3)
≥ 85	10 (3.8)	4 (10.5)	12, 39 (*n* = 2)	4 (2.8)	39 (*n* = 3); 12 (*n* = 1)	–	–	2 (2.4)	1, 98 (*n* = 1)
Pediatric total	338 (56.4)	136 (78.2)	39 (*n* = 34); 3 (*n* = 12); 7 (*n* = 11)	103 (42.2)	3 (*n* = 13); 39 (*n* = 10); 4, 12, 24 (*n* = 9)	–	–	99 (54.7)	3, 19 (*n* = 11); 12 (*n* = 9)
Adult total	261 (43.6)	38 (21.8)	39 (*n* = 15); 27 (*n* = 9); 12 (*n* = 5)	141 (57.8)	8 (*n* = 52); 12, 98 (*n* = 19)	–	–	82 (45.3)	27 (*n* = 23); 12 (*n* = 22); 98 (*n* = 13)
Overall	599 (100)	174 (29.0)		244 (40.7)		–	–	181 (30.2)	
Sex, male	352 (58.8)	91 (52.3)		146 (59.8)		–	–	115 (63.5)	
Source of isolation						–	–		
Blood	519 (86.6)	155 (89.1)		211 (86.5)		–	–	153 (84.5)	
CSF	43 (7.2)	14 (8.0)		16 (6.6)		–	–	13 (7.2)	
Other^f^	37 (6.2)	5 (2.9)		17 (7.0)		–	–	15 (8.3)	
Toronto									
< 2	134 (27.9)	–	–	35 (34.0)	15 (*n* = 5); 3, 10, 12, 19, 27 (*n* = 3)	28 (36.4)	1 (*n* = 5); 27 (*n* = 4); 15, 59 (*n* = 3)	71 (23.7)	11 (*n* = 11); 27 (*n* = 8); 3, 7, 19 (*n* = 5)
2–4	192 (40.0)	–	–	37 (35.9)	1 (*n* = 6); 12, 19 (*n* = 4)	26 (33.8)	15, 27 (*n* = 5); 4 (*n* = 4)	129 (43)	27 (*n* = 27); 11 (*n* = 14); 4 (*n* = 12)
5–17	154 (32.1)	–	–	31 (30.1)	15, 19, 27 (*n* = 5)	23 (29.9)	15 (*n* = 9); 11 (*n* = 3); 19 (*n* = 2)	100 (33.3)	27 (*n* = 15); 11 (*n* = 12); 19 (*n* = 11)
Pediatric total	480 (100)	–	–	103 (21.5)	15, 19 (*n* = 12); 27 (*n* = 11)	77 (16.0)	15 (*n* = 17); 27 (*n* = 9); 1,4 (*n* = 7)	300 (62.5)	27 (*n* = 50); 11 (*n* = 37); 19 (*n* = 27)
Sex, male	457 (95.2)	–		60 (58.3)		39 (50.6)		169 (56.3)	
Source of isolation									
Blood	457 (95.2)			98 (95.1)		76 (98.7)		283 (94.3)	
CSF	6 (1.3)			1 (1.0)		0 (0)		5 (1.7)	
Other^g^	17 (3.5)			4 (3.9)		1 (1.3)		12 (4.0)	
Province of Ontario									
50–64	87 (41.8)	–	–	9 (23.7)	3 (*n* = 3); 4 (*n* = 2); 9, 13, 32, 98 (*n* = 1)	15 (31.9)	15 (*n* = 3); 32 (*n* = 2); 3, 4, 5, 6, 9, 11, 19, 27, 119, 156 (*n* = 1)	63 (51.2)	15 (*n* = 8); 27 (*n* = 7); 16 (*n* = 6)
65–84	98 (47.1)	–	–	24 (63.2)	3, 19 (*n* = 3); 6, 7, 12, 32 (*n* = 2)	21 (44.7)	4 (*n* = 4); 15 (*n* = 3); 13, 16, 36 (*n* = 2)	53 (43.1)	19 (*n* = 9); 15 (*n* = 7); 3 (*n* = 6)
≥ 85	23 (11.1)	–	–	5 (13.2)	3, 9, 11, 15, 19 (*n* = 1)	11 (23.4)	3, 7, 12, 19 (*n* = 2)	7 (5.7)	19 (*n* = 2); 3, 12, 15, 23, 75 (*n* = 1)
Adult total	208 (100)	–	–	38 (18.3)	3 (*n* = 7); 19 (*n* = 4); 32 (*n* = 3)	47 (22.6)	15 (*n* = 7); 4 (*n* = 5); 3, 19 (*n* = 4)	123 (59.1)	15, 19 (*n* = 16); 3, 12, 27 (*n* = 9)

^a^In Calgary, percentages refer to pediatric or adult totals, not overall totals. Percentages have been rounded and may not sum to 100. ^b^P1, or the pre-vaccine era, is defined as the time before January 1st of the year after the universal introduction of the first PCV vaccine. In Alberta this is before January 1, 2003. In Ontario this is before January 1, 2006. ^c^P2, or PCV7-era, spans from January 1 of the year after the universal introduction of PCV7, until January 1 of the year after the introduction of either PCV13 (Alberta) or PCV10 (Ontario). In Alberta, this is January 1, 2003—December 31, 2010. In Ontario, this is January 1, 2006—December 31, 2009. Note the beginning of sample availability is January 1, 2009. ^d^P2’, or thePCV10-era, is only applicable in Ontario. It spans from January 1 of the year after the universal introduction of PCV10, until January 1 of the year after the universal introduction of PCV13. This is January 1, 2010—December 31, 2010. ^e^P3, or the PCV13-era, spans from January 1 of the year after the universal introduction of PCV13, until the end of the collection period. In Alberta and Ontario, this is January 1, 2011—December 31, 2016. ^f^Other invasive source of infection includes eye, joint fluid, peritoneal fluid, pleural fluid, and other sterile sites.

In Calgary, IPD in the pre-vaccine period (P1) was characterized by a predominance of PCV7-targeted serotypes, particularly serotype 14, followed by 18C, 19F, 6B, and 23F (Table 1). In children under 2 years of age, serotype 14 cases were mainly associated with GPSC39, while serotype 23F was linked to GPSC7, serotype 6B to GPSC24, and serotype 19F to GPSC119 (Supplementary Figure 4). During the PCV7 period, there was a marked decline in cases among children under 2 years old, along with a reduction of PCV7 serotypes (Table 2). Among children aged 2–4 years, a similar decline in PCV7 serotypes like serotype 14 was observed, while GPSC12 (serotype 3) emerged. In this age group, isolates of serotype 19A belonged to GPSC1 and GPSC9, among others (Supplementary Figure 4). For children aged 5–17 years, IPD cases remained relatively constant across vaccine periods, but shifts in GPSC and serotype distribution mirrored those in younger children (Supplementary Figure 4). During the PCV13 period (P3), NVT 22F, associated with GPSC19, became more frequent in all pediatric age groups, alongside persistence of serotype 3 (GPSC12) and serotype 19A associated with many GPSC (Tables 1, 2).

In Toronto, similar trends were observed, although with some regional differences in serotype distribution. By P3, NVTs such as serotypes 22F and 15B, along with VTs 19A and 3, became predominant across pediatric age groups (Table 1). Supplementary Figure 5 shows that in children under 2 years of age, serotype 19A was primarily associated with GPSC27, serotype 3 with GPSC12, and serotype 22F with GPSC19. In children aged 2–4 years, GPSC11 (serotype 15B), GPSC27 (serotype 19A), GPSC19 (serotype 22F), and GPSC12 (serotype 3) were the dominant lineages during the PCV13 period. Among children aged 5–17 years, a similar pattern was observed, with GPSC15 (7F), GPSC12 (3), GPSC19 (22F), and GPSC27 (19A) being the most frequent GPSCs, reflecting emergence of NVTs and incomplete suppression of vaccine lineages despite vaccination (Supplementary Figure 5).

### Potential spillover effects of pediatric vaccination on adult IPD trends

Given the potential for indirect effects of pediatric PCV introduction on adult IPD, we examined whether changes in adult serotype and lineage distribution coincided with trends observed in children. It is important to interpret these trends in the context of pneumococcal vaccination programs for adults, which during the study period relied exclusively on the 23-valent pneumococcal polysaccharide vaccine (PPV23), as PCVs were not part of adult immunization schedules. In Calgary, 261 adult IPD isolates were analyzed, selected to reflect the serotype distribution among 2,226 total adult cases recorded between 2000 and 2016. During P2, the proportion of adult cases increased among individuals aged 18–64 years, a group unlikely to have received pneumococcal vaccination (Table 2). This increase paralleled a decline in VTs among children and was accompanied by a rise in serotype 19A in adults. The majority of adult 19A isolates belonged to GPSC4, GPSC9 and GPSC27, lineages also prevalent in pediatric cases during this period (Table 2 and Supplementary Figure 4). Among adults aged 65 years and older, increases were observed in serotypes 3, 8, and 19A following the introduction of PCV13. These trends reflected the post-PCV13 expansion of GPSC12 (serotype 3), GPSC98 (serotype 8), and GPSC27 (serotype 19A) also seen in pediatric cases (Table 2 and Supplementary Figure 4).

In Ontario, adult IPD genome and serotype data were available for 208 cases recorded at the provincial level between 2009 and 2016. Although these data represent a selection from the entire province and not Toronto specifically, they offer insight into broader trends. During P3, serotypes 3 and 22F became predominant among adults aged ≥50 years, consistent with their increased frequency in pediatric cases from Toronto (Table 2). The GPSC profiles of Ontario adults largely mirrored those observed in Toronto pediatric IPD, with serotype 3 linked to GPSC12 and serotype 22F to GPSC19 (Table 2). In both adult and pediatric datasets, serotype 33F was infrequently detected. These findings suggest that pediatric vaccination likely contributed to shaping serotype and lineage patterns in adult IPD, although additional factors such as regional epidemiology and adult vaccination strategies may also have influenced these dynamics.

### AMR persists at low levels

Overall, AMR among pediatric IPD isolates from Calgary remained relatively low but exhibited notable temporal trends across vaccine eras (Table 3). The proportion of isolates non-susceptible to at least one antimicrobial agent increased over time, primarily due to rising macrolide resistance. In contrast, non-susceptibility to *β*-lactam antibiotics remained infrequent. Non-susceptibility to trimethoprim-sulfamethoxazole remained stable at approximately 20% throughout the study period. No resistance to levofloxacin was detected.

**Table 3:** Antimicrobial susceptibility patterns in pediatric IPD isolates from Calgary.

Antimicrobial agent	Total non-susceptible^a^ *N* (%)	Period—*N* (%)			*p*-value^b^
		P1^c^	P2^d^	P3^e^	
Amoxicillin^f^	3 (1.3)	0 (0.0)	3 (4.8)	0 (0.0)	0.035
Penicillin^g^	12 (3.5)	2 (1.5)	7 (6.8)	3 (3.0)	
Cefotaxime^g^	4 (1.2)	0 (0.0)	1 (1.0)	3 (3.0)	
Ceftriaxone^g^	2 (0.6)	0 (0.0)	0 (0.0)	2 (2.0)	
Cefuroxime	25 (7.4)	11 (8.1)	6 (5.8)	8 (8.1)	
Erythromycin	54 (16.0)	10 (7.4)	15 (14.6)	29 (29.3)	<0.0001
Genes *mefA_msrD*		5 (50.0)	6 (40.0)	16 (55.2)	
Gene *ermB*		4 (40.0)	4 (26.7)	10 (34.5)	
Genes *mefA_msrD* + *ermB*			4 (26.7)	3 (10.3)	
No known marker		1 (10.0)	1 (6.7)		
Levofloxacin	0 (0.0)	0 (0.0)	0 (0.0)	0 (0.0)	
Trimethoprim-sulfamethoxazole	67 (19.8)	25 (18.4)	21 (20.4)	21 (21.2)	
Total	338	136	103	99	

^a^Data shown for isolates that met breakpoints above susceptibility as defined by CLSI (see section “Materials and methods”). ^b^χ^2^ (or Fisher’s test where appropriate) for statistically significant difference between eras. Only statistically significant values are shown. ^c^P1, or the pre-vaccine era is defined as the time before January 1st of the year after the universal introduction of the first PCV vaccine. In Alberta this is before January 1, 2003. ^d^P2, or the PCV7-era, spans from January 1 of the year after the universal introduction of PCV7, until January 1 of the year after the introduction of PCV13. In Alberta, this is January 1, 2003—December 31, 2010. ^e^P3, or the PCV13-era is defined from January 1 of the year after the universal introduction of PCV13, until the end of the collection period. In Alberta, this is January 1, 2011—December 31, 2016. ^f^A total of 23 isolates were not tested for amoxicillin. ^g^CLSI breakpoints were applied based on meningitis status.

**Figure 5:**
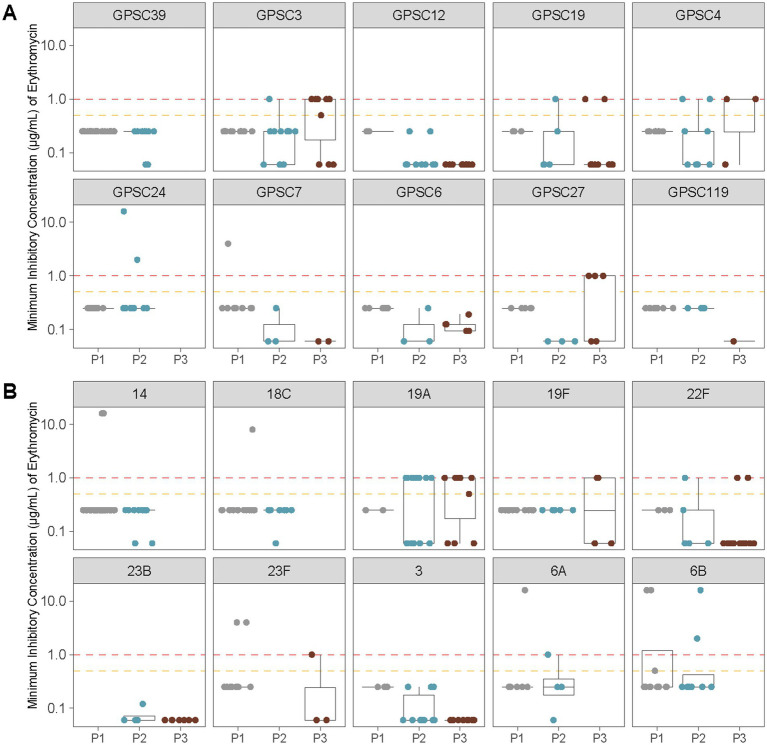
Erythromycin MIC distributions across vaccine eras for the 10 most common GPSCs and serotypes identified in pediatric IPD isolates from Calgary. **(A)** Erythromycin MICs (μg/mL) for the 10 most prevalent GPSCs among pediatric samples are shown before vaccine introduction (P1), after PCV7 introduction (P2), and after PCV13 introduction (P3). Horizontal lines indicate interpretive criteria for erythromycin susceptibility: intermediate = 0.5 μg/mL (orange) and resistant ≥1 μg/mL (red). Isolates with MIC ≤0.25 μg/mL were considered susceptible. **(B)** As in **(A)** showing erythromycin MICs (μg/mL) for the 10 most common serotypes among pediatric samples.

The most significant increase in AMR was observed for erythromycin. Non-susceptibility rose from 7.4% in P1 to 14.6% in P2, and further to 29.3% in P3 (*p* < 0.0001; Table 3). This trend was accompanied by a shift in the distribution of macrolide resistance determinants, with *mefA*/*msrD* detected in P3 in over half of resistant isolates and *ermB* in approximately one-third. To further investigate this trend, we analyzed erythromycin MIC distributions across the 10 most common GPSCs and serotypes identified in pediatric cases (Figure 5). High MICs were observed in P3 across multiple clusters, including GPSC3 (serotype 33F), GPSC4 and GPSC27 (serotype 19A). In contrast, GPSC12 (serotype 3) and GPSC19 (serotype 22F) consistently maintained low MICs (<0.5 μg/mL) across all vaccine eras. This pattern reflects the dissemination of macrolide resistance determinants across diverse lineages, rather than expansion of a single resistant clone.

In contrast to macrolides, resistance to β-lactam antibiotics remained limited throughout the study period. Non-susceptibility to penicillin increased from 1.5% in P1 to 6.8% in P2, before declining to 3.0% in P3 (Table 3). Similarly, amoxicillin non-susceptibility remained low, detected only in P2 (4.8%, *p* = 0.035). Resistance to cefuroxime remained stable across vaccine eras (<10%), while non-susceptibility to cefotaxime and ceftriaxone was rare, observed in fewer than 3% of isolates in P3.

**Figure 6:**
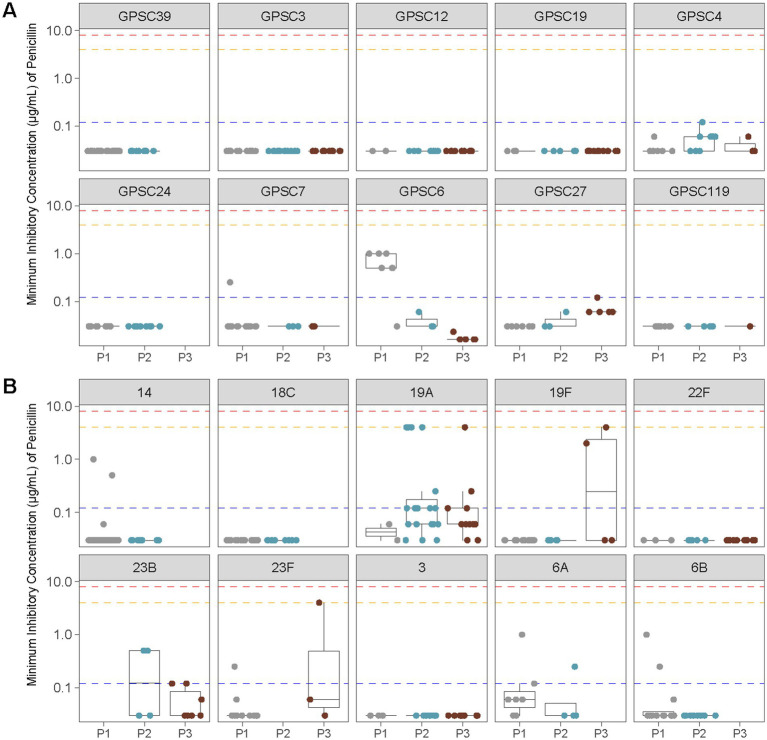
Penicillin MIC distributions across vaccine eras for the 10 most common GPSCs and serotypes identified in pediatric IPD isolates from Calgary. **(A)** Penicillin MICs (μg/mL) for the 10 most prevalent GPSCs among pediatric samples are shown before vaccine introduction (P1), after PCV7 introduction (P2), and after PCV13 introduction (P3). Horizontal lines indicate the interpretive criteria for parenteral penicillin susceptibility: resistant ≥8 μg/mL (red), intermediate = 4 μg/mL (orange) for non-meningitis isolates, and resistant ≥0.12 μg/mL (blue) for meningitis isolates. **(B)** As in **(A)**, showing penicillin MICs (μg/mL) for the 10 most common serotypes among pediatric samples.

To further examine penicillin resistance, we analyzed MIC distributions across the most common GPSCs and serotypes identified in pediatric cases. The lineage with the highest penicillin MICs, GPSC1, was primarily detected during the PCV7 era, but since this GPSC was numerically minor, it is not shown in Figure 6, which displays MIC distributions for the 10 most prevalent serotypes and GPSCs in Calgary. Among the lineages included in Figure 6, MICs remained low for most clusters and serotypes. Moderate increases were observed during P3 in GPSC4 and GPSC27 (both associated with serotype 19A). In contrast, serotype 3 (GPSC12) and serotype 22F (GPSC19) consistently maintained low MICs (<0.5 μg/mL) across all vaccine periods.

## Discussion

The introduction of PCVs has revolutionized the prevention of pediatric IPD in Canada and globally, dramatically reducing the burden of disease caused by key pneumococcal serotypes in children (Chen et al., 2019; Wilson et al., 2020). Nevertheless, the pneumococcus remains a significant public health challenge due to its remarkable capacity for genetic exchange and adaptation (Bentley et al., 2006; Centers for Disease Control and Prevention, 2021; Duke and Avci, 2023; National Advisory Committee on Immunization, 2002; Scheifele et al., 2000). In this longitudinal, population-based study spanning 18 years, we combined genome analysis of pediatric IPD isolates with epidemiologic data to characterize the dynamics of serotype and lineage composition in two major Canadian urban centers, Calgary and Toronto. Although our observational design limits the ability to disentangle the effects of vaccination from other factors such as antibiotic use or demographic shifts, our findings are consistent with prior Canadian and international reports showing that PCVs have significantly reduced specific serotypes while enabling the expansion of several NVTs and the persistence of certain VTs across multiple genetic backgrounds (Feemster et al., 2023; Griffith et al., 2024; Hanquet et al., 2022; Ricketson et al., 2018; Ricketson et al., 2022; Wasserman et al., 2021).

Our study builds upon and complements previous Canadian reports of pneumococcal population structure and vaccine impact, including provincial and national surveillance efforts such as the SAVE study (Golden et al., 2023a). However, prior studies have largely focused on selected isolates across age groups, with limited inclusion of pediatric-specific, longitudinal, population-based genomic data. Our dataset is unique in its exhaustive inclusion of every pediatric IPD isolate in Calgary over 18 years and all pediatric isolates in Toronto over an 8-year period, coupled with whole-genome sequencing and serotype data. This level of granularity is not available in previous Canadian surveillance efforts.

A key observation of our study was the sustained prevalence of serotype 19A despite its inclusion in PCV13. This aligns with findings from other geographies where serotype 19A continues to contribute substantially to IPD burden (Corcoran et al., 2021; Griffith et al., 2024; Mott et al., 2019; Rockett et al., 2018; Rodriguez-Ruiz et al., 2024; Zeng et al., 2023). Phylogenomic analyses have shown that some serotype 19A lineages undergo frequent genetic exchange, including capsular switching, effectively evading PCV13-induced immunity, while often carrying AMR determinants that enable them to thrive under antibiotic pressure (Croucher et al., 2011; Sari et al., 2024; Wyres et al., 2013). Our genomic analyses revealed that serotype 19A isolates in both Calgary and Toronto belonged to multiple GPSCs, demonstrating that its persistence was not driven by a single lineage but rather by the parallel maintenance of multiple lineages across the study period. Importantly, we identified serotype 19A IPD cases in children who had received complete PCV13 vaccination, confirming that vaccine escape was not limited to under-vaccinated populations.

Our analyses also showed that many of the serotype 19A isolates were associated with increased penicillin MICs and the presence of macrolide resistance genes, such as *ermB* and *mefA*, further highlighting the capacity of this serotype to accumulate AMR determinants. This genetic plasticity, i.e., the ability to accumulate resistance and evade immunity through recombination and horizontal gene transfer, combined with the presence of multiple co-circulating lineages, likely contributed to the ability of serotype 19A to evade vaccine-induced immunity and persist over time. These findings reinforce the notion, previously described in Canadian and global studies (de Miguel et al., 2023; Liñares et al., 2010; Lo et al., 2019; Lo et al., 2022; Sandoval et al., 2024; Skoczyńska et al., 2015) that serotype 19A represents a paradigm of pneumococcal adaptability through both capsular switching and AMR gene acquisition. By documenting the long-term, multi-lineage persistence of serotype 19A in two major Canadian pediatric populations, our data extend and strengthen this evidence.

The adaptive capacity of the pneumococcus is driven in part by its high recombination rate (Bentley et al., 2006; Croucher et al., 2011). In addition to the multiplicity of GPSCs associated with serotype 19A, our data revealed that several GPSCs initially linked to vaccine-included serotypes (e.g., 19F, 18C, or 9V) later re-emerged under NVT capsules, likely due to capsular switching (Sari et al., 2024; Wyres et al., 2013). These events illustrate how pre-existing genetic backgrounds can evade PCV-induced immunity by acquiring NVT capsules, contributing to the ongoing diversification and persistence of pneumococcal genetic lineages. Although the frequency of capsule-switching events varies across regions and lineages, prior studies have shown that such strains can disseminate widely and evade vaccine-induced immunity (Wyres et al., 2013). Our genomic data provide clear evidence of these events in Canada, with multiple GPSCs initially associated with vaccine-included serotypes persisting or re-emerging under NVT capsules in both Calgary and Toronto. This underscores the ongoing risk of vaccine escape through capsular switching and the potential for virulent genetic backgrounds to be maintained despite vaccination. Close genomic surveillance is therefore essential to detect convergent trends and anticipate shifts in pneumococcal populations (Croucher et al., 2014). However, the evolutionary dynamics underlying these patterns in Canadian settings remain incompletely understood and warrant further longitudinal and functional investigation.

PCV13 has provided limited and potentially short-lived protection against serotype 3 IPD (Griffith et al., 2024; Lo et al., 2019; Ricketson et al., 2022). Unlike other vaccine-included serotypes that persisted through capsular switching, serotype 3 isolates in our study were consistently associated with a single lineage, GPSC12. This pattern suggests that the continued prevalence of serotype 3 may result from intrinsic properties of this lineage, such as resistance to immune clearance, rather than from horizontal gene transfer (Gladstone et al., 2021; Lo et al., 2022). Previous research has proposed that the serotype 3 capsule may be poorly immunogenic, leading to suboptimal vaccine responses (Gladstone et al., 2021). Our data support this hypothesis: serotype 3 accounted for 9.1% of pediatric IPD cases in Calgary and 8.3% in Toronto during P3. Phylogenetic analysis (Figure 2) further confirmed that serotype 3 isolates exclusively clustered within GPSC12, with no evidence of recent capsular switching. Together with the persistence of serotype 19A discussed above, the continued circulation of serotype 3 underscores the limitations of PCV13 in fully suppressing certain vaccine-included serotypes. In contrast, serotype 7F is typically considered immunogenic and has declined substantially in most populations post-PCV13 (Griffith et al., 2024; Lo et al., 2019; Ricketson et al., 2022). In our dataset, its continued presence in GPSC15 likely reflects low-level background circulation, rather than evidence of vaccine evasion.

Beyond these refractory VTs, our data revealed a broader epidemiologic shift toward emerging NVTs as major contributors to pediatric IPD. While serotype 22F, though present in both Calgary and Toronto, showed no significant difference in proportion, other NVTs exhibited notable regional variability. Specifically, serotype 33F was observed exclusively in Calgary, whereas serotype 15B was more prevalent in Toronto. These patterns are consistent with global reports of geographic variability in serotype distribution (Balsells et al., 2017; Weinberger et al., 2011) and likely reflect differences in local epidemiology potentially influenced by PCV implementation strategies, antimicrobial use, and unmeasured host population factors—such as household structure, comorbidities, travel history, and immigration. The accumulating evidence suggests that replacement serotypes, particularly 22F and 33F, can cause severe invasive disease and may harbor AMR determinants (Grant et al., 2023). Our findings align with these observations, highlighting the importance of continued genomic surveillance of emerging NVTs. Notably, the age-specific distribution of serotypes such as 15B/C, 22F, and 33F, and particularly their prominence among younger children, may help inform future decisions by NACI about the use of higher-valency conjugate vaccines (e.g., PCV15, PCV20) in pediatric populations.

Regional differences in vaccine schedules and implementation strategies may also have influenced patterns of NVT expansion. Although Alberta and Ontario both followed national guidance by NACI, variations in the timing and rollout of PCV7, the brief use of PCV10 in Ontario, and the subsequent adoption of PCV13 likely contributed to differences in local serotype dynamics. In our study, serotype 19A remained prevalent in both provinces but was more frequent in Toronto, possibly reflecting variations in catch-up programs, vaccine uptake, or antibiotic prescribing practices, as previously observed in Quebec and other regions (Deceuninck et al., 2024; Golden et al., 2023b). Similar patterns of resistant NVT lineages replacing previously dominant VT strains have been reported in Canada and internationally, although the specific serotype-genotype combinations vary by location (Lo et al., 2022).

Increased use of higher-valent PCVs, such as PCV15 and PCV20, may help to limit the spread of certain NVT lineages (National Advisory Committee on Immunization, 2024). However, whether these vaccines will fully address the continued emergence of other NVTs, particularly those prone to capsular switching, remains uncertain (Choi et al., 2024). Moreover, cost-effectiveness analyses from other settings have cautioned that expanding the number of covered serotypes does not guarantee eradication of IPD, given the unpredictable nature of serotype dynamics and capsule switching (Huang et al., 2024). Thus, in addition to expanding vaccine coverage, ongoing coordinated surveillance at both provincial and national levels is essential to monitor serotype and lineage shifts, assess the impact of vaccine implementation strategies, and guide future vaccination policies. Further research is also needed to clarify the relative contributions of vaccine schedules, antibiotic use, and demographic factors to local epidemiologic trends.

Pediatric pneumococcal vaccination programs have effectively reduced vaccine-type (VT) serotypes in children, with indirect benefits reported in adults through herd immunity (Miller et al., 2011; Weinberger et al., 2011). In our analysis, adult IPD serotype trends largely mirrored pediatric dynamics in both study regions. In Calgary, the post-PCV13 period was characterized by increases in serotypes 3, 8, and 19A among adults aged ≥50 years—serotypes also commonly found in pediatric cases during this period (Table 2 and Supplementary Figure 4). In Ontario, adult IPD during the same period was dominated by serotypes 3 and 22F, both of which were frequent among pediatric cases from Toronto. These findings align with prior Canadian and international studies reporting indirect protection conferred by pediatric PCV programs (Ciruela et al., 2019; Flem et al., 2024; Nasreen et al., 2024). However, our study was not specifically designed to assess adult disease dynamics, and limitations of the adult data, particularly in Ontario, warrant cautious interpretation. Ongoing, age-stratified genomic surveillance will be important to monitor these trends and to assess the broader impact of pediatric vaccination on adult pneumococcal disease, particularly in the context of recent recommendations to use PCV15 and PCV20 in adult populations (National Advisory Committee on Immunization, 2024; Ricketson and Kellner, 2023).

Our analysis revealed clear temporal trends in AMR among pediatric IPD isolates from Calgary. Macrolide resistance increased markedly over time, rising from 7.4% in the pre-vaccine period to 29.3% in the PCV13 era (Table 3). This increase reflected the accumulation of macrolide resistance determinants across multiple lineages and serotypes, rather than clonal expansion of a single resistant strain. Elevated erythromycin MICs were detected in several GPSCs, notably those associated with serotypes 19A, 33F, and 6A (Figure 5). These patterns are consistent with recent Canadian surveillance data reporting a progressive rise in macrolide resistance, particularly among pediatric invasive pneumococcal isolates (Griffith et al., 2024; Zhanel et al., 2023). Similar trends have been reported in earlier US datasets (Metcalf et al., 2016) and in other regions (Watkins et al., 2022).

In contrast, penicillin non-susceptibility remained infrequent in our dataset and was largely confined to specific lineages. Moderate increases in penicillin MICs were detected in GPSC4 and GPSC27 (serotype 19A) and serotype 19F, whereas serotype 3 (GPSC12) and serotype 22F (GPSC19) maintained low MICs across all periods (Figure 6). These findings are consistent with national surveillance reports indicating that penicillin resistance in invasive pneumococcal isolates has remained stable and low over time in Canadian and US populations (Griffith et al., 2024; Metcalf et al., 2016). Together, our results highlight the importance of integrating AMR monitoring within genomic surveillance frameworks.

In conclusion, by combining population-based, longitudinal genomic analysis with detailed epidemiologic data, we provide here a detailed portrait of pediatric pneumococcal evolution over nearly two decades in Calgary and complementary cross-sectional data from Toronto. Our results reinforce the dual impact of PCVs: the substantial reduction in VT disease, alongside the emergence and persistence of specific NVTs lineages. Importantly, we demonstrate that AMR, particularly macrolide resistance, has expanded across multiple genetic backgrounds, while *β*-lactam resistance has remained infrequent. These observations underscore the need for continuous, high-resolution genomic surveillance programs tailored to pediatric populations. Such efforts should aim to monitor ongoing serotype replacement, track the spread of resistant lineages, and evaluate the long-term impact of newer higher-valent PCVs and other preventive strategies. In addition, future investigations should prioritize the standardization and harmonization of GPSC-based tracking frameworks across regions, and assess how local vaccine schedules, antibiotic use, and demographic factors influence pneumococcal adaptation. As the Canadian context illustrates, local epidemiology, vaccine implementation strategies, and antibiotic use patterns can significantly influence pneumococcal population dynamics. An integrated approach combining genomic, epidemiologic, and AMR surveillance will be key to informing future vaccination policies and sustaining the gains achieved in pneumococcal disease prevention.

## Data Availability

The datasets presented in this study can be found in online repositories. The names of the repository/repositories and accession number(s) can be found in the Supplementary material.

## Footnotes

^1^ https://www.cdc.gov/pneumococcal/php/drug-resistance/index.html

^2^ https://www.pneumogen.net/gps/#/training#gpsc-assignment

## References

[ref1] Austrian R. (1976). The quellung reaction, a neglected microbiologic technique. Mount Sinai J. Med. 43, 699–709, PMID: 13297

[ref2] Balsells E. Guillot L. Nair H. Kyaw M. H. (2017). Serotype distribution of *Streptococcus pneumoniae* causing invasive disease in children in the post-PCV era: a systematic review and meta-analysis. PLoS One 12:e0177113. doi: 10.1371/journal.pone.0177113, PMID: 28486544 PMC5423631

[ref3] Bentley S. D. Aanensen D. M. Mavroidi A. Saunders D. Rabbinowitsch E. Collins M.. (2006). Genetic analysis of the capsular biosynthetic locus from all 90 pneumococcal serotypes. PLoS Genet. 2:e31. doi: 10.1371/journal.pgen.0020031, PMID: 16532061 PMC1391919

[ref4] Centers for Disease Control and Prevention (2021). Active bacterial Core surveillance report, emerging infections program network, Streptococcus pneumoniae, 2021. Atlanta, GA: CDC.

[ref5] Chen C. Cervero Liceras F. Flasche S. Sidharta S. Yoong J. Sundaram N.. (2019). Effect and cost-effectiveness of pneumococcal conjugate vaccination: a global modelling analysis. Lancet Glob. Health 7, e58–e67. doi: 10.1016/S2214-109X(18)30422-4, PMID: 30554762 PMC6293964

[ref6] Choi Y. H. Bertran M. Litt D. J. Ladhani S. N. Miller E. (2024). Potential impact of replacing the 13-valent pneumococcal conjugate vaccine with 15-valent or 20-valent pneumococcal conjugate vaccine in the 1 + 1 infant schedule in England: a modelling study. Lancet Public Health 9, e654–e663. doi: 10.1016/S2468-2667(24)00161-0, PMID: 39153492

[ref7] Ciruela P. Broner S. Izquierdo C. Pallarés R. Muñoz-Almagro C. Hernández S.. (2019). Indirect effects of paediatric conjugate vaccines on invasive pneumococcal disease in older adults. Int. J. Infect. Dis. 86, 122–130. doi: 10.1016/j.ijid.2019.06.030, PMID: 31283992

[ref8] CLSI (2019). Performance standards for antimicrobial susceptibility testing. Wayne, PA: Clinical and Laboratory Standards Institute.

[ref9] Coil D. Jospin G. Darling A. E. (2015). A5-miseq: an updated pipeline to assemble microbial genomes from Illumina MiSeq data. Bioinf (Oxf) 31, 587–589. doi: 10.1093/bioinformatics/btu661, PMID: 25338718

[ref10] Corcoran M. Mereckiene J. Cotter S. Murchan S. Lo S. W. McGee L.. (2021). Using genomics to examine the persistence of *Streptococcus pneumoniae* serotype 19A in Ireland and the emergence of a sub-clade associated with vaccine failures. Vaccine 39, 5064–5073. doi: 10.1016/j.vaccine.2021.06.017, PMID: 34301430

[ref11] Croucher N. J. Chewapreecha C. Hanage W. P. Harris S. R. McGee L. van der Linden M.. (2014). Evidence for soft selective sweeps in the evolution of pneumococcal multidrug resistance and vaccine escape. Genome Biol. Evol. 6, 1589–1602. doi: 10.1093/gbe/evu120, PMID: 24916661 PMC4122920

[ref12] Croucher N. J. Finkelstein J. A. Pelton S. I. Mitchell P. K. Lee G. M. Parkhill J.. (2013). Population genomics of post-vaccine changes in pneumococcal epidemiology. Nat. Genet. 45, 656–663. doi: 10.1038/ng.2625, PMID: 23644493 PMC3725542

[ref13] Croucher N. J. Harris S. R. Fraser C. Quail M. A. Burton J. van der Linden M.. (2011). Rapid pneumococcal evolution in response to clinical interventions. Science 331, 430–434. doi: 10.1126/science.1198545, PMID: 21273480 PMC3648787

[ref14] Croucher N. J. Page A. J. Connor T. R. Delaney A. J. Keane J. A. Bentley S. D.. (2015). Rapid phylogenetic analysis of large samples of recombinant bacterial whole genome sequences using gubbins. Nucleic Acids Res. 43:e15. doi: 10.1093/nar/gku1196, PMID: 25414349 PMC4330336

[ref15] de Miguel S. Pérez-Abeledo M. Ramos B. García L. Arce A. Martínez-Arce R.. (2023). Distribution of multidrug-resistant invasive serotypes of *Streptococcus pneumoniae* during the period 2007-2021 in Madrid, Spain. Antibiotics 12:342. doi: 10.3390/antibiotics12020342, PMID: 36830253 PMC9951976

[ref16] Deceuninck G. Brousseau N. Lefebvre B. Quach C. Tapiero B. Bui Y. G.. (2024). Effectiveness of the ten- and thirteen-valent pneumococcal conjugate vaccines to prevent serotype 19A invasive pneumococcal disease in Quebec, Canada. A Canadian immunization research network (CIRN) study. Vaccine 42:126379. doi: 10.1016/j.vaccine.2024.126379, PMID: 39332237

[ref17] Deng X. Memari N. Teatero S. Athey T. Isabel M. Mazzulli T.. (2016). Whole-genome sequencing for surveillance of invasive pneumococcal diseases in Ontario, Canada: rapid prediction of genotype, antibiotic resistance and characterization of emerging serotype 22F. Front. Microbiol. 7:2099. doi: 10.3389/fmicb.2016.02099, PMID: 28082965 PMC5187366

[ref18] Desai S. McGeer A. Quach-Thanh C. Elliott D. approved by NACI (2010). Update on the use of conjugate pneumococcal vaccines in childhood: an advisory committee statement (ACS) national advisory committee on immunization (NACI)†. Can. Commun. Dis. Rep. Relev. Mal. Transm. Can. 36, 1–21. doi: 10.14745/ccdr.v36i00a12, PMID: 31697280 PMC6802447

[ref19] Duke J. A. Avci F. Y. (2023). Emerging vaccine strategies against the incessant pneumococcal disease. NPJ Vaccines 8:122. doi: 10.1038/s41541-023-00715-w, PMID: 37591986 PMC10435554

[ref20] Duvvuri V. R. Deng X. Teatero S. Memari N. Athey T. Fittipaldi N.. (2016). Population structure and drug resistance patterns of emerging non-PCV-13 *Streptococcus pneumoniae* serotypes 22F, 15A, and 8 isolated from adults in Ontario, Canada. Inf. Genetics Evol. J. Mol. Epidemiol. Inf. Dis. 42, 1–8. doi: 10.1016/j.meegid.2016.04.007, PMID: 27071529

[ref21] Facklam R. R. Breiman R. F. (1991). Current trends in bacterial respiratory pathogens. Am. J. Med. 91, 3S–11S. doi: 10.1016/0002-9343(91)90301-d 1767804

[ref22] Feemster K. Weaver J. Buchwald U. Banniettis N. Cox K. S. McIntosh E. D.. (2023). Pneumococcal vaccine breakthrough and failure in infants and children: a narrative review. Vaccine 11:1750. doi: 10.3390/vaccines11121750, PMID: 38140155 PMC10747311

[ref23] Flem E. Mouawad C. Palmu A. A. Platt H. Johnson K. D. McIntosh E. D.. (2024). Indirect protection in adults ≥18 years of age from pediatric pneumococcal vaccination: a review. Expert Rev. Vaccines 23, 997–1010. doi: 10.1080/14760584.2024.2416229, PMID: 39435466

[ref24] Ganaie F. Saad J. S. McGee L. van Tonder A. J. Bentley S. D. Lo S. W.. (2020). A new pneumococcal capsule type, 10D, is the 100th serotype and has a large *cps* fragment from an oral streptococcus. MBio 11:e00937-20. doi: 10.1128/mBio.00937-20, PMID: 32430472 PMC7240158

[ref25] Gladstone R. A. Bojang E. Hart J. Harding-Esch E. M. Mabey D. Sillah A.. (2021). Mass drug administration with azithromycin for trachoma elimination and the population structure of *Streptococcus pneumoniae* in the nasopharynx. Clin. Microbiol. Infect. 27, 864–870. doi: 10.1016/j.cmi.2020.07.039, PMID: 32750538 PMC8203556

[ref26] Gladstone R. A. Lo S. W. Lees J. A. Croucher N. J. van Tonder A. J. Corander J.. (2019). International genomic definition of pneumococcal lineages, to contextualise disease, antibiotic resistance and vaccine impact. EBioMedicine 43, 338–346. doi: 10.1016/j.ebiom.2019.04.021, PMID: 31003929 PMC6557916

[ref27] Golden A. R. Adam H. J. Karlowsky J. A. Baxter M. Schellenberg J. Martin I.. (2023a). Genomic investigation of the most common *Streptococcus pneumoniae* serotypes causing invasive infections in Canada: the SAVE study, 2011-2020. J. Antimicrob. Chemother. 78, i26–i36. doi: 10.1093/jac/dkad067, PMID: 37130587

[ref28] Golden A. R. Lefebvre B. Deceuninck G. Brousseau N. De Wals P. Quach C.. (2023b). Clonal diversity of *Streptococcus pneumoniae* serotype 19A collected from children < 5 years old in Québec, Canada, 2016-2021. Vaccine 41, 6612–6618. doi: 10.1016/j.vaccine.2023.09.042, PMID: 37758569

[ref29] Grant L. R. Slack M. P. E. Theilacker C. Vojicic J. Dion S. Reinert R. R.. (2023). Distribution of serotypes causing invasive pneumococcal disease in children from high-income countries and the impact of pediatric pneumococcal vaccination. Clin. Infect. Dis. 76, e1062–e1070. doi: 10.1093/cid/ciac475, PMID: 35789262 PMC9907512

[ref30] Griffith A. Golden A. R. Lefebvre B. McGeer A. Tyrrell G. J. Zhanel G. G.. (2024). Invasive pneumococcal disease surveillance in Canada, 2021-2022. Canada Commun. Dis. Rep. 50, 121–134. doi: 10.14745/ccdr.v50i05a02, PMID: 38835503 PMC11147492

[ref31] Hackel M. Lascols C. Bouchillon S. Hilton B. Morgenstern D. Purdy J. (2013). Serotype prevalence and antibiotic resistance in *Streptococcus pneumoniae* clinical isolates among global populations. Vaccine 31, 4881–4887. doi: 10.1016/j.vaccine.2013.07.054, PMID: 23928466

[ref32] Hanage W. P. Huang S. S. Lipsitch M. Bishop C. J. Godoy D. Pelton S. I.. (2007). Diversity and antibiotic resistance among nonvaccine serotypes of *Streptococcus pneumoniae* carriage isolates in the post-heptavalent conjugate vaccine era. J. Infect. Dis. 195, 347–352. doi: 10.1086/510249 17205472

[ref33] Hanquet G. Krizova P. Dalby T. Ladhani S. N. Nuorti J. P. Danis K.. (2022). Serotype replacement after introduction of 10-Valent and 13-Valent pneumococcal conjugate vaccines in 10 countries, Europe. Emerg. Inf. Dis. 28, 137–138. doi: 10.3201/eid2801.210734, PMID: 34932457 PMC8714201

[ref34] Huang M. Weaver J. P. Elbasha E. Weiss T. Banniettis N. Feemster K.. (2024). Cost-effectiveness analysis of routine childhood immunization with 20-valent versus 15-valent pneumococcal conjugate vaccines in Germany. Vaccine 12:1045. doi: 10.3390/vaccines12091045, PMID: 39340075 PMC11435687

[ref35] Kellner J. D. Vanderkooi O. G. MacDonald J. Church D. L. Tyrrell G. J. Scheifele D. W. (2009). Changing epidemiology of invasive pneumococcal disease in Canada, 1998-2007: update from the Calgary-area *Streptococcus pneumoniae* research (CASPER) study. Clin. Infect. Dis. 49, 205–212. doi: 10.1086/599827, PMID: 19508165

[ref36] Kim S. H. Bae I. K. Park D. Lee K. Kim N. Y. Song S. A.. (2016). Serotype distribution and antimicrobial resistance of *Streptococcus pneumoniae* isolates causing invasive and noninvasive pneumococcal diseases in Korea from 2008 to 2014. Biomed. Res. Int. 2016:6950482. doi: 10.1155/2016/6950482, PMID: 27314035 PMC4904076

[ref37] Leal J. Vanderkooi O. G. Church D. L. Macdonald J. Tyrrell G. J. Kellner J. D. (2012). Eradication of invasive pneumococcal disease due to the seven-valent pneumococcal conjugate vaccine serotypes in Calgary, Alberta. Pediatr. Infect. Dis. J. 31, e169–e175. doi: 10.1097/INF.0b013e3182624a40, PMID: 22673137

[ref38] Lees J. A. Harris S. R. Tonkin-Hill G. Gladstone R. A. Lo S. W. Weiser J. N.. (2019). Fast and flexible bacterial genomic epidemiology with PopPUNK. Genome Res. 29, 304–316. doi: 10.1101/gr.241455.118, PMID: 30679308 PMC6360808

[ref39] Liñares J. Ardanuy C. Pallares R. Fenoll A. (2010). Changes in antimicrobial resistance, serotypes and genotypes in *Streptococcus pneumoniae* over a 30-year period. Clin. Microbiol. Infect. 16, 402–410. doi: 10.1111/j.1469-0691.2010.03182.x, PMID: 20132251

[ref40] Lo S. W. Gladstone R. A. van Tonder A. J. Lees J. A. du Plessis M. Benisty R.. (2019). Pneumococcal lineages associated with serotype replacement and antibiotic resistance in childhood invasive pneumococcal disease in the post-PCV13 era: an international whole-genome sequencing study. Lancet Infect. Dis. 19, 759–769. doi: 10.1016/S1473-3099(19)30297-X, PMID: 31196809 PMC7641901

[ref41] Lo S. W. Mellor K. Cohen R. Alonso A. R. Belman S. Kumar N.. (2022). Emergence of a multidrug-resistant and virulent *Streptococcus pneumoniae* lineage mediates serotype replacement after PCV13: an international whole-genome sequencing study. Lancet Microbe 3, e735–e743. doi: 10.1016/S2666-5247(22)00158-6, PMID: 35985351 PMC9519462

[ref42] Metcalf B. J. Chochua S. Gertz R. E. Jr. Li Z. Walker H. Tran T.. (2016). Using whole genome sequencing to identify resistance determinants and predict antimicrobial resistance phenotypes for year 2015 invasive pneumococcal disease isolates recovered in the United States. Clin. Microbiol. Infect. 22:1002. doi: 10.1016/j.cmi.2016.08.001, PMID: 27542334

[ref43] Miller E. Andrews N. J. Waight P. A. Slack M. P. George R. C. (2011). Herd immunity and serotype replacement 4 years after seven-valent pneumococcal conjugate vaccination in England and Wales: an observational cohort study. Lancet Infect. Dis. 11, 760–768. doi: 10.1016/S1473-3099(11)70090-1, PMID: 21621466

[ref44] Mostowy R. J. Holt K. E. (2018). Diversity-generating machines: genetics of bacterial sugar-coating. Trends Microbiol. 26, 1008–1021. doi: 10.1016/j.tim.2018.06.006, PMID: 30037568 PMC6249986

[ref45] Mott M. P. Caierão J. Cunha G. R. Del Maschi M. M. Pizzutti K. d'Azevedo P.. (2019). Emergence of serotype 19A *Streptococcus pneumoniae* after PCV10 associated with a ST320 in adult population, in Porto Alegre, Brazil. Epidemiol. Infect. 147:e93. doi: 10.1017/S0950268819000013, PMID: 30869012 PMC6518833

[ref46] Nasreen S. Wang J. Marra F. Kwong J. C. McGeer A. Sadarangani M.. (2024). Indirect impact of childhood 13-valent pneumococcal conjugate vaccine (PCV13) in Canadian older adults: a Canadian immunization research network (CIRN) retrospective observational study. Thorax 79, 861–869. doi: 10.1136/thorax-2023-220377, PMID: 38359926 PMC11347212

[ref47] National Advisory Committee on Immunization (2002). An advisory committee statement (ACS). National Advisory Committee on immunization (NACI). Statement on recommended use of pneumococcal conjugate vaccine. Canada Commun. Dis. Rep. 28, 1–32. 12728645

[ref48] National Advisory Committee on Immunization (2024). Recommendations for public health programs on the use of pneumococcal vaccines in children, including the use of 15-Valent and 20-Valent conjugate vaccines. [cat. No.: HP5-239/1-2024E-PDF]. Ottawa, ON: National Advisory Committee on Immunization.

[ref49] Page A. J. Cummins C. A. Hunt M. Wong V. K. Reuter S. Holden M. T.. (2015). Roary: rapid large-scale prokaryote pan genome analysis. Bioinf (Oxf) 31, 3691–3693. doi: 10.1093/bioinformatics/btv421, PMID: 26198102 PMC4817141

[ref50] Pilishvili T. Lexau C. Farley M. M. Hadler J. Harrison L. H. Bennett N. M.. (2010). Sustained reductions in invasive pneumococcal disease in the era of conjugate vaccine. J. Infect. Dis. 201, 32–41. doi: 10.1086/648593, PMID: 19947881

[ref51] Price M. N. Dehal P. S. Arkin A. P. (2010). FastTree 2--approximately maximum-likelihood trees for large alignments. PLoS One 5:e9490. doi: 10.1371/journal.pone.0009490, PMID: 20224823 PMC2835736

[ref52] R Core Team (2023). R: A language and environment for statistical computing. Vienna: R Foundation for Statistical Computing.

[ref53] Rennels M. B. Edwards K. M. Keyserling H. L. Reisinger K. S. Hogerman D. A. Madore D. V.. (1998). Safety and immunogenicity of heptavalent pneumococcal vaccine conjugated to CRM197 in United States infants. Pediatrics 101, 604–611. doi: 10.1542/peds.101.4.604, PMID: 9521941

[ref54] Ricketson L. J. Bettinger J. A. Sadarangani M. Halperin S. A. Kellner J. D. Canadian Immunization Monitoring Program, Active (IMPACT) Investigators (2022). Vaccine effectiveness of the 7-valent and 13-valent pneumococcal conjugate vaccines in Canada: an IMPACT study. Vaccine 40, 2733–2740. doi: 10.1016/j.vaccine.2022.03.048, PMID: 35351324

[ref55] Ricketson L. J. Conradi N. G. Vanderkooi O. G. Kellner J. D. (2018). Changes in the nature and severity of invasive pneumococcal disease in children before and after the seven-valent and thirteen-valent pneumococcal conjugate vaccine programs in Calgary, Canada. Pediatr. Infect. Dis. J. 37, 22–27. doi: 10.1097/INF.0000000000001709, PMID: 28737622

[ref56] Ricketson L. J. Kellner J. D. (2023). Changes in the incidence of invasive pneumococcal disease in Calgary, Canada, during the SARS-CoV-2 pandemic 2020-2022. Microorganisms 11:1333. doi: 10.3390/microorganisms11051333, PMID: 37317307 PMC10222282

[ref57] Rockett R. J. Oftadeh S. Bachmann N. L. Timms V. J. Kong F. Gilbert G. L.. (2018). Genome-wide analysis of *Streptococcus pneumoniae* serogroup 19 in the decade after the introduction of pneumococcal conjugate vaccines in Australia. Sci. Rep. 8:16969. doi: 10.1038/s41598-018-35270-1, PMID: 30446692 PMC6240094

[ref58] Rodriguez-Ruiz J. P. Xavier B. B. Stöhr W. van Heirstraeten L. Lammens C. Finn A.. (2024). High-resolution genomics identifies pneumococcal diversity and persistence of vaccine types in children with community-acquired pneumonia in the UK and Ireland. BMC Microbiol. 24:146. doi: 10.1186/s12866-024-03300-w, PMID: 38678217 PMC11055344

[ref59] Sandoval M. M. Ruvinsky S. Palermo M. C. Alconada T. Brizuela M. E. Wierzbicki E. R.. (2024). Antimicrobial resistance of *Streptococcus pneumoniae* from invasive pneumococcal diseases in Latin American countries: a systematic review and meta-analysis. Front. Public Health 12:1337276. doi: 10.3389/fpubh.2024.1337276, PMID: 38317800 PMC10839967

[ref60] Sari R. F. Fadilah F. Maladan Y. Sarassari R. Safari D. (2024). A narrative review of genomic characteristics, serotype, immunogenicity, and vaccine development of *Streptococcus pneumoniae* capsular polysaccharide. Clin. Exp. Vaccine Res. 13, 91–104. doi: 10.7774/cevr.2024.13.2.91, PMID: 38752009 PMC11091432

[ref61] Scheifele D. Halperin S. Pelletier L. Talbot J. (2000). Invasive pneumococcal infections in Canadian children, 1991-1998: implications for new vaccination strategies. Canadian paediatric society/Laboratory Centre for Disease Control Immunization Monitoring Program, active (IMPACT). Clin. Infect. Dis. 31, 58–64. doi: 10.1086/313923, PMID: 10913397

[ref62] Seemann T. (2014). Prokka: rapid prokaryotic genome annotation. Bioinf 30, 2068–2069. doi: 10.1093/bioinformatics/btu153, PMID: 24642063

[ref63] Skoczyńska A. Kuch A. Sadowy E. Waśko I. Markowska M. Ronkiewicz P.. (2015). Recent trends in epidemiology of invasive pneumococcal disease in Poland. Eur. J. Clin. Microbiol. Infect. Dis. 34, 779–787. doi: 10.1007/s10096-014-2283-8, PMID: 25475124

[ref64] Tyrrell G. J. Lovgren M. Chui N. Minion J. Garg S. Kellner J. D.. (2009). Serotypes and antimicrobial susceptibilities of invasive *Streptococcus pneumoniae* pre- and post-seven valent pneumococcal conjugate vaccine introduction in Alberta, Canada, 2000-2006. Vaccine 27, 3553–3560. doi: 10.1016/j.vaccine.2009.03.063, PMID: 19464534

[ref65] Wasserman M. Chapman R. Lapidot R. Sutton K. Dillon-Murphy D. Patel S.. (2021). Twenty-year public health impact of 7- and 13-Valent pneumococcal conjugate vaccines in US children. Emerg. Infect. Dis. 27, 1627–1636. doi: 10.3201/eid2706.204238, PMID: 34013855 PMC8153862

[ref66] Watkins E. R. Kalizang'Oma A. Gori A. Gupta S. Heyderman R. S. (2022). Factors affecting antimicrobial resistance in *Streptococcus pneumoniae* following vaccination introduction. Trends Microbiol. 30, 1135–1145. doi: 10.1016/j.tim.2022.06.001, PMID: 35843855

[ref67] Weinberger D. M. Malley R. Lipsitch M. (2011). Serotype replacement in disease after pneumococcal vaccination. Lancet 378, 1962–1973. doi: 10.1016/S0140-6736(10)62225-8, PMID: 21492929 PMC3256741

[ref68] Wilson M. R. Wasserman M. D. Breton M. C. Peloquin F. Earnshaw S. R. McDade C.. (2020). Health and economic impact of routine pediatric pneumococcal immunization programs in Canada: a retrospective analysis. Infect. Dis. Ther. 9, 341–353. doi: 10.1007/s40121-020-00294-6, PMID: 32270372 PMC7237628

[ref69] Wyres K. L. Lambertsen L. M. Croucher N. J. McGee L. von Gottberg A. Liñares J.. (2013). Pneumococcal capsular switching: a historical perspective. J. Infect. Dis. 207, 439–449. doi: 10.1093/infdis/jis703, PMID: 23175765 PMC3537446

[ref70] Yildirim I. Little B. A. Finkelstein J. Lee G. Hanage W. P. Shea K.. (2017). Surveillance of pneumococcal colonization and invasive pneumococcal disease reveals shift in prevalent carriage serotypes in Massachusetts' children to relatively low invasiveness. Vaccine 35, 4002–4009. doi: 10.1016/j.vaccine.2017.05.077, PMID: 28645717

[ref71] Zeng Y. Song Y. Cui L. Wu Q. Wang C. Coelho A. C.. (2023). Phylogenomic insights into evolutionary trajectories of multidrug resistant *S. pneumoniae* CC271 over a period of 14 years in China. Genome Med. 15:46. doi: 10.1186/s13073-023-01200-8, PMID: 37403170 PMC10318735

[ref72] Zhanel G. G. Lynch J. P. Adam H. J. (2023). *Streptococcus pneumoniae* serotyping and antimicrobial susceptibility: assessment for vaccine efficacy in Canada after the introduction of PCV13. J. Antimicrob. Chemother. 78, i2–i7. doi: 10.1093/jac/dkad064, PMID: 37130585

