# Supplementary material for: Evolving genomic landscape of pediatric pneumococcus in two Canadian urban centers following conjugate vaccination
Source: Front Microbiol. 2025 Aug 18;16:1642658. doi: 10.3389/fmicb.2025.1642658 (PMC12400966; doi:10.3389/fmicb.2025.1642658)
Supplement: Supplementary file 1 [file Table_1.DOCX]

# Supplementary Table 1. Population under surveillance and number of IPD isolates included in this study.

| Surveillance Program | Population under surveillance | | | Pediatric IPD | Adult IPD ^a^ |
| --- | --- | --- | --- | --- | --- |
|  | 1998 | 2009 | 2016 |  |  |
| CASPER (active surveillance, Calgary) | 888,432 |  | 1,392,609 | 338 | 261 |
| TIBDN (active surveillance, Toronto) | NA | 3,943,510 | 4,250,707 | 480 |  |
| Public Health Ontario (passive surveillance) ^b^ |  |  |  |  | 208 |
| Total |  |  |  | 818 | 469 |

^a^ Subset of total adult IPD in each region, representative of serotype distribution.

^b^ Only genome and limited patient data (no isolates) available to this study. The area covered in the province of Ontario.
