# Supplementary material for: Evolving genomic landscape of pediatric pneumococcus in two Canadian urban centers following conjugate vaccination
Source: Front Microbiol. 2025 Aug 18;16:1642658. doi: 10.3389/fmicb.2025.1642658 (PMC12400966; doi:10.3389/fmicb.2025.1642658)
Supplement: Supplementary file 2 [file Table_2.DOCX]

**Table S2. List of pediatric and adult invasive pneumococcal disease (IPD) isolates included in this study, with associated basic genome metadata.**

| Isolate name | Surveillance program | City or province | Age group | Serotype | GPSC ^a^ | DOC ^b^ | Genome assembly length (bp) | Number of Contigs ^c^ | N50 ^d^ | SRA Accession number ^e^ |
| --- | --- | --- | --- | --- | --- | --- | --- | --- | --- | --- |
| CSPN0001 | CASPER | Calgary | Pediatric | 14 | GPSC39 | 47.77 | 2,055,256 | 131 | 33,777 | SAMN48917514 |
| CSPN0003 | CASPER | Calgary | Pediatric | 14 | GPSC39 | 49.44 | 2,068,871 | 75 | 47,825 | SAMN48917516 |
| CSPN0002 | CASPER | Calgary | Pediatric | 23F | GPSC7 | 47.03 | 2,058,506 | 52 | 90,730 | SAMN48917515 |
| CSPN0005 | CASPER | Calgary | Pediatric | 18C | GPSC3 | 45.53 | 2,062,939 | 45 | 112,386 | SAMN48917518 |
| CSPN0004 | CASPER | Calgary | Pediatric | 23F | GPSC14 | 51.09 | 2,172,446 | 134 | 28,980 | SAMN48917517 |
| CSPN0006 | CASPER | Calgary | Pediatric | 14 | GPSC39 | 50.76 | 2,110,199 | 121 | 38,805 | SAMN48917519 |
| CSPN0008 | CASPER | Calgary | Pediatric | 14 | GPSC39 | 58.67 | 2,108,804 | 96 | 50,055 | SAMN48917521 |
| CSPN0007 | CASPER | Calgary | Pediatric | 14 | GPSC18 | 51.68 | 2,117,963 | 107 | 47,311 | SAMN48917520 |
| CSPN0011 | CASPER | Calgary | Pediatric | 4 | GPSC27 | 60.09 | 2,073,185 | 94 | 41,939 | SAMN48917524 |
| CSPN0009 | CASPER | Calgary | Pediatric | 14 | GPSC39 | 49.59 | 2,133,595 | 118 | 46,752 | SAMN48917522 |
| CSPN0010 | CASPER | Calgary | Pediatric | 18C | GPSC50 | 47.7 | 2,090,759 | 76 | 68,682 | SAMN48917523 |
| CSPN0013 | CASPER | Calgary | Pediatric | 14 | GPSC39 | 77.13 | 2,107,959 | 65 | 54,900 | SAMN48917526 |
| CSPN0012 | CASPER | Calgary | Pediatric | 14 | GPSC18 | 56.5 | 2,119,962 | 102 | 52,873 | SAMN48917525 |
| CSPN0014 | CASPER | Calgary | Pediatric | 14 | GPSC39 | 46.95 | 2,075,184 | 57 | 61,319 | SAMN48917527 |
| CSPN0015 | CASPER | Calgary | Pediatric | 23F | GPSC7 | 42.24 | 2,068,827 | 65 | 80,304 | SAMN48917528 |
| CSPN0016 | CASPER | Calgary | Pediatric | 4 | GPSC27 | 51.13 | 2,119,164 | 88 | 50,712 | SAMN48917529 |
| CSPN0017 | CASPER | Calgary | Pediatric | 18C | GPSC50 | 56.77 | 2,116,876 | 142 | 38,694 | SAMN48917530 |
| CSPN0018 | CASPER | Calgary | Pediatric | 14 | GPSC39 | 50.96 | 2,134,613 | 128 | 37,329 | SAMN48917531 |
| CSPN0026 | CASPER | Calgary | Pediatric | 6B | GPSC24 | 50.45 | 2,125,396 | 60 | 65,850 | SAMN48917539 |
| CSPN0019 | CASPER | Calgary | Pediatric | 15C | GPSC4 | 46.74 | 2,106,385 | 55 | 74,749 | SAMN48917532 |
| CSPN0020 | CASPER | Calgary | Pediatric | 19F | GPSC119 | 61.1 | 2,060,911 | 121 | 35,401 | SAMN48917533 |
| CSPN0021 | CASPER | Calgary | Pediatric | 9V | GPSC6 | 55.27 | 2,113,996 | 94 | 57,829 | SAMN48917534 |
| CSPN0023 | CASPER | Calgary | Pediatric | 23F | GPSC7 | 50.87 | 2,075,448 | 73 | 77,907 | SAMN48917536 |
| CSPN0022 | CASPER | Calgary | Pediatric | 23F | GPSC7 | 39.68 | 2,110,242 | 26 | 195,373 | SAMN48917535 |
| CSPN0024 | CASPER | Calgary | Pediatric | 3 | GPSC51 | 53.98 | 2,019,099 | 82 | 47,934 | SAMN48917537 |
| CSPN0025 | CASPER | Calgary | Pediatric | 14 | GPSC18 | 49.85 | 2,140,718 | 92 | 48,218 | SAMN48917538 |
| CSPN0028 | CASPER | Calgary | Pediatric | 14 | GPSC39 | 67.59 | 2,095,946 | 92 | 40,294 | SAMN48917541 |
| CSPN0027 | CASPER | Calgary | Pediatric | 9V | GPSC6 | 42.94 | 2,085,703 | 129 | 37,315 | SAMN48917540 |
| CSPN0029 | CASPER | Calgary | Pediatric | 6A | GPSC64 | 61.72 | 2,137,944 | 110 | 38,946 | SAMN48917542 |
| CSPN0030 | CASPER | Calgary | Pediatric | 6B | GPSC24 | 52.11 | 2,121,619 | 98 | 43,732 | SAMN48917543 |
| CSPN0031 | CASPER | Calgary | Pediatric | 23F | GPSC7 | 51.71 | 2,096,843 | 72 | 50,408 | SAMN48917544 |
| CSPN0033 | CASPER | Calgary | Pediatric | 14 | GPSC39 | 46.94 | 2,092,808 | 104 | 47,198 | SAMN48917546 |
| CSPN0036 | CASPER | Calgary | Pediatric | 14 | GPSC39 | 57.38 | 2,093,398 | 123 | 33,691 | SAMN48917549 |
| CSPN0037 | CASPER | Calgary | Pediatric | 14 | GPSC39 | 389.52 | 2,149,755 | 151 | 49,729 | SAMN48917550 |
| CSPN0034 | CASPER | Calgary | Pediatric | 19F | GPSC4 | 63.31 | 2,068,414 | 104 | 38,515 | SAMN48917547 |
| CSPN0035 | CASPER | Calgary | Pediatric | 23F | GPSC7 | 66.34 | 2,072,742 | 76 | 52,279 | SAMN48917548 |
| CSPN0032 | CASPER | Calgary | Pediatric | 6B | GPSC23 | 58.59 | 2,069,127 | 119 | 54,168 | SAMN48917545 |
| CSPN0041 | CASPER | Calgary | Pediatric | 22F | GPSC19 | 382.94 | 2,040,599 | 63 | 86,412 | SAMN48917553 |
| CSPN0040 | CASPER | Calgary | Pediatric | 14 | GPSC39 | 405.46 | 2,109,114 | 86 | 48,832 | SAMN48917552 |
| CSPN0039 | CASPER | Calgary | Pediatric | 18C | GPSC3 | 202.29 | 2,069,140 | 34 | 191,246 | SAMN48917551 |
| CSPN0042 | CASPER | Calgary | Pediatric | 18C | GPSC43 | 325.75 | 2,083,451 | 99 | 48,786 | SAMN48917554 |
| CSPN0043 | CASPER | Calgary | Pediatric | 4 | GPSC27 | 427.85 | 2,097,349 | 95 | 46,403 | SAMN48917555 |
| CSPN0044 | CASPER | Calgary | Pediatric | 14 | GPSC39 | 375.05 | 2,067,891 | 82 | 45,994 | SAMN48917556 |
| CSPN0045 | CASPER | Calgary | Pediatric | 14 | GPSC39 | 458.63 | 2,073,075 | 117 | 39,550 | SAMN48917557 |
| CSPN0047 | CASPER | Calgary | Pediatric | 9N | GPSC16 | 345.35 | 2,094,737 | 72 | 79,083 | SAMN48917559 |
| CSPN0046 | CASPER | Calgary | Pediatric | 18B | GPSC50 | 338.53 | 2,090,779 | 85 | 82,239 | SAMN48917558 |
| CSPN0049 | CASPER | Calgary | Pediatric | 23F | GPSC7 | 433.48 | 2,022,946 | 55 | 158,366 | SAMN48917561 |
| CSPN0310 | CASPER | Calgary | Adult | 14 | GPSC39 | 40.61 | 2,074,295 | 83 | 43,825 | SAMN48917814 |
| CSPN0309 | CASPER | Calgary | Adult | 4 | GPSC27 | 34.49 | 2,111,994 | 110 | 44,779 | SAMN48917813 |
| CSPN0048 | CASPER | Calgary | Pediatric | 14 | GPSC39 | 373.54 | 2,091,314 | 86 | 60,496 | SAMN48917560 |
| CSPN0050 | CASPER | Calgary | Pediatric | 18C | GPSC3 | 324.16 | 2,141,838 | 79 | 76,471 | SAMN48917562 |
| CSPN0312 | CASPER | Calgary | Adult | 14 | GPSC39 | 57.38 | 2,089,511 | 99 | 46,303 | SAMN48917816 |
| CSPN0311 | CASPER | Calgary | Adult | 4 | GPSC27 | 87.18 | 2,121,731 | 84 | 54,169 | SAMN48917815 |
| CSPN0053 | CASPER | Calgary | Pediatric | 19F | GPSC119 | 410.74 | 2,137,757 | 110 | 50,611 | SAMN48917565 |
| CSPN0052 | CASPER | Calgary | Pediatric | 14 | GPSC18 | 423.45 | 2,115,390 | 73 | 87,246 | SAMN48917564 |
| CSPN0051 | CASPER | Calgary | Pediatric | 6B | GPSC24 | 413.36 | 2,127,226 | 96 | 54,293 | SAMN48917563 |
| CSPN0313 | CASPER | Calgary | Adult | 3 | GPSC12 | 32.23 | 2,022,236 | 130 | 30,482 | SAMN48917817 |
| CSPN0315 | CASPER | Calgary | Adult | 4 | GPSC27 | 35.11 | 2,119,521 | 168 | 27,336 | SAMN48917818 |
| CSPN0055 | CASPER | Calgary | Pediatric | 19F | GPSC4 | 387.14 | 2,072,822 | 77 | 84,554 | SAMN48917567 |
| CSPN0054 | CASPER | Calgary | Pediatric | 6B | GPSC24 | 411.1 | 2,161,855 | 70 | 95,032 | SAMN48917566 |
| CSPN0316 | CASPER | Calgary | Adult | 14 | GPSC39 | 44.09 | 2,105,109 | 110 | 34,207 | SAMN48917819 |
| CSPN0057 | CASPER | Calgary | Pediatric | 19F | GPSC119 | 367.02 | 2,076,597 | 78 | 74,012 | SAMN48917569 |
| CSPN0056 | CASPER | Calgary | Pediatric | 19F | GPSC119 | 396.42 | 2,101,649 | 80 | 57,593 | SAMN48917568 |
| CSPN0318 | CASPER | Calgary | Adult | 7F | GPSC15 | 55.2 | 2,002,078 | 101 | 47,774 | SAMN48917820 |
| CSPN0058 | CASPER | Calgary | Pediatric | 14 | GPSC39 | 368.73 | 2,074,634 | 96 | 45,175 | SAMN48917570 |
| CSPN0060 | CASPER | Calgary | Pediatric | 19F | GPSC119 | 457.26 | 2,107,158 | 92 | 56,407 | SAMN48917572 |
| CSPN0059 | CASPER | Calgary | Pediatric | 19F | GPSC119 | 442.06 | 2,125,536 | 67 | 92,084 | SAMN48917571 |
| CSPN0319 | CASPER | Calgary | Adult | 4 | GPSC27 | 65.05 | 2,126,612 | 108 | 48,011 | SAMN48917821 |
| CSPN0062 | CASPER | Calgary | Pediatric | 18C | GPSC50 | 392.86 | 2,088,965 | 70 | 93,393 | SAMN48917573 |
| CSPN0065 | CASPER | Calgary | Pediatric | 14 | GPSC39 | 362.88 | 2,105,872 | 97 | 48,174 | SAMN48917576 |
| CSPN0064 | CASPER | Calgary | Pediatric | 19F | GPSC119 | 340.45 | 2,073,790 | 78 | 51,016 | SAMN48917575 |
| CSPN0063 | CASPER | Calgary | Pediatric | 23F | GPSC7 | 400.05 | 2,095,354 | 56 | 86,504 | SAMN48917574 |
| CSPN0066 | CASPER | Calgary | Pediatric | 38 | GPSC38 | 366.78 | 2,130,602 | 90 | 49,489 | SAMN48917577 |
| CSPN0067 | CASPER | Calgary | Pediatric | 7F | GPSC15 | 19.16 | 1,970,730 | 187 | 19,583 | SAMN48917578 |
| CSPN0320 | CASPER | Calgary | Adult | 3 | GPSC12 | 47.92 | 2,022,940 | 69 | 46,800 | SAMN48917822 |
| CSPN0069 | CASPER | Calgary | Pediatric | 14 | GPSC9 | 401.12 | 2,134,521 | 61 | 101,714 | SAMN48917579 |
| CSPN0070 | CASPER | Calgary | Pediatric | 18C | GPSC107 | 410.12 | 2,033,498 | 54 | 112,712 | SAMN48917580 |
| CSPN0071 | CASPER | Calgary | Pediatric | 18C | GPSC3 | 333.37 | 2,133,609 | 60 | 106,674 | SAMN48917581 |
| CSPN0322 | CASPER | Calgary | Adult | 14 | GPSC16 | 82.6 | 2,150,980 | 69 | 59,177 | SAMN48917824 |
| CSPN0321 | CASPER | Calgary | Adult | 14 | GPSC39 | 37.72 | 2,097,237 | 139 | 30,266 | SAMN48917823 |
| CSPN0073 | CASPER | Calgary | Pediatric | 33F | GPSC3 | 361.04 | 2,021,322 | 72 | 141,501 | SAMN48917583 |
| CSPN0075 | CASPER | Calgary | Pediatric | 3 | GPSC12 | 382.55 | 2,026,159 | 30 | 202,537 | SAMN48917585 |
| CSPN0072 | CASPER | Calgary | Pediatric | 19A | GPSC4 | 404.97 | 2,117,231 | 95 | 72,952 | SAMN48917582 |
| CSPN0074 | CASPER | Calgary | Pediatric | 18C | GPSC3 | 426.55 | 2,092,307 | 49 | 100,883 | SAMN48917584 |
| CSPN0324 | CASPER | Calgary | Adult | 4 | GPSC27 | 66.39 | 2,112,024 | 198 | 22,647 | SAMN48917826 |
| CSPN0323 | CASPER | Calgary | Adult | 14 | GPSC39 | 370.01 | 2,123,843 | 124 | 44,100 | SAMN48917825 |
| CSPN0076 | CASPER | Calgary | Pediatric | 14 | GPSC39 | 360.47 | 2,084,366 | 93 | 49,161 | SAMN48917586 |
| CSPN0077 | CASPER | Calgary | Pediatric | 6B | GPSC47 | 359.31 | 2,197,537 | 92 | 44,524 | SAMN48917587 |
| CSPN0326 | CASPER | Calgary | Adult | 4 | GPSC27 | 34.24 | 2,093,365 | 157 | 25,910 | SAMN48917828 |
| CSPN0325 | CASPER | Calgary | Adult | 14 | GPSC39 | 24.42 | 2,069,483 | 132 | 34,678 | SAMN48917827 |
| CSPN0086 | CASPER | Calgary | Pediatric | 23A | GPSC7 | 431.22 | 2,104,900 | 75 | 81,020 | SAMN48917596 |
| CSPN0085 | CASPER | Calgary | Pediatric | 19A | GPSC4 | 350.5 | 2,112,452 | 76 | 80,017 | SAMN48917595 |
| CSPN0082 | CASPER | Calgary | Pediatric | 4 | GPSC27 | 380.91 | 2,125,749 | 85 | 71,892 | SAMN48917592 |
| CSPN0084 | CASPER | Calgary | Pediatric | 14 | GPSC39 | 464.5 | 2,081,298 | 100 | 46,070 | SAMN48917594 |
| CSPN0079 | CASPER | Calgary | Pediatric | 14 | GPSC6 | 403.02 | 2,125,183 | 87 | 94,750 | SAMN48917589 |
| CSPN0078 | CASPER | Calgary | Pediatric | 6B | GPSC47 | 344.14 | 2,198,109 | 90 | 49,200 | SAMN48917588 |
| CSPN0080 | CASPER | Calgary | Pediatric | 17F | GPSC49 | 353.45 | 2,059,915 | 68 | 77,159 | SAMN48917590 |
| CSPN0081 | CASPER | Calgary | Pediatric | 6A | GPSC14 | 433.61 | 2,113,184 | 85 | 62,467 | SAMN48917591 |
| CSPN0328 | CASPER | Calgary | Adult | 8 | GPSC98 | 30.04 | 1,989,472 | 99 | 42,141 | SAMN48917830 |
| CSPN0327 | CASPER | Calgary | Adult | 3 | GPSC12 | 360.81 | 2,040,903 | 72 | 68,815 | SAMN48917829 |
| CSPN0083 | CASPER | Calgary | Pediatric | 6A | GPSC24 | 444.04 | 2,115,137 | 83 | 66,010 | SAMN48917593 |
| CSPN0091 | CASPER | Calgary | Pediatric | 14 | GPSC39 | 292.69 | 2,130,393 | 102 | 50,453 | SAMN48917601 |
| CSPN0087 | CASPER | Calgary | Pediatric | 14 | GPSC39 | 394.37 | 2,037,035 | 93 | 50,410 | SAMN48917597 |
| CSPN0089 | CASPER | Calgary | Pediatric | 14 | GPSC18 | 378.67 | 2,147,948 | 78 | 84,469 | SAMN48917599 |
| CSPN0088 | CASPER | Calgary | Pediatric | 6B | GPSC24 | 334.41 | 2,119,048 | 67 | 86,563 | SAMN48917598 |
| CSPN0092 | CASPER | Calgary | Pediatric | 18C | GPSC3 | 360.66 | 2,093,664 | 53 | 88,358 | SAMN48917602 |
| CSPN0093 | CASPER | Calgary | Pediatric | 18C | GPSC50 | 372.67 | 2,089,704 | 71 | 92,551 | SAMN48917603 |
| CSPN0090 | CASPER | Calgary | Pediatric | 14 | GPSC39 | 345.29 | 2,070,509 | 80 | 52,421 | SAMN48917600 |
| CSPN0330 | CASPER | Calgary | Adult | 14 | GPSC39 | 30.58 | 2,087,911 | 135 | 33,652 | SAMN48917832 |
| CSPN0329 | CASPER | Calgary | Adult | 9V | GPSC6 | 267.66 | 2,119,010 | 93 | 58,540 | SAMN48917831 |
| CSPN0094 | CASPER | Calgary | Pediatric | 18C | GPSC3 | 362.48 | 2,096,676 | 67 | 102,843 | SAMN48917604 |
| CSPN0095 | CASPER | Calgary | Pediatric | 4 | GPSC162 | 347.35 | 2,061,212 | 146 | 49,063 | SAMN48917605 |
| CSPN0096 | CASPER | Calgary | Pediatric | 22F | GPSC19 | 363.73 | 2,080,487 | 75 | 77,712 | SAMN48917606 |
| CSPN0331 | CASPER | Calgary | Adult | 8 | GPSC3 | 189.57 | 2,001,505 | 61 | 88,376 | SAMN48917833 |
| CSPN0097 | CASPER | Calgary | Pediatric | 14 | GPSC39 | 361.54 | 2,127,000 | 123 | 48,229 | SAMN48917607 |
| CSPN0332 | CASPER | Calgary | Adult | 4 | GPSC27 | 29.08 | 2,091,815 | 157 | 26,068 | SAMN48917834 |
| CSPN0098 | CASPER | Calgary | Pediatric | 4 | GPSC27 | 347.74 | 2,124,422 | 100 | 54,656 | SAMN48917608 |
| CSPN0101 | CASPER | Calgary | Pediatric | 18C | GPSC3 | 346.18 | 2,061,131 | 42 | 91,397 | SAMN48917611 |
| CSPN0100 | CASPER | Calgary | Pediatric | 6A | GPSC64 | 323.28 | 2,137,253 | 80 | 62,625 | SAMN48917610 |
| CSPN0099 | CASPER | Calgary | Pediatric | 3 | GPSC12 | 364.89 | 2,027,285 | 48 | 144,207 | SAMN48917609 |
| CSPN0106 | CASPER | Calgary | Pediatric | 14 | GPSC39 | 264.11 | 2,143,968 | 87 | 50,660 | SAMN48917616 |
| CSPN0104 | CASPER | Calgary | Pediatric | 18C | GPSC3 | 372.17 | 2,069,036 | 40 | 125,503 | SAMN48917614 |
| CSPN0102 | CASPER | Calgary | Pediatric | 6B | GPSC24 | 324.56 | 2,064,521 | 57 | 75,505 | SAMN48917612 |
| CSPN0105 | CASPER | Calgary | Pediatric | 9V | GPSC6 | 327.72 | 2,108,747 | 43 | 90,554 | SAMN48917615 |
| CSPN0103 | CASPER | Calgary | Pediatric | 18C | GPSC3 | 364.42 | 2,097,919 | 43 | 255,175 | SAMN48917613 |
| CSPN0333 | CASPER | Calgary | Adult | 14 | GPSC39 | 24.69 | 2,051,507 | 179 | 21,073 | SAMN48917835 |
| CSPN0110 | CASPER | Calgary | Pediatric | 6A | GPSC64 | 365.56 | 2,136,031 | 60 | 97,195 | SAMN48917620 |
| CSPN0107 | CASPER | Calgary | Pediatric | 14 | GPSC39 | 398.03 | 2,071,576 | 70 | 70,538 | SAMN48917617 |
| CSPN0108 | CASPER | Calgary | Pediatric | 19F | GPSC4 | 316.69 | 2,108,411 | 35 | 124,813 | SAMN48917618 |
| CSPN0109 | CASPER | Calgary | Pediatric | 1 | GPSC31 | 316.34 | 2,101,290 | 98 | 47,146 | SAMN48917619 |
| CSPN0111 | CASPER | Calgary | Pediatric | 9V | GPSC6 | 305.29 | 2,110,146 | 76 | 68,233 | SAMN48917621 |
| CSPN0112 | CASPER | Calgary | Pediatric | 6B | GPSC24 | 298.99 | 2,120,154 | 54 | 80,594 | SAMN48917622 |
| CSPN0335 | CASPER | Calgary | Adult | 14 | GPSC39 | 34.15 | 2,067,672 | 120 | 32,360 | SAMN48917837 |
| CSPN0336 | CASPER | Calgary | Adult | 14 | GPSC39 | 403.61 | 2,127,010 | 114 | 55,536 | SAMN48917838 |
| CSPN0334 | CASPER | Calgary | Adult | 8 | GPSC39 | 33.92 | 2,067,755 | 118 | 31,812 | SAMN48917836 |
| CSPN0113 | CASPER | Calgary | Pediatric | 22F | GPSC19 | 430.54 | 2,068,486 | 56 | 76,303 | SAMN48917623 |
| CSPN0117 | CASPER | Calgary | Pediatric | 14 | GPSC39 | 297.64 | 2,097,681 | 99 | 45,429 | SAMN48917626 |
| CSPN0115 | CASPER | Calgary | Pediatric | 6B | GPSC24 | 386.13 | 2,059,690 | 48 | 100,396 | SAMN48917624 |
| CSPN0116 | CASPER | Calgary | Pediatric | 23A | GPSC7 | 343.41 | 2,070,436 | 65 | 74,719 | SAMN48917625 |
| CSPN0337 | CASPER | Calgary | Adult | 14 | GPSC39 | 27.73 | 2,073,633 | 139 | 28,288 | SAMN48917839 |
| CSPN0118 | CASPER | Calgary | Pediatric | 19F | GPSCNA ^g^ | 367.62 | 2,093,968 | 94 | 69,623 | SAMN48917627 |
| CSPN0119 | CASPER | Calgary | Pediatric | 14 | GPSC39 | 335.64 | 2,099,453 | 102 | 46,125 | SAMN48917628 |
| CSPN0121 | CASPER | Calgary | Pediatric | 19F | GPSC119 | 314.01 | 2,118,323 | 92 | 50,213 | SAMN48917630 |
| CSPN0120 | CASPER | Calgary | Pediatric | 9V | GPSC6 | 356.88 | 2,117,317 | 84 | 62,449 | SAMN48917629 |
| CSPN0340 | CASPER | Calgary | Adult | 14 | GPSC39 | 282.92 | 2,072,054 | 84 | 51,152 | SAMN48917842 |
| CSPN0339 | CASPER | Calgary | Adult | 3 | GPSC12 | 23.67 | 2,019,930 | 194 | 20,038 | SAMN48917841 |
| CSPN0338 | CASPER | Calgary | Adult | 8 | GPSC98 | 163.25 | 1,991,266 | 53 | 82,984 | SAMN48917840 |
| CSPN0124 | CASPER | Calgary | Pediatric | 6B | GPSC47 | 290 | 2,203,304 | 73 | 77,317 | SAMN48917632 |
| CSPN0125 | CASPER | Calgary | Pediatric | 4 | GPSC27 | 366.03 | 2,115,480 | 87 | 48,639 | SAMN48917633 |
| CSPN0341 | CASPER | Calgary | Adult | 4 | GPSC27 | 277.35 | 2,125,552 | 108 | 49,301 | SAMN48917843 |
| CSPN0342 | CASPER | Calgary | Adult | 14 | GPSC39 | 27.42 | 2,093,525 | 174 | 29,852 | SAMN48917844 |
| CSPN0126 | CASPER | Calgary | Pediatric | 14 | GPSC39 | 350.3 | 2,111,387 | 86 | 49,245 | SAMN48917634 |
| CSPN0127 | CASPER | Calgary | Pediatric | 23F | GPSC7 | 310.21 | 2,086,222 | 47 | 131,523 | SAMN48917635 |
| CSPN0128 | CASPER | Calgary | Pediatric | 18C | GPSC3 | 413.44 | 2,061,375 | 37 | 133,573 | SAMN48917636 |
| CSPN0129 | CASPER | Calgary | Pediatric | 14 | GPSC18 | 262.9 | 2,062,188 | 52 | 130,511 | SAMN48917637 |
| CSPN0130 | CASPER | Calgary | Pediatric | 6A | GPSC64 | 381.92 | 2,136,005 | 62 | 85,573 | SAMN48917638 |
| CSPN0344 | CASPER | Calgary | Adult | 7F | GPSC15 | 239.28 | 1,992,815 | 81 | 59,634 | SAMN48917846 |
| CSPN0343 | CASPER | Calgary | Adult | 14 | GPSC6 | 159.26 | 2,141,046 | 64 | 85,953 | SAMN48917845 |
| CSPN0132 | CASPER | Calgary | Pediatric | 34 | GPSC45 | 343.93 | 2,102,344 | 49 | 139,075 | SAMN48917640 |
| CSPN0131 | CASPER | Calgary | Pediatric | 14 | GPSC39 | 495.69 | 2,114,145 | 153 | 37,606 | SAMN48917639 |
| CSPN0133 | CASPER | Calgary | Pediatric | 14 | GPSC39 | 144.58 | 2,147,907 | 141 | 40,556 | SAMN48917641 |
| CSPN0345 | CASPER | Calgary | Adult | 14 | GPSC39 | 27.53 | 2,093,807 | 118 | 35,726 | SAMN48917847 |
| CSPN0138 | CASPER | Calgary | Pediatric | 14 | GPSC39 | 295.8 | 2,071,670 | 82 | 46,103 | SAMN48917645 |
| CSPN0140 | CASPER | Calgary | Pediatric | 14 | GPSC39 | 318.59 | 2,120,036 | 92 | 47,866 | SAMN48917647 |
| CSPN0139 | CASPER | Calgary | Pediatric | 19F | GPSC4 | 311.4 | 2,110,861 | 69 | 82,971 | SAMN48917646 |
| CSPN0135 | CASPER | Calgary | Pediatric | 19F | GPSC11 | 172.11 | 2,109,631 | 132 | 42,411 | SAMN48917643 |
| CSPN0347 | CASPER | Calgary | Adult | 4 | GPSC27 | 466.78 | 2,089,491 | 85 | 59,801 | SAMN48917849 |
| CSPN0346 | CASPER | Calgary | Adult | 14 | GPSC3 | 33.57 | 2,063,617 | 137 | 39,210 | SAMN48917848 |
| CSPN0134 | CASPER | Calgary | Pediatric | 38 | GPSC38 | 215.83 | 2,137,623 | 84 | 50,049 | SAMN48917642 |
| CSPN0137 | CASPER | Calgary | Pediatric | 14 | GPSC39 | 251.16 | 2,069,896 | 102 | 40,919 | SAMN48917644 |
| CSPN0141 | CASPER | Calgary | Pediatric | 14 | GPSC16 | 206.26 | 2,148,972 | 148 | 48,411 | SAMN48917648 |
| CSPN0143 | CASPER | Calgary | Pediatric | 9V | GPSC6 | 261.71 | 2,122,337 | 91 | 58,562 | SAMN48917650 |
| CSPN0142 | CASPER | Calgary | Pediatric | 6A | GPSC64 | 125.42 | 2,121,150 | 65 | 73,594 | SAMN48917649 |
| CSPN0348 | CASPER | Calgary | Adult | 3 | GPSC12 | 488.96 | 2,023,076 | 63 | 96,874 | SAMN48917850 |
| CSPN0158 | CASPER | Calgary | Pediatric | 6A | GPSC24 | 222.49 | 2,113,707 | 99 | 48,702 | SAMN48917665 |
| CSPN0144 | CASPER | Calgary | Pediatric | 14 | GPSC39 | 162.81 | 2,089,240 | 108 | 42,508 | SAMN48917651 |
| CSPN0349 | CASPER | Calgary | Adult | 8 | GPSC3 | 201.98 | 2,038,561 | 57 | 103,944 | SAMN48917851 |
| CSPN0353 | CASPER | Calgary | Adult | 7F | GPSC15 | 176.46 | 1,990,521 | 76 | 69,693 | SAMN48917855 |
| CSPN0350 | CASPER | Calgary | Adult | 14 | GPSC39 | 234.65 | 2,098,150 | 125 | 39,055 | SAMN48917852 |
| CSPN0351 | CASPER | Calgary | Adult | 19A | GPSC4 | 296.85 | 2,163,910 | 79 | 69,254 | SAMN48917853 |
| CSPN0352 | CASPER | Calgary | Adult | 8 | GPSC3 | 199.98 | 2,001,304 | 47 | 99,939 | SAMN48917854 |
| CSPN0145 | CASPER | Calgary | Pediatric | 18C | GPSC3 | 82.81 | 2,099,002 | 82 | 59,258 | SAMN48917652 |
| CSPN0146 | CASPER | Calgary | Pediatric | 19F | GPSC119 | 85.61 | 2,081,504 | 102 | 48,345 | SAMN48917653 |
| CSPN0354 | CASPER | Calgary | Adult | 14 | GPSC16 | 25.15 | 2,145,052 | 151 | 33,374 | SAMN48917856 |
| CSPN0147 | CASPER | Calgary | Pediatric | 6B | GPSC24 | 143.81 | 2,119,860 | 103 | 56,823 | SAMN48917654 |
| CSPN0149 | CASPER | Calgary | Pediatric | 14 | GPSC39 | 149.19 | 2,082,573 | 143 | 34,922 | SAMN48917656 |
| CSPN0148 | CASPER | Calgary | Pediatric | 14 | GPSC39 | 186 | 2,074,804 | 86 | 49,115 | SAMN48917655 |
| CSPN0122 | CASPER | Calgary | Pediatric | 1 | GPSC50 | 305.29 | 2,095,943 | 63 | 91,964 | SAMN48917631 |
| CSPN0355 | CASPER | Calgary | Adult | 4 | GPSC27 | 407.01 | 2,090,779 | 74 | 52,306 | SAMN48917857 |
| CSPN0150 | CASPER | Calgary | Pediatric | 18C | GPSC3 | 157.64 | 2,099,233 | 43 | 131,160 | SAMN48917657 |
| CSPN0151 | CASPER | Calgary | Pediatric | 19F | GPSC119 | 153.1 | 2,108,003 | 123 | 45,513 | SAMN48917658 |
| CSPN0356 | CASPER | Calgary | Adult | 14 | GPSC39 | 473.69 | 2,069,172 | 84 | 59,134 | SAMN48917858 |
| CSPN0153 | CASPER | Calgary | Pediatric | 14 | GPSC39 | 231.44 | 2,108,474 | 108 | 37,409 | SAMN48917660 |
| CSPN0152 | CASPER | Calgary | Pediatric | 16F | GPSC135 | 301.45 | 2,090,265 | 87 | 64,174 | SAMN48917659 |
| CSPN0358 | CASPER | Calgary | Adult | 3 | GPSC12 | 165.52 | 2,028,785 | 79 | 68,552 | SAMN48917860 |
| CSPN0357 | CASPER | Calgary | Adult | 14 | GPSC39 | 298.16 | 2,069,851 | 105 | 48,526 | SAMN48917859 |
| CSPN0359 | CASPER | Calgary | Adult | 7F | GPSC15 | 30.7 | 1,992,873 | 148 | 30,465 | SAMN48917861 |
| CSPN0154 | CASPER | Calgary | Pediatric | 19F | GPSC4 | 113.99 | 2,073,384 | 61 | 68,881 | SAMN48917661 |
| CSPN0360 | CASPER | Calgary | Adult | 4 | GPSC27 | 513.59 | 2,092,226 | 90 | 50,348 | SAMN48917862 |
| CSPN0155 | CASPER | Calgary | Pediatric | 6B | GPSC24 | 255.49 | 2,165,422 | 101 | 57,580 | SAMN48917662 |
| CSPN0156 | CASPER | Calgary | Pediatric | 14 | GPSC39 | 153.86 | 2,076,456 | 129 | 33,264 | SAMN48917663 |
| CSPN0362 | CASPER | Calgary | Adult | 3 | GPSC12 | 290.59 | 2,026,750 | 69 | 83,572 | SAMN48917864 |
| CSPN0361 | CASPER | Calgary | Adult | 4 | GPSC27 | 342.18 | 2,120,486 | 108 | 55,517 | SAMN48917863 |
| CSPN0157 | CASPER | Calgary | Pediatric | 18C | GPSC3 | 148.81 | 2,102,100 | 65 | 61,472 | SAMN48917664 |
| CSPN0160 | CASPER | Calgary | Pediatric | 6B | GPSC24 | 272.43 | 2,126,899 | 70 | 80,473 | SAMN48917667 |
| CSPN0364 | CASPER | Calgary | Adult | 9V | GPSC6 | 397.27 | 2,146,812 | 81 | 84,790 | SAMN48917865 |
| CSPN0159 | CASPER | Calgary | Pediatric | 14 | GPSC39 | 105.95 | 2,103,509 | 99 | 42,349 | SAMN48917666 |
| CSPN0162 | CASPER | Calgary | Pediatric | 15A | GPSC324 | 266.49 | 2,118,193 | 127 | 48,026 | SAMN48917669 |
| CSPN0161 | CASPER | Calgary | Pediatric | 6B | GPSC24 | 58.83 | 2,123,249 | 182 | 24,972 | SAMN48917668 |
| CSPN0163 | CASPER | Calgary | Pediatric | 14 | GPSC39 | 90.45 | 2,077,236 | 111 | 34,254 | SAMN48917670 |
| CSPN0164 | CASPER | Calgary | Pediatric | 18C | GPSC3 | 148.92 | 2,101,028 | 97 | 62,225 | SAMN48917671 |
| CSPN0367 | CASPER | Calgary | Adult | 3 | GPSC12 | 262.18 | 2,021,183 | 58 | 105,229 | SAMN48917867 |
| CSPN0365 | CASPER | Calgary | Adult | 8 | GPSC98 | 24.21 | 1,991,059 | 139 | 28,869 | SAMN48917866 |
| CSPN0166 | CASPER | Calgary | Pediatric | 18B | GPSC3 | 192.65 | 2,100,405 | 73 | 71,230 | SAMN48917673 |
| CSPN0165 | CASPER | Calgary | Pediatric | 18C | GPSC3 | 259.42 | 2,093,626 | 50 | 90,668 | SAMN48917672 |
| CSPN0167 | CASPER | Calgary | Pediatric | 18C | GPSC3 | 232.47 | 2,072,457 | 48 | 102,724 | SAMN48917674 |
| CSPN0168 | CASPER | Calgary | Pediatric | 3 | GPSC12 | 102.26 | 2,027,823 | 61 | 69,479 | SAMN48917675 |
| CSPN0368 | CASPER | Calgary | Adult | 4 | GPSC27 | 273 | 2,093,774 | 112 | 40,814 | SAMN48917868 |
| CSPN0169 | CASPER | Calgary | Pediatric | 6B | GPSC24 | 112.48 | 2,125,019 | 74 | 74,653 | SAMN48917676 |
| CSPN0369 | CASPER | Calgary | Adult | 4 | GPSC27 | 347.71 | 2,156,521 | 75 | 52,907 | SAMN48917869 |
| CSPN0170 | CASPER | Calgary | Pediatric | 15A | GPSC140 | 149.28 | 2,109,125 | 119 | 39,655 | SAMN48917677 |
| CSPN0171 | CASPER | Calgary | Pediatric | 6A | GPSC24 | 121.8 | 2,061,008 | 86 | 51,984 | SAMN48917678 |
| CSPN0371 | CASPER | Calgary | Adult | 19A | GPSC4 | 125.4 | 2,118,042 | 109 | 40,309 | SAMN48917871 |
| CSPN0370 | CASPER | Calgary | Adult | 14 | GPSC39 | 253.13 | 2,095,490 | 87 | 48,953 | SAMN48917870 |
| CSPN0373 | CASPER | Calgary | Adult | 4 | GPSC27 | 28.8 | 2,113,879 | 158 | 28,424 | SAMN48917873 |
| CSPN0372 | CASPER | Calgary | Adult | 9V | GPSC6 | 401.65 | 2,091,853 | 59 | 75,938 | SAMN48917872 |
| CSPN0172 | CASPER | Calgary | Pediatric | 3 | GPSC12 | 30.5 | 2,019,909 | 162 | 33,674 | SAMN48917679 |
| CSPN0174 | CASPER | Calgary | Pediatric | 18C | GPSC50 | 58.67 | 2,144,780 | 141 | 40,993 | SAMN48917681 |
| CSPN0173 | CASPER | Calgary | Pediatric | 6B | GPSC24 | 299.21 | 2,119,178 | 49 | 126,065 | SAMN48917680 |
| CSPN0374 | CASPER | Calgary | Adult | 8 | GPSC98 | 27.14 | 1,989,132 | 172 | 20,535 | SAMN48917874 |
| CSPN0379 | CASPER | Calgary | Adult | 3 | GPSC12 | 419.01 | 2,021,943 | 47 | 86,524 | SAMN48917879 |
| CSPN0384 | CASPER | Calgary | Adult | 3 | GPSC12 | 283.09 | 2,022,679 | 43 | 85,676 | SAMN48917884 |
| CSPN0386 | CASPER | Calgary | Adult | 3 | GPSC12 | 315.79 | 2,019,743 | 36 | 93,145 | SAMN48917886 |
| CSPN0387 | CASPER | Calgary | Adult | 8 | GPSC98 | 288.73 | 1,990,016 | 47 | 71,432 | SAMN48917887 |
| CSPN0392 | CASPER | Calgary | Adult | 8 | GPSC98 | 120.66 | 1,993,455 | 92 | 47,220 | SAMN48917892 |
| CSPN0375 | CASPER | Calgary | Adult | 8 | GPSC98 | 199.08 | 1,989,293 | 60 | 87,449 | SAMN48917875 |
| CSPN0377 | CASPER | Calgary | Adult | 8 | GPSC98 | 324.77 | 1,991,865 | 47 | 100,360 | SAMN48917877 |
| CSPN0376 | CASPER | Calgary | Adult | 3 | GPSC12 | 22.82 | 2,020,856 | 147 | 28,531 | SAMN48917876 |
| CSPN0378 | CASPER | Calgary | Adult | 14 | GPSC18 | 129.03 | 2,063,616 | 94 | 52,673 | SAMN48917878 |
| CSPN0175 | CASPER | Calgary | Pediatric | 6B | GPSC76 | 73.02 | 2,141,698 | 126 | 53,326 | SAMN48917682 |
| CSPN0176 | CASPER | Calgary | Pediatric | 18C | GPSC3 | 390.42 | 2,096,019 | 45 | 150,097 | SAMN48917683 |
| CSPN0177 | CASPER | Calgary | Pediatric | 3 | GPSC7 | 206.27 | 2,089,593 | 63 | 72,074 | SAMN48917684 |
| CSPN0380 | CASPER | Calgary | Adult | 4 | GPSC27 | 345.32 | 2,092,221 | 98 | 49,902 | SAMN48917880 |
| CSPN0178 | CASPER | Calgary | Pediatric | NT ^f^ | GPSCNA | 351.3 | 2,090,072 | 62 | 97,400 | SAMN48917685 |
| CSPN0385 | CASPER | Calgary | Adult | 8 | GPSC98 | 277.33 | 1,989,597 | 43 | 83,074 | SAMN48917885 |
| CSPN0382 | CASPER | Calgary | Adult | 14 | GPSC3 | 187.08 | 2,094,495 | 66 | 61,251 | SAMN48917882 |
| CSPN0381 | CASPER | Calgary | Adult | 3 | GPSC12 | 252.33 | 2,021,601 | 68 | 67,077 | SAMN48917881 |
| CSPN0383 | CASPER | Calgary | Adult | 3 | GPSC12 | 202.21 | 2,019,384 | 59 | 67,276 | SAMN48917883 |
| CSPN0179 | CASPER | Calgary | Pediatric | 15C | GPSC4 | 131.98 | 2,097,067 | 94 | 48,560 | SAMN48917686 |
| CSPN0388 | CASPER | Calgary | Adult | 8 | GPSC98 | 192.46 | 1,988,162 | 47 | 67,403 | SAMN48917888 |
| CSPN0389 | CASPER | Calgary | Adult | 8 | GPSC3 | 162.1 | 2,035,382 | 56 | 76,855 | SAMN48917889 |
| CSPN0180 | CASPER | Calgary | Pediatric | 19F | GPSC119 | 116.24 | 2,103,093 | 107 | 39,630 | SAMN48917687 |
| CSPN0181 | CASPER | Calgary | Pediatric | 8 | GPSC98 | 50.32 | 1,988,433 | 118 | 32,073 | SAMN48917688 |
| CSPN0182 | CASPER | Calgary | Pediatric | 22F | GPSC19 | 121.82 | 2,071,706 | 75 | 59,060 | SAMN48917689 |
| CSPN0391 | CASPER | Calgary | Adult | 8 | GPSC98 | 293.14 | 1,990,374 | 46 | 143,943 | SAMN48917891 |
| CSPN0390 | CASPER | Calgary | Adult | 8 | GPSC98 | 247.3 | 1,991,934 | 43 | 102,085 | SAMN48917890 |
| CSPN0183 | CASPER | Calgary | Pediatric | 38 | GPSC38 | 89.84 | 2,128,333 | 139 | 36,293 | SAMN48917690 |
| CSPN0393 | CASPER | Calgary | Adult | 8 | GPSC98 | 139.04 | 1,986,939 | 103 | 49,924 | SAMN48917893 |
| CSPN0394 | CASPER | Calgary | Adult | 5 | GPSC8 | 246.84 | 2,074,170 | 85 | 57,407 | SAMN48917894 |
| CSPN0186 | CASPER | Calgary | Pediatric | 14 | GPSC39 | 181.83 | 2,100,151 | 87 | 47,101 | SAMN48917693 |
| CSPN0184 | CASPER | Calgary | Pediatric | 6B | GPSC24 | 112.46 | 2,072,514 | 59 | 65,639 | SAMN48917691 |
| CSPN0398 | CASPER | Calgary | Adult | 8 | GPSC98 | 251.81 | 1,989,576 | 50 | 92,016 | SAMN48917898 |
| CSPN0395 | CASPER | Calgary | Adult | 7F | GPSC32 | 308.31 | 1,990,384 | 48 | 80,722 | SAMN48917895 |
| CSPN0397 | CASPER | Calgary | Adult | 3 | GPSC12 | 191.02 | 2,020,966 | 51 | 99,909 | SAMN48917897 |
| CSPN0396 | CASPER | Calgary | Adult | 8 | GPSC3 | 346.48 | 2,034,103 | 45 | 80,958 | SAMN48917896 |
| CSPN0185 | CASPER | Calgary | Pediatric | 19F | GPSC119 | 183.28 | 2,177,672 | 63 | 99,094 | SAMN48917692 |
| CSPN0411 | CASPER | Calgary | Adult | 5 | GPSC8 | 350.82 | 2,076,562 | 77 | 69,868 | SAMN48917911 |
| CSPN0196 | CASPER | Calgary | Pediatric | 19A | GPSC4 | 197.76 | 2,162,582 | 74 | 75,736 | SAMN48917702 |
| CSPN0197 | CASPER | Calgary | Pediatric | 14 | GPSC39 | 102.04 | 2,127,465 | 151 | 53,675 | SAMN48917703 |
| CSPN0399 | CASPER | Calgary | Adult | 8 | GPSC98 | 253.64 | 1,992,796 | 60 | 91,831 | SAMN48917899 |
| CSPN0187 | CASPER | Calgary | Pediatric | 3 | GPSC12 | 188.22 | 2,025,088 | 73 | 71,018 | SAMN48917694 |
| CSPN0188 | CASPER | Calgary | Pediatric | 9V | GPSC6 | 213.66 | 2,114,016 | 84 | 58,990 | SAMN48917695 |
| CSPN0400 | CASPER | Calgary | Adult | 3 | GPSC12 | 342.13 | 2,022,465 | 50 | 88,171 | SAMN48917900 |
| CSPN0190 | CASPER | Calgary | Pediatric | 14 | GPSC39 | 109.24 | 2,068,423 | 100 | 35,095 | SAMN48917696 |
| CSPN0401 | CASPER | Calgary | Adult | 8 | GPSC98 | 270.17 | 1,990,763 | 51 | 101,428 | SAMN48917901 |
| CSPN0402 | CASPER | Calgary | Adult | 8 | GPSC3 | 332.81 | 2,037,049 | 48 | 138,505 | SAMN48917902 |
| CSPN0404 | CASPER | Calgary | Adult | 4 | GPSC27 | 210.4 | 2,127,264 | 101 | 63,795 | SAMN48917904 |
| CSPN0403 | CASPER | Calgary | Adult | 4 | GPSC27 | 392.6 | 2,111,303 | 91 | 49,732 | SAMN48917903 |
| CSPN0191 | CASPER | Calgary | Pediatric | 9V | GPSC6 | 158 | 2,070,553 | 66 | 68,194 | SAMN48917697 |
| CSPN0405 | CASPER | Calgary | Adult | 5 | GPSC8 | 228.48 | 2,073,069 | 78 | 69,926 | SAMN48917905 |
| CSPN0409 | CASPER | Calgary | Adult | 5 | GPSC8 | 197.86 | 2,074,247 | 64 | 69,411 | SAMN48917909 |
| CSPN0406 | CASPER | Calgary | Adult | 5 | GPSC8 | 155.3 | 2,070,444 | 106 | 43,535 | SAMN48917906 |
| CSPN0408 | CASPER | Calgary | Adult | 5 | GPSC8 | 234.8 | 2,072,764 | 67 | 55,034 | SAMN48917908 |
| CSPN0407 | CASPER | Calgary | Adult | 3 | GPSC12 | 556.77 | 2,019,925 | 40 | 89,049 | SAMN48917907 |
| CSPN0192 | CASPER | Calgary | Pediatric | 5 | GPSC8 | 117.94 | 2,079,660 | 76 | 61,688 | SAMN48917698 |
| CSPN0193 | CASPER | Calgary | Pediatric | 5 | GPSC8 | 64.88 | 2,077,392 | 106 | 49,190 | SAMN48917699 |
| CSPN0410 | CASPER | Calgary | Adult | 5 | GPSC8 | 227.07 | 2,074,741 | 71 | 77,974 | SAMN48917910 |
| CSPN0194 | CASPER | Calgary | Pediatric | 33A | GPSC3 | 91.47 | 2,015,468 | 75 | 54,088 | SAMN48917700 |
| CSPN0195 | CASPER | Calgary | Pediatric | 9V | GPSC6 | 54.52 | 2,126,024 | 89 | 54,904 | SAMN48917701 |
| CSPN0415 | CASPER | Calgary | Adult | 8 | GPSC3 | 622.71 | 1,981,277 | 44 | 121,251 | SAMN48917915 |
| CSPN0416 | CASPER | Calgary | Adult | 8 | GPSC98 | 519.28 | 1,986,130 | 63 | 91,869 | SAMN48917916 |
| CSPN0412 | CASPER | Calgary | Adult | 5 | GPSC8 | 33 | 2,076,645 | 115 | 41,801 | SAMN48917912 |
| CSPN0414 | CASPER | Calgary | Adult | 5 | GPSC8 | 21.5 | 2,071,145 | 193 | 20,826 | SAMN48917914 |
| CSPN0413 | CASPER | Calgary | Adult | 5 | GPSC8 | 432.7 | 2,071,979 | 72 | 69,902 | SAMN48917913 |
| CSPN0417 | CASPER | Calgary | Adult | 5 | GPSC8 | 597.8 | 2,074,365 | 69 | 87,040 | SAMN48917917 |
| CSPN0418 | CASPER | Calgary | Adult | 5 | GPSC8 | 479.6 | 2,074,749 | 80 | 64,743 | SAMN48917918 |
| CSPN0420 | CASPER | Calgary | Adult | 5 | GPSC8 | 319.15 | 2,073,087 | 94 | 55,569 | SAMN48917920 |
| CSPN0419 | CASPER | Calgary | Adult | 5 | GPSC8 | 285.46 | 2,073,211 | 70 | 69,918 | SAMN48917919 |
| CSPN0198 | CASPER | Calgary | Pediatric | 22F | GPSC19 | 146.1 | 2,036,890 | 89 | 55,651 | SAMN48917704 |
| CSPN0425 | CASPER | Calgary | Adult | 5 | GPSC8 | 52.34 | 2,075,540 | 97 | 45,160 | SAMN48917925 |
| CSPN0422 | CASPER | Calgary | Adult | 5 | GPSC8 | 48.67 | 2,077,874 | 97 | 45,915 | SAMN48917922 |
| CSPN0427 | CASPER | Calgary | Adult | 5 | GPSC8 | 70.32 | 2,079,179 | 115 | 43,492 | SAMN48917927 |
| CSPN0428 | CASPER | Calgary | Adult | 5 | GPSC8 | 68.16 | 2,078,263 | 85 | 53,353 | SAMN48917928 |
| CSPN0421 | CASPER | Calgary | Adult | 5 | GPSC8 | 45.63 | 2,077,125 | 78 | 53,514 | SAMN48917921 |
| CSPN0424 | CASPER | Calgary | Adult | 5 | GPSC8 | 59.84 | 2,077,421 | 109 | 45,544 | SAMN48917924 |
| CSPN0426 | CASPER | Calgary | Adult | 5 | GPSC8 | 51.05 | 2,077,526 | 104 | 45,995 | SAMN48917926 |
| CSPN0423 | CASPER | Calgary | Adult | 5 | GPSC8 | 49.11 | 2,078,048 | 86 | 45,913 | SAMN48917923 |
| CSPN0199 | CASPER | Calgary | Pediatric | 22F | GPSC19 | 168.49 | 2,036,861 | 41 | 75,117 | SAMN48917705 |
| CSPN0200 | CASPER | Calgary | Pediatric | 3 | GPSC12 | 99.92 | 2,014,624 | 63 | 63,447 | SAMN48917706 |
| CSPN0439 | CASPER | Calgary | Adult | 5 | GPSC8 | 59.66 | 2,076,656 | 136 | 27,472 | SAMN48917939 |
| CSPN0429 | CASPER | Calgary | Adult | 5 | GPSC8 | 63.98 | 2,078,289 | 108 | 32,045 | SAMN48917929 |
| CSPN0432 | CASPER | Calgary | Adult | 5 | GPSC8 | 47.68 | 2,083,851 | 129 | 38,243 | SAMN48917932 |
| CSPN0431 | CASPER | Calgary | Adult | 5 | GPSC8 | 62.53 | 2,077,984 | 151 | 30,698 | SAMN48917931 |
| CSPN0430 | CASPER | Calgary | Adult | 5 | GPSC8 | 53.11 | 2,077,796 | 102 | 48,638 | SAMN48917930 |
| CSPN0435 | CASPER | Calgary | Adult | 5 | GPSC8 | 63.52 | 2,080,080 | 104 | 48,381 | SAMN48917935 |
| CSPN0433 | CASPER | Calgary | Adult | 5 | GPSC8 | 49.08 | 2,078,644 | 75 | 65,049 | SAMN48917933 |
| CSPN0436 | CASPER | Calgary | Adult | 5 | GPSC8 | 58.27 | 2,079,546 | 105 | 44,167 | SAMN48917936 |
| CSPN0434 | CASPER | Calgary | Adult | 4 | GPSC27 | 55.48 | 2,119,692 | 84 | 48,961 | SAMN48917934 |
| CSPN0202 | CASPER | Calgary | Pediatric | 4 | GPSC27 | 196.07 | 2,121,962 | 100 | 48,893 | SAMN48917708 |
| CSPN0201 | CASPER | Calgary | Pediatric | 19A | GPSC4 | 61.56 | 2,112,537 | 136 | 32,906 | SAMN48917707 |
| CSPN0442 | CASPER | Calgary | Adult | 5 | GPSC8 | 57.54 | 2,078,264 | 117 | 42,639 | SAMN48917942 |
| CSPN0437 | CASPER | Calgary | Adult | 5 | GPSC8 | 62.72 | 2,076,553 | 119 | 46,041 | SAMN48917937 |
| CSPN0440 | CASPER | Calgary | Adult | 5 | GPSC8 | 61.79 | 2,079,377 | 99 | 46,481 | SAMN48917940 |
| CSPN0438 | CASPER | Calgary | Adult | 5 | GPSC8 | 62.61 | 2,078,646 | 107 | 50,583 | SAMN48917938 |
| CSPN0441 | CASPER | Calgary | Adult | 5 | GPSC8 | 66.69 | 2,077,339 | 113 | 35,245 | SAMN48917941 |
| CSPN0444 | CASPER | Calgary | Adult | 5 | GPSC8 | 49.38 | 2,078,545 | 64 | 71,390 | SAMN48917944 |
| CSPN0443 | CASPER | Calgary | Adult | 5 | GPSC8 | 66.83 | 2,077,661 | 135 | 38,293 | SAMN48917943 |
| CSPN0446 | CASPER | Calgary | Adult | 5 | GPSC8 | 50.07 | 2,079,449 | 78 | 53,499 | SAMN48917946 |
| CSPN0448 | CASPER | Calgary | Adult | 5 | GPSC8 | 50.21 | 2,075,726 | 84 | 55,986 | SAMN48917948 |
| CSPN0445 | CASPER | Calgary | Adult | 5 | GPSC8 | 49.04 | 2,081,601 | 70 | 68,976 | SAMN48917945 |
| CSPN0447 | CASPER | Calgary | Adult | 4 | GPSC27 | 51.68 | 2,127,700 | 90 | 54,504 | SAMN48917947 |
| CSPN0450 | CASPER | Calgary | Adult | 5 | GPSC8 | 58.87 | 2,079,786 | 116 | 45,902 | SAMN48917950 |
| CSPN0449 | CASPER | Calgary | Adult | 5 | GPSC8 | 66.99 | 2,079,498 | 101 | 46,205 | SAMN48917949 |
| CSPN0451 | CASPER | Calgary | Adult | 5 | GPSC8 | 62.38 | 2,076,100 | 93 | 44,015 | SAMN48917951 |
| CSPN0205 | CASPER | Calgary | Pediatric | 4 | GPSC27 | 68.52 | 2,120,505 | 131 | 35,089 | SAMN48917711 |
| CSPN0455 | CASPER | Calgary | Adult | 5 | GPSC8 | 61.87 | 2,078,795 | 99 | 44,116 | SAMN48917955 |
| CSPN0454 | CASPER | Calgary | Adult | 5 | GPSC8 | 67.21 | 2,075,784 | 126 | 41,828 | SAMN48917954 |
| CSPN0456 | CASPER | Calgary | Adult | 5 | GPSC8 | 59.41 | 2,077,183 | 83 | 54,044 | SAMN48917956 |
| CSPN0453 | CASPER | Calgary | Adult | 7F | GPSC15 | 70.64 | 1,994,424 | 81 | 61,815 | SAMN48917953 |
| CSPN0457 | CASPER | Calgary | Adult | 3 | GPSC12 | 43.26 | 2,025,442 | 44 | 93,364 | SAMN48917957 |
| CSPN0452 | CASPER | Calgary | Adult | 19A | GPSC4 | 66.89 | 2,100,852 | 70 | 63,107 | SAMN48917952 |
| CSPN0204 | CASPER | Calgary | Pediatric | 5 | GPSC8 | 39.34 | 2,077,236 | 124 | 27,628 | SAMN48917710 |
| CSPN0203 | CASPER | Calgary | Pediatric | 23B | GPSC5 | 83.41 | 2,170,566 | 52 | 83,543 | SAMN48917709 |
| CSPN0206 | CASPER | Calgary | Pediatric | 22F | GPSC19 | 114.99 | 2,038,921 | 78 | 48,983 | SAMN48917712 |
| CSPN0458 | CASPER | Calgary | Adult | 8 | GPSC98 | 46.03 | 1,993,689 | 68 | 57,650 | SAMN48917958 |
| CSPN0459 | CASPER | Calgary | Adult | 5 | GPSC8 | 57.34 | 2,077,791 | 78 | 53,654 | SAMN48917959 |
| CSPN0208 | CASPER | Calgary | Pediatric | 17F | GPSC49 | 98.44 | 2,084,741 | 69 | 48,554 | SAMN48917714 |
| CSPN0209 | CASPER | Calgary | Pediatric | 23B | GPSC5 | 142.46 | 2,170,477 | 77 | 79,862 | SAMN48917715 |
| CSPN0207 | CASPER | Calgary | Pediatric | 3 | GPSC12 | 70.11 | 2,023,294 | 76 | 61,889 | SAMN48917713 |
| CSPN0210 | CASPER | Calgary | Pediatric | 19A | GPSC10 | 111.04 | 2,096,144 | 87 | 59,108 | SAMN48917716 |
| CSPN0460 | CASPER | Calgary | Adult | 3 | GPSC83 | 53.65 | 1,995,033 | 71 | 54,706 | SAMN48917960 |
| CSPN0211 | CASPER | Calgary | Pediatric | 7F | GPSC15 | 97.15 | 1,994,270 | 90 | 48,351 | SAMN48917717 |
| CSPN0222 | CASPER | Calgary | Pediatric | 35F | GPSC323 | 170.32 | 2,082,304 | 104 | 58,126 | SAMN48917727 |
| CSPN0461 | CASPER | Calgary | Adult | 8 | GPSC98 | 46.44 | 1,990,757 | 60 | 50,018 | SAMN48917961 |
| CSPN0462 | CASPER | Calgary | Adult | 3 | GPSC12 | 54.88 | 2,024,677 | 81 | 44,628 | SAMN48917962 |
| CSPN0212 | CASPER | Calgary | Pediatric | 6A | GPSC13 | 88.01 | 2,084,530 | 79 | 55,093 | SAMN48917718 |
| CSPN0213 | CASPER | Calgary | Pediatric | 29 | GPSC59 | 61.26 | 2,008,095 | 88 | 47,796 | SAMN48917719 |
| CSPN0463 | CASPER | Calgary | Adult | 5 | GPSC8 | 56.37 | 2,077,852 | 118 | 43,029 | SAMN48917963 |
| CSPN0464 | CASPER | Calgary | Adult | 5 | GPSC8 | 53.47 | 2,075,875 | 82 | 57,866 | SAMN48917964 |
| CSPN0214 | CASPER | Calgary | Pediatric | 18C | GPSC3 | 98.45 | 2,061,652 | 54 | 81,835 | SAMN48917720 |
| CSPN0216 | CASPER | Calgary | Pediatric | 23B | GPSC7 | 195.86 | 2,145,577 | 77 | 72,429 | SAMN48917721 |
| CSPN0467 | CASPER | Calgary | Adult | 8 | GPSC224 | 60.66 | 2,010,799 | 86 | 48,862 | SAMN48917967 |
| CSPN0465 | CASPER | Calgary | Adult | 5 | GPSC8 | 60.86 | 2,076,739 | 130 | 33,534 | SAMN48917965 |
| CSPN0466 | CASPER | Calgary | Adult | 5 | GPSC8 | 51.07 | 2,078,932 | 120 | 41,828 | SAMN48917966 |
| CSPN0217 | CASPER | Calgary | Pediatric | 19A | GPSC1 | 58.34 | 2,055,537 | 116 | 35,141 | SAMN48917722 |
| CSPN0468 | CASPER | Calgary | Adult | 19A | GPSC9 | 50.32 | 2,064,884 | 77 | 74,034 | SAMN48917968 |
| CSPN0469 | CASPER | Calgary | Adult | 9V | GPSC43 | 36.36 | 2,083,401 | 62 | 70,986 | SAMN48917969 |
| CSPN0218 | CASPER | Calgary | Pediatric | 11A | GPSC3 | 67.7 | 2,021,647 | 90 | 57,642 | SAMN48917723 |
| CSPN0219 | CASPER | Calgary | Pediatric | 3 | GPSC12 | 63.47 | 2,061,681 | 83 | 55,832 | SAMN48917724 |
| CSPN0471 | CASPER | Calgary | Adult | 7F | GPSC15 | 48.91 | 1,997,339 | 88 | 53,549 | SAMN48917971 |
| CSPN0470 | CASPER | Calgary | Adult | 9V | GPSC6 | 52.71 | 2,104,360 | 73 | 80,319 | SAMN48917970 |
| CSPN0221 | CASPER | Calgary | Pediatric | 19A | GPSC1 | 77.56 | 2,036,618 | 72 | 58,377 | SAMN48917726 |
| CSPN0220 | CASPER | Calgary | Pediatric | 19A | GPSC9 | 78.36 | 2,067,844 | 72 | 68,476 | SAMN48917725 |
| CSPN0472 | CASPER | Calgary | Adult | 7F | GPSC15 | 61.38 | 1,992,385 | 85 | 57,953 | SAMN48917972 |
| CSPN0223 | CASPER | Calgary | Pediatric | 19A | GPSC1 | 64.02 | 2,054,866 | 106 | 42,760 | SAMN48917728 |
| CSPN0473 | CASPER | Calgary | Adult | 14 | GPSC18 | 46.98 | 2,066,805 | 121 | 35,704 | SAMN48917973 |
| CSPN0475 | CASPER | Calgary | Adult | 3 | GPSC12 | 52.75 | 2,019,929 | 183 | 27,938 | SAMN48917975 |
| CSPN0474 | CASPER | Calgary | Adult | 7F | GPSC15 | 52.66 | 1,996,793 | 119 | 29,657 | SAMN48917974 |
| CSPN0224 | CASPER | Calgary | Pediatric | 7F | GPSC15 | 107.76 | 1,995,604 | 85 | 60,177 | SAMN48917729 |
| CSPN0476 | CASPER | Calgary | Adult | 5 | GPSC8 | 49.14 | 2,079,352 | 79 | 47,558 | SAMN48917976 |
| CSPN0477 | CASPER | Calgary | Adult | 19A | GPSC9 | 55.69 | 2,046,408 | 97 | 54,224 | SAMN48917977 |
| CSPN0225 | CASPER | Calgary | Pediatric | 19A | GPSC9 | 88.06 | 2,070,387 | 67 | 58,093 | SAMN48917730 |
| CSPN0226 | CASPER | Calgary | Pediatric | 19A | GPSC4 | 82.35 | 2,097,999 | 96 | 42,125 | SAMN48917731 |
| CSPN0228 | CASPER | Calgary | Pediatric | 19A | GPSC9 | 91.52 | 2,058,917 | 72 | 66,347 | SAMN48917733 |
| CSPN0229 | CASPER | Calgary | Pediatric | 19A | GPSC9 | 70.17 | 2,067,440 | 135 | 35,205 | SAMN48917734 |
| CSPN0227 | CASPER | Calgary | Pediatric | 19A | GPSC109 | 102.48 | 2,129,750 | 89 | 49,954 | SAMN48917732 |
| CSPN0479 | CASPER | Calgary | Adult | 3 | GPSC363 | 49.63 | 1,989,895 | 125 | 32,915 | SAMN48917979 |
| CSPN0478 | CASPER | Calgary | Adult | 7F | GPSC15 | 52.41 | 1,992,893 | 134 | 33,309 | SAMN48917978 |
| CSPN0480 | CASPER | Calgary | Adult | 19A | GPSC4 | 57.14 | 2,101,750 | 138 | 44,314 | SAMN48917980 |
| CSPN0230 | CASPER | Calgary | Pediatric | 19A | GPSC27 | 39.8 | 2,139,231 | 159 | 29,162 | SAMN48917735 |
| CSPN0481 | CASPER | Calgary | Adult | 3 | GPSC12 | 44.74 | 2,019,215 | 109 | 53,854 | SAMN48917981 |
| CSPN0484 | CASPER | Calgary | Adult | 3 | GPSC12 | 52.73 | 2,023,564 | 66 | 66,123 | SAMN48917984 |
| CSPN0231 | CASPER | Calgary | Pediatric | 3 | GPSC12 | 49.89 | 2,010,576 | 83 | 58,464 | SAMN48917736 |
| CSPN0482 | CASPER | Calgary | Adult | 7F | GPSC15 | 48.34 | 1,998,100 | 72 | 58,021 | SAMN48917982 |
| CSPN0483 | CASPER | Calgary | Adult | 19A | GPSC3 | 56.97 | 2,077,110 | 50 | 95,283 | SAMN48917983 |
| CSPN0486 | CASPER | Calgary | Adult | 3 | GPSC12 | 50.13 | 2,022,037 | 82 | 45,574 | SAMN48917986 |
| CSPN0487 | CASPER | Calgary | Adult | 8 | GPSC98 | 49.35 | 1,992,017 | 71 | 56,272 | SAMN48917987 |
| CSPN0583 | CASPER | Calgary | Pediatric | 3 | GPSC12 | 63.34 | 1,989,491 | 45 | 91,444 | SAMN48918082 |
| CSPN0485 | CASPER | Calgary | Adult | 3 | GPSC12 | 42.21 | 2,026,966 | 51 | 139,875 | SAMN48917985 |
| CSPN0232 | CASPER | Calgary | Pediatric | 19A | GPSC4 | 80.52 | 2,065,656 | 61 | 72,396 | SAMN48917737 |
| CSPN0233 | CASPER | Calgary | Pediatric | 19A | GPSC4 | 143.31 | 2,112,738 | 65 | 75,013 | SAMN48917738 |
| CSPN0235 | CASPER | Calgary | Pediatric | 19A | GPSC1 | 50.62 | 2,054,370 | 97 | 56,739 | SAMN48917740 |
| CSPN0236 | CASPER | Calgary | Pediatric | 8 | GPSC3 | 72.27 | 1,999,078 | 55 | 77,658 | SAMN48917741 |
| CSPN0234 | CASPER | Calgary | Pediatric | 33F | GPSC3 | 55.82 | 2,068,217 | 62 | 69,989 | SAMN48917739 |
| CSPN0237 | CASPER | Calgary | Pediatric | 5 | GPSC8 | 159.08 | 2,078,026 | 112 | 45,902 | SAMN48917742 |
| CSPN0238 | CASPER | Calgary | Pediatric | 38 | GPSC38 | 187.42 | 2,119,512 | 125 | 41,356 | SAMN48917743 |
| CSPN0488 | CASPER | Calgary | Adult | 8 | GPSC98 | 56.22 | 1,992,820 | 55 | 69,966 | SAMN48917988 |
| CSPN0574 | CASPER | Calgary | Pediatric | 19A | GPSC99 | 52.31 | 2,123,922 | 138 | 37,047 | SAMN48918074 |
| CSPN0575 | CASPER | Calgary | Pediatric | 10A | GPSC36 | 61.49 | 2,062,856 | 68 | 55,345 | SAMN48918075 |
| CSPN0576 | CASPER | Calgary | Pediatric | 19A | GPSC4 | 56.2 | 2,061,553 | 105 | 43,364 | SAMN48918076 |
| CSPN0577 | CASPER | Calgary | Pediatric | 23B | GPSC7 | 48.61 | 2,134,450 | 126 | 38,726 | SAMN48918077 |
| CSPN0489 | CASPER | Calgary | Adult | 8 | GPSC3 | 61.79 | 2,022,765 | 57 | 64,936 | SAMN48917989 |
| CSPN0490 | CASPER | Calgary | Adult | 7F | GPSC15 | 56.33 | 1,999,206 | 88 | 50,586 | SAMN48917990 |
| CSPN0579 | CASPER | Calgary | Pediatric | 6A | GPSC29 | 63.29 | 2,096,969 | 64 | 83,368 | SAMN48918079 |
| CSPN0578 | CASPER | Calgary | Pediatric | 22F | GPSC19 | 62.94 | 2,076,785 | 77 | 58,190 | SAMN48918078 |
| CSPN0491 | CASPER | Calgary | Adult | 19A | GPSC4 | 54.42 | 2,069,237 | 95 | 55,172 | SAMN48917991 |
| CSPN0581 | CASPER | Calgary | Pediatric | 3 | GPSC12 | 48.51 | 1,988,731 | 45 | 141,650 | SAMN48918080 |
| CSPN0582 | CASPER | Calgary | Pediatric | 19A | GPSC4 | 48.93 | 2,068,632 | 76 | 60,445 | SAMN48918081 |
| CSPN0496 | CASPER | Calgary | Adult | 19A | GPSC9 | 44.97 | 2,067,843 | 53 | 73,342 | SAMN48917996 |
| CSPN0492 | CASPER | Calgary | Adult | 8 | GPSC98 | 53.37 | 1,992,944 | 80 | 45,407 | SAMN48917992 |
| CSPN0584 | CASPER | Calgary | Pediatric | 12F | GPSC32 | 54.12 | 1,991,060 | 66 | 50,012 | SAMN48918083 |
| CSPN0493 | CASPER | Calgary | Adult | 8 | GPSC336 | 45.61 | 1,969,422 | 151 | 24,693 | SAMN48917993 |
| CSPN0494 | CASPER | Calgary | Adult | 19A | GPSC4 | 61.12 | 2,106,192 | 130 | 37,465 | SAMN48917994 |
| CSPN0585 | CASPER | Calgary | Pediatric | 7F | GPSC15 | 51.39 | 1,999,304 | 99 | 46,401 | SAMN48918084 |
| CSPN0586 | CASPER | Calgary | Pediatric | 23F | GPSC94 | 55.57 | 2,147,496 | 105 | 50,094 | SAMN48918085 |
| CSPN0495 | CASPER | Calgary | Adult | 7F | GPSC15 | 53.45 | 1,998,932 | 97 | 36,420 | SAMN48917995 |
| CSPN0587 | CASPER | Calgary | Pediatric | 35B | GPSC75 | 50.66 | 2,074,918 | 67 | 60,778 | SAMN48918086 |
| CSPN0588 | CASPER | Calgary | Pediatric | 7F | GPSC15 | 51.19 | 1,992,006 | 62 | 67,185 | SAMN48918087 |
| CSPN0589 | CASPER | Calgary | Pediatric | 19A | GPSC4 | 51.92 | 2,142,211 | 110 | 40,632 | SAMN48918088 |
| CSPN0497 | CASPER | Calgary | Adult | 7F | GPSC15 | 43.7 | 2,000,926 | 94 | 47,699 | SAMN48917997 |
| CSPN0590 | CASPER | Calgary | Pediatric | 19A | GPSC27 | 47.53 | 2,214,095 | 162 | 28,126 | SAMN48918089 |
| CSPN0591 | CASPER | Calgary | Pediatric | 3 | GPSC12 | 69.62 | 2,013,872 | 117 | 34,467 | SAMN48918090 |
| CSPN0592 | CASPER | Calgary | Pediatric | 19A | GPSC27 | 47.73 | 2,149,980 | 84 | 53,880 | SAMN48918091 |
| CSPN0498 | CASPER | Calgary | Adult | 4 | GPSC27 | 54.22 | 2,119,586 | 85 | 55,222 | SAMN48917998 |
| CSPN0593 | CASPER | Calgary | Pediatric | 7F | GPSC15 | 40.74 | 1,997,766 | 80 | 47,368 | SAMN48918092 |
| CSPN0499 | CASPER | Calgary | Adult | 4 | GPSC27 | 65.24 | 2,121,811 | 131 | 34,125 | SAMN48917999 |
| CSPN0594 | CASPER | Calgary | Pediatric | 15C | GPSC48 | 60.02 | 2,087,658 | 90 | 50,989 | SAMN48918093 |
| CSPN0595 | CASPER | Calgary | Pediatric | 15A | GPSC9 | 49.63 | 2,088,851 | 76 | 54,718 | SAMN48918094 |
| CSPN0500 | CASPER | Calgary | Adult | 4 | GPSC27 | 51.11 | 2,111,488 | 117 | 33,574 | SAMN48918000 |
| CSPN0503 | CASPER | Calgary | Adult | 8 | GPSC98 | 58.29 | 1,992,952 | 82 | 44,976 | SAMN48918003 |
| CSPN0502 | CASPER | Calgary | Adult | 7F | GPSC15 | 58.03 | 1,996,230 | 123 | 29,600 | SAMN48918002 |
| CSPN0501 | CASPER | Calgary | Adult | 8 | GPSC98 | 52.57 | 1,992,261 | 100 | 44,966 | SAMN48918001 |
| CSPN0505 | CASPER | Calgary | Adult | 3 | GPSC12 | 53.12 | 2,046,319 | 74 | 63,270 | SAMN48918005 |
| CSPN0504 | CASPER | Calgary | Adult | 7F | GPSC15 | 63.6 | 1,995,622 | 138 | 26,926 | SAMN48918004 |
| CSPN0596 | CASPER | Calgary | Pediatric | 7F | GPSC15 | 46 | 1,999,888 | 92 | 43,674 | SAMN48918095 |
| CSPN0604 | CASPER | Calgary | Pediatric | 19A | GPSC27 | 51.38 | 2,179,610 | 131 | 34,676 | SAMN48918103 |
| CSPN0607 | CASPER | Calgary | Pediatric | 10A | GPSC36 | 54.27 | 2,065,817 | 58 | 65,504 | SAMN48918106 |
| CSPN0597 | CASPER | Calgary | Pediatric | 19A | GPSC1 | 51.87 | 2,048,646 | 88 | 50,955 | SAMN48918096 |
| CSPN0506 | CASPER | Calgary | Adult | 19A | GPSC9 | 44.87 | 2,067,417 | 63 | 77,666 | SAMN48918006 |
| CSPN0598 | CASPER | Calgary | Pediatric | 33F | GPSC3 | 49.07 | 2,048,942 | 109 | 44,968 | SAMN48918097 |
| CSPN0599 | CASPER | Calgary | Pediatric | 3 | GPSC12 | 42.89 | 2,022,477 | 92 | 41,814 | SAMN48918098 |
| CSPN0507 | CASPER | Calgary | Adult | 3 | GPSC12 | 50.2 | 2,020,163 | 121 | 43,564 | SAMN48918007 |
| CSPN0508 | CASPER | Calgary | Adult | 19A | GPSC1 | 55.93 | 2,058,891 | 85 | 57,768 | SAMN48918008 |
| CSPN0509 | CASPER | Calgary | Adult | 19A | GPSC27 | 50.89 | 2,107,217 | 88 | 57,156 | SAMN48918009 |
| CSPN0510 | CASPER | Calgary | Adult | 19A | GPSC99 | 50.25 | 2,179,087 | 148 | 33,312 | SAMN48918010 |
| CSPN0511 | CASPER | Calgary | Adult | 4 | GPSC27 | 369.17 | 2,106,641 | 76 | 50,819 | SAMN48918011 |
| CSPN0600 | CASPER | Calgary | Pediatric | 11A | GPSC3 | 44.55 | 2,021,061 | 74 | 54,441 | SAMN48918099 |
| CSPN0601 | CASPER | Calgary | Pediatric | 11A | GPSC3 | 59.15 | 2,009,909 | 57 | 69,873 | SAMN48918100 |
| CSPN0602 | CASPER | Calgary | Pediatric | 7F | GPSC15 | 47.07 | 1,997,361 | 130 | 34,832 | SAMN48918101 |
| CSPN0512 | CASPER | Calgary | Adult | 3 | GPSC12 | 44.32 | 2,043,087 | 107 | 51,249 | SAMN48918012 |
| CSPN0603 | CASPER | Calgary | Pediatric | 23B | GPSC7 | 50.42 | 2,072,410 | 92 | 45,767 | SAMN48918102 |
| CSPN0605 | CASPER | Calgary | Pediatric | 19F | GPSC1 | 43.06 | 2,073,056 | 97 | 51,181 | SAMN48918104 |
| CSPN0515 | CASPER | Calgary | Adult | 19A | GPSC27 | 59.97 | 2,143,892 | 132 | 34,126 | SAMN48918015 |
| CSPN0513 | CASPER | Calgary | Adult | 3 | GPSC12 | 62.29 | 1,990,765 | 76 | 53,716 | SAMN48918013 |
| CSPN0514 | CASPER | Calgary | Adult | 7F | GPSC15 | 52.06 | 1,992,897 | 140 | 31,625 | SAMN48918014 |
| CSPN0606 | CASPER | Calgary | Pediatric | 22F | GPSC19 | 44.5 | 2,072,958 | 81 | 47,768 | SAMN48918105 |
| CSPN0608 | CASPER | Calgary | Pediatric | 19A | GPSC4 | 52.22 | 2,124,402 | 84 | 50,763 | SAMN48918107 |
| CSPN0517 | CASPER | Calgary | Adult | 8 | GPSC98 | 59.95 | 1,992,047 | 69 | 64,902 | SAMN48918017 |
| CSPN0516 | CASPER | Calgary | Adult | 3 | GPSC12 | 48.97 | 1,987,705 | 165 | 26,731 | SAMN48918016 |
| CSPN0609 | CASPER | Calgary | Pediatric | 19A | GPSC99 | 63.74 | 2,188,133 | 93 | 51,526 | SAMN48918108 |
| CSPN0610 | CASPER | Calgary | Pediatric | 19A | GPSC5 | 53.71 | 2,192,421 | 133 | 39,529 | SAMN48918109 |
| CSPN0518 | CASPER | Calgary | Adult | 8 | GPSC98 | 54.8 | 1,987,993 | 76 | 67,602 | SAMN48918018 |
| CSPN0611 | CASPER | Calgary | Pediatric | 22F | GPSC19 | 41.74 | 2,057,948 | 128 | 32,480 | SAMN48918110 |
| CSPN0519 | CASPER | Calgary | Adult | 8 | GPSC98 | 54.26 | 1,986,707 | 69 | 55,901 | SAMN48918019 |
| CSPN0520 | CASPER | Calgary | Adult | 3 | GPSC12 | 57.73 | 1,982,059 | 60 | 57,696 | SAMN48918020 |
| CSPN0521 | CASPER | Calgary | Adult | 4 | GPSC27 | 40.98 | 2,105,609 | 131 | 36,614 | SAMN48918021 |
| CSPN0612 | CASPER | Calgary | Pediatric | 23B | GPSC5 | 65.43 | 2,143,060 | 59 | 97,168 | SAMN48918111 |
| CSPN0613 | CASPER | Calgary | Pediatric | 3 | GPSC12 | 54.66 | 1,990,144 | 51 | 77,802 | SAMN48918112 |
| CSPN0251 | CASPER | Calgary | Pediatric | 3 | GPSC12 | 48.13 | 1,986,739 | 119 | 38,696 | SAMN48917756 |
| CSPN0243 | CASPER | Calgary | Pediatric | 19A | GPSC27 | 45.13 | 2,101,010 | 108 | 38,993 | SAMN48917748 |
| CSPN0522 | CASPER | Calgary | Adult | 19A | GPSC9 | 51.05 | 2,083,408 | 80 | 73,401 | SAMN48918022 |
| CSPN0239 | CASPER | Calgary | Pediatric | 19F | GPSC1 | 131.98 | 2,073,651 | 69 | 57,296 | SAMN48917744 |
| CSPN0241 | CASPER | Calgary | Pediatric | 15B | GPSC6 | 43.43 | 2,145,210 | 156 | 36,027 | SAMN48917746 |
| CSPN0242 | CASPER | Calgary | Pediatric | 7F | GPSC15 | 51.75 | 1,993,021 | 105 | 46,518 | SAMN48917747 |
| CSPN0240 | CASPER | Calgary | Pediatric | 8 | GPSC98 | 76 | 1,990,524 | 104 | 44,783 | SAMN48917745 |
| CSPN0523 | CASPER | Calgary | Adult | 4 | GPSC27 | 56.97 | 2,109,192 | 114 | 39,069 | SAMN48918023 |
| CSPN0525 | CASPER | Calgary | Adult | 3 | GPSC12 | 53.06 | 1,989,882 | 81 | 53,502 | SAMN48918025 |
| CSPN0524 | CASPER | Calgary | Adult | 3 | GPSC12 | 55.13 | 1,970,666 | 84 | 47,726 | SAMN48918024 |
| CSPN0244 | CASPER | Calgary | Pediatric | 15C | GPSC4 | 59.89 | 2,122,654 | 101 | 48,797 | SAMN48917749 |
| CSPN0526 | CASPER | Calgary | Adult | 8 | GPSC98 | 45.35 | 1,990,168 | 94 | 60,514 | SAMN48918026 |
| CSPN0527 | CASPER | Calgary | Adult | 4 | GPSC27 | 56.06 | 2,117,575 | 120 | 36,471 | SAMN48918027 |
| CSPN0246 | CASPER | Calgary | Pediatric | 22F | GPSC19 | 49.81 | 2,074,505 | 95 | 47,128 | SAMN48917751 |
| CSPN0245 | CASPER | Calgary | Pediatric | 19A | GPSC9 | 68.77 | 2,066,765 | 69 | 74,504 | SAMN48917750 |
| CSPN0247 | CASPER | Calgary | Pediatric | 3 | GPSC12 | 81.78 | 2,011,193 | 52 | 81,548 | SAMN48917752 |
| CSPN0528 | CASPER | Calgary | Adult | 3 | GPSC12 | 50.65 | 2,020,714 | 84 | 42,165 | SAMN48918028 |
| CSPN0248 | CASPER | Calgary | Pediatric | 6C | GPSC29 | 176.89 | 2,098,961 | 72 | 67,458 | SAMN48917753 |
| CSPN0529 | CASPER | Calgary | Adult | 8 | GPSC98 | 59.95 | 1,992,799 | 59 | 70,892 | SAMN48918029 |
| CSPN0530 | CASPER | Calgary | Adult | 19A | GPSC4 | 57.95 | 2,072,238 | 110 | 36,358 | SAMN48918030 |
| CSPN0249 | CASPER | Calgary | Pediatric | 15B | GPSC6 | 66.47 | 2,135,194 | 142 | 33,272 | SAMN48917754 |
| CSPN0531 | CASPER | Calgary | Adult | 3 | GPSC12 | 63.44 | 2,037,701 | 77 | 62,234 | SAMN48918031 |
| CSPN0250 | CASPER | Calgary | Pediatric | 22F | GPSC19 | 220.3 | 2,111,037 | 73 | 72,502 | SAMN48917755 |
| CSPN0252 | CASPER | Calgary | Pediatric | 23B | GPSC11 | 96.92 | 2,130,686 | 104 | 59,139 | SAMN48917757 |
| CSPN0532 | CASPER | Calgary | Adult | 3 | GPSC12 | 51.26 | 1,982,470 | 56 | 93,967 | SAMN48918032 |
| CSPN0253 | CASPER | Calgary | Pediatric | 15C | GPSC48 | 44.95 | 2,154,435 | 106 | 56,084 | SAMN48917758 |
| CSPN0533 | CASPER | Calgary | Adult | 4 | GPSC27 | 50.64 | 2,111,509 | 129 | 34,572 | SAMN48918033 |
| CSPN0254 | CASPER | Calgary | Pediatric | 6C | GPSC89 | 59.14 | 2,114,606 | 110 | 50,305 | SAMN48917759 |
| CSPN0534 | CASPER | Calgary | Adult | 3 | GPSC12 | 50.43 | 2,020,611 | 91 | 48,810 | SAMN48918034 |
| CSPN0258 | CASPER | Calgary | Pediatric | 11A | GPSC3 | 52.47 | 2,008,840 | 56 | 91,337 | SAMN48917763 |
| CSPN0270 | CASPER | Calgary | Pediatric | 15A | GPSC9 | 58.59 | 2,111,208 | 77 | 60,655 | SAMN48917775 |
| CSPN0255 | CASPER | Calgary | Pediatric | 34 | GPSC45 | 110.18 | 2,061,494 | 47 | 94,622 | SAMN48917760 |
| CSPN0256 | CASPER | Calgary | Pediatric | 22F | GPSC19 | 99.81 | 2,073,203 | 73 | 58,011 | SAMN48917761 |
| CSPN0257 | CASPER | Calgary | Pediatric | 33F | GPSC3 | 55.2 | 2,067,589 | 45 | 91,687 | SAMN48917762 |
| CSPN0260 | CASPER | Calgary | Pediatric | 35F | GPSC36 | 63.35 | 2,058,283 | 64 | 60,173 | SAMN48917765 |
| CSPN0259 | CASPER | Calgary | Pediatric | 33F | GPSC3 | 79.86 | 2,049,740 | 80 | 68,535 | SAMN48917764 |
| CSPN0535 | CASPER | Calgary | Adult | 8 | GPSC98 | 60.07 | 1,994,138 | 72 | 55,284 | SAMN48918035 |
| CSPN0261 | CASPER | Calgary | Pediatric | 21 | GPSC99 | 76.05 | 2,131,119 | 72 | 58,651 | SAMN48917766 |
| CSPN0264 | CASPER | Calgary | Pediatric | 21 | GPSC11 | 56.01 | 2,120,561 | 105 | 45,636 | SAMN48917769 |
| CSPN0263 | CASPER | Calgary | Pediatric | 7C | GPSC698 | 68.27 | 2,073,431 | 75 | 48,697 | SAMN48917768 |
| CSPN0262 | CASPER | Calgary | Pediatric | 29 | GPSC75 | 51.99 | 2,105,209 | 88 | 49,244 | SAMN48917767 |
| CSPN0536 | CASPER | Calgary | Adult | 19A | GPSC1 | 68.51 | 2,025,191 | 77 | 49,553 | SAMN48918036 |
| CSPN0265 | CASPER | Calgary | Pediatric | 22F | GPSC19 | 40.67 | 2,069,533 | 170 | 26,754 | SAMN48917770 |
| CSPN0537 | CASPER | Calgary | Adult | 4 | GPSC27 | 49.73 | 2,107,897 | 87 | 45,887 | SAMN48918037 |
| CSPN0538 | CASPER | Calgary | Adult | 4 | GPSC27 | 59.16 | 2,114,604 | 128 | 30,536 | SAMN48918038 |
| CSPN0539 | CASPER | Calgary | Adult | 8 | GPSC98 | 42.59 | 1,989,347 | 134 | 33,356 | SAMN48918039 |
| CSPN0267 | CASPER | Calgary | Pediatric | 23B | GPSC5 | 57.61 | 2,140,089 | 110 | 50,730 | SAMN48917772 |
| CSPN0268 | CASPER | Calgary | Pediatric | 35F | GPSC75 | 35.62 | 2,140,780 | 94 | 48,707 | SAMN48917773 |
| CSPN0266 | CASPER | Calgary | Pediatric | 23B | GPSC5 | 64.31 | 2,097,049 | 151 | 29,484 | SAMN48917771 |
| CSPN0541 | CASPER | Calgary | Adult | 4 | GPSC27 | 51.03 | 2,101,364 | 112 | 38,946 | SAMN48918041 |
| CSPN0540 | CASPER | Calgary | Adult | 19A | GPSC4 | 52.13 | 2,069,918 | 103 | 43,010 | SAMN48918040 |
| CSPN0269 | CASPER | Calgary | Pediatric | 15A | GPSC9 | 40.62 | 2,076,023 | 80 | 57,136 | SAMN48917774 |
| CSPN0542 | CASPER | Calgary | Adult | 4 | GPSC27 | 55.97 | 2,130,757 | 85 | 58,982 | SAMN48918042 |
| CSPN0543 | CASPER | Calgary | Adult | 4 | GPSC27 | 50.49 | 2,127,121 | 75 | 71,034 | SAMN48918043 |
| CSPN0544 | CASPER | Calgary | Adult | 3 | GPSC12 | 59 | 1,990,077 | 38 | 113,408 | SAMN48918044 |
| CSPN0271 | CASPER | Calgary | Pediatric | 22F | GPSC19 | 48.91 | 2,076,169 | 108 | 46,155 | SAMN48917776 |
| CSPN0275 | CASPER | Calgary | Pediatric | 3 | GPSC12 | 212.04 | 1,975,854 | 62 | 94,383 | SAMN48917780 |
| CSPN0547 | CASPER | Calgary | Adult | 8 | GPSC3 | 59.77 | 2,045,702 | 36 | 120,065 | SAMN48918047 |
| CSPN0282 | CASPER | Calgary | Pediatric | 23B | GPSC11 | 30.07 | 2,126,230 | 85 | 59,369 | SAMN48917787 |
| CSPN0552 | CASPER | Calgary | Adult | 8 | GPSC98 | 57.19 | 1,992,426 | 36 | 137,876 | SAMN48918052 |
| CSPN0286 | CASPER | Calgary | Pediatric | 24A | GPSC6 | 151.11 | 2,095,712 | 53 | 89,829 | SAMN48917791 |
| CSPN0272 | CASPER | Calgary | Pediatric | 33F | GPSC3 | 54.45 | 2,032,459 | 81 | 60,295 | SAMN48917777 |
| CSPN0273 | CASPER | Calgary | Pediatric | 9N | GPSC124 | 66.89 | 2,078,198 | 70 | 60,804 | SAMN48917778 |
| CSPN0546 | CASPER | Calgary | Adult | 3 | GPSC12 | 56.21 | 2,021,963 | 40 | 123,012 | SAMN48918046 |
| CSPN0545 | CASPER | Calgary | Adult | 3 | GPSC12 | 58.77 | 2,013,815 | 36 | 91,082 | SAMN48918045 |
| CSPN0274 | CASPER | Calgary | Pediatric | 34 | GPSC45 | 68.38 | 2,107,255 | 62 | 71,790 | SAMN48917779 |
| CSPN0276 | CASPER | Calgary | Pediatric | 3 | GPSC12 | 72.75 | 2,014,587 | 60 | 88,658 | SAMN48917781 |
| CSPN0277 | CASPER | Calgary | Pediatric | 33F | GPSC3 | 33.46 | 2,051,798 | 110 | 45,691 | SAMN48917782 |
| CSPN0278 | CASPER | Calgary | Pediatric | 19F | GPSC98 | 33.73 | 1,992,361 | 53 | 64,900 | SAMN48917783 |
| CSPN0548 | CASPER | Calgary | Adult | 7F | GPSC15 | 56.48 | 1,996,452 | 76 | 59,264 | SAMN48918048 |
| CSPN0280 | CASPER | Calgary | Pediatric | 22F | GPSC19 | 34.76 | 2,068,456 | 98 | 42,588 | SAMN48917785 |
| CSPN0279 | CASPER | Calgary | Pediatric | 22F | GPSC19 | 37.33 | 2,055,225 | 101 | 39,151 | SAMN48917784 |
| CSPN0281 | CASPER | Calgary | Pediatric | 23F | GPSC14 | 30.87 | 2,117,878 | 132 | 35,203 | SAMN48917786 |
| CSPN0549 | CASPER | Calgary | Adult | 4 | GPSC27 | 60.18 | 2,127,176 | 61 | 65,522 | SAMN48918049 |
| CSPN0550 | CASPER | Calgary | Adult | 19A | GPSC4 | 55.17 | 2,055,702 | 72 | 49,114 | SAMN48918050 |
| CSPN0551 | CASPER | Calgary | Adult | 19A | GPSC4 | 58.99 | 2,056,956 | 60 | 64,391 | SAMN48918051 |
| CSPN0553 | CASPER | Calgary | Adult | 3 | GPSC12 | 49.1 | 2,015,378 | 111 | 45,782 | SAMN48918053 |
| CSPN0554 | CASPER | Calgary | Adult | 19A | GPSC10 | 52.12 | 2,099,114 | 140 | 40,942 | SAMN48918054 |
| CSPN0283 | CASPER | Calgary | Pediatric | 15A | GPSC9 | 57.07 | 2,071,894 | 95 | 51,623 | SAMN48917788 |
| CSPN0285 | CASPER | Calgary | Pediatric | 38 | GPSC38 | 23.94 | 2,114,328 | 92 | 44,469 | SAMN48917790 |
| CSPN0284 | CASPER | Calgary | Pediatric | 15B | GPSC11 | 43.3 | 2,101,022 | 126 | 30,245 | SAMN48917789 |
| CSPN0557 | CASPER | Calgary | Adult | 8 | GPSC98 | 48.79 | 1,993,430 | 57 | 67,743 | SAMN48918057 |
| CSPN0556 | CASPER | Calgary | Adult | 3 | GPSC12 | 66.07 | 2,022,023 | 75 | 54,605 | SAMN48918056 |
| CSPN0555 | CASPER | Calgary | Adult | 4 | GPSC27 | 65.71 | 2,118,785 | 102 | 41,998 | SAMN48918055 |
| CSPN0287 | CASPER | Calgary | Pediatric | 19F | GPSC119 | 380.71 | 2,118,329 | 105 | 49,017 | SAMN48917792 |
| CSPN0292 | CASPER | Calgary | Pediatric | 3 | GPSC12 | 42.44 | 2,025,048 | 60 | 73,999 | SAMN48917797 |
| CSPN0567 | CASPER | Calgary | Adult | 3 | GPSC12 | 57.72 | 2,005,964 | 107 | 40,094 | SAMN48918067 |
| CSPN0301 | CASPER | Calgary | Pediatric | NT | GPSC81 | 31.01 | 2,184,587 | 160 | 31,259 | SAMN48917806 |
| CSPN0558 | CASPER | Calgary | Adult | 19A | GPSC4 | 48.82 | 2,099,487 | 108 | 44,307 | SAMN48918058 |
| CSPN0560 | CASPER | Calgary | Adult | 19A | GPSC4 | 54.21 | 2,055,106 | 105 | 40,075 | SAMN48918060 |
| CSPN0559 | CASPER | Calgary | Adult | 14 | GPSC9 | 57.69 | 2,121,814 | 91 | 51,793 | SAMN48918059 |
| CSPN0561 | CASPER | Calgary | Adult | 3 | GPSC12 | 56.45 | 1,991,336 | 63 | 69,700 | SAMN48918061 |
| CSPN0288 | CASPER | Calgary | Pediatric | 3 | GPSC12 | 50.33 | 2,027,262 | 82 | 49,747 | SAMN48917793 |
| CSPN0289 | CASPER | Calgary | Pediatric | 23B | GPSC7 | 38.11 | 2,059,342 | 64 | 68,065 | SAMN48917794 |
| CSPN0290 | CASPER | Calgary | Pediatric | 33F | GPSC3 | 34.54 | 2,050,983 | 58 | 74,711 | SAMN48917795 |
| CSPN0562 | CASPER | Calgary | Adult | 4 | GPSC27 | 55.85 | 2,095,602 | 117 | 36,429 | SAMN48918062 |
| CSPN0291 | CASPER | Calgary | Pediatric | 19A | GPSC27 | 35.09 | 2,105,256 | 99 | 45,871 | SAMN48917796 |
| CSPN0563 | CASPER | Calgary | Adult | 3 | GPSC12 | 52.88 | 1,969,058 | 91 | 44,924 | SAMN48918063 |
| CSPN0566 | CASPER | Calgary | Adult | 19A | GPSC10 | 54.25 | 2,101,738 | 107 | 43,754 | SAMN48918066 |
| CSPN0564 | CASPER | Calgary | Adult | 4 | GPSC27 | 50.04 | 2,102,644 | 91 | 55,693 | SAMN48918064 |
| CSPN0565 | CASPER | Calgary | Adult | 4 | GPSC27 | 48.76 | 2,099,726 | 126 | 34,480 | SAMN48918065 |
| CSPN0294 | CASPER | Calgary | Pediatric | 35B | GPSC75 | 48.42 | 2,075,215 | 127 | 40,006 | SAMN48917799 |
| CSPN0293 | CASPER | Calgary | Pediatric | 33F | GPSC3 | 46.8 | 1,974,146 | 61 | 61,890 | SAMN48917798 |
| CSPN0295 | CASPER | Calgary | Pediatric | 15C | GPSC6 | 351.91 | 2,125,135 | 72 | 68,674 | SAMN48917800 |
| CSPN0568 | CASPER | Calgary | Adult | 8 | GPSC98 | 50.71 | 1,992,221 | 56 | 91,709 | SAMN48918068 |
| CSPN0569 | CASPER | Calgary | Adult | 3 | GPSC12 | 52.86 | 1,989,416 | 67 | 68,378 | SAMN48918069 |
| CSPN0296 | CASPER | Calgary | Pediatric | 15A | GPSC9 | 62.97 | 2,109,044 | 133 | 32,219 | SAMN48917801 |
| CSPN0297 | CASPER | Calgary | Pediatric | 35B | GPSC75 | 59.12 | 2,071,936 | 113 | 46,781 | SAMN48917802 |
| CSPN0299 | CASPER | Calgary | Pediatric | 12F | GPSC32 | 55.02 | 2,010,484 | 77 | 56,530 | SAMN48917804 |
| CSPN0298 | CASPER | Calgary | Pediatric | 8 | GPSC98 | 43.55 | 1,991,480 | 64 | 56,754 | SAMN48917803 |
| CSPN0300 | CASPER | Calgary | Pediatric | 22F | GPSC19 | 74.29 | 2,079,373 | 70 | 50,162 | SAMN48917805 |
| CSPN0570 | CASPER | Calgary | Adult | 3 | GPSC12 | 52.39 | 1,989,039 | 73 | 51,152 | SAMN48918070 |
| CSPN0571 | CASPER | Calgary | Adult | 4 | GPSC27 | 50.47 | 2,099,530 | 95 | 37,031 | SAMN48918071 |
| CSPN0572 | CASPER | Calgary | Adult | 4 | GPSC27 | 50.23 | 2,103,498 | 130 | 34,320 | SAMN48918072 |
| CSPN0302 | CASPER | Calgary | Pediatric | 22F | GPSC19 | 32.58 | 2,097,356 | 82 | 48,375 | SAMN48917807 |
| CSPN0305 | CASPER | Calgary | Pediatric | 38 | GPSC38 | 36.17 | 2,120,265 | 144 | 29,534 | SAMN48917809 |
| CSPN0304 | CASPER | Calgary | Pediatric | 23F | GPSC196 | 38.59 | 2,105,537 | 91 | 45,739 | SAMN48917808 |
| CSPN0307 | CASPER | Calgary | Pediatric | 31 | GPSC57 | 37.31 | 2,015,903 | 147 | 31,108 | SAMN48917811 |
| CSPN0573 | CASPER | Calgary | Adult | 19A | GPSC27 | 57.25 | 2,148,012 | 138 | 38,130 | SAMN48918073 |
| CSPN0308 | CASPER | Calgary | Pediatric | 15A | GPSC6 | 48.05 | 2,101,187 | 84 | 47,761 | SAMN48917812 |
| CSPN0306 | CASPER | Calgary | Pediatric | 33F | GPSC3 | 38.42 | 2,048,158 | 93 | 47,508 | SAMN48917810 |
| NSPN0005 | TIBDN | Toronto | Pediatric | 15C | GPSC11 | 55.84 | 2,107,438 | 92 | 49,295 | SAMN48929170 |
| NSPN0147 | TIBDN | Toronto | Pediatric | 3 | GPSC12 | 48.45 | 2,016,910 | 67 | 80,340 | SAMN48929267 |
| NSPN0078 | TIBDN | Toronto | Pediatric | 7F | GPSC15 | 62.84 | 1,999,505 | 68 | 69,531 | SAMN48929226 |
| NSPN0077 | TIBDN | Toronto | Pediatric | 19A | GPSC1 | 55.06 | 2,055,303 | 112 | 40,560 | SAMN48929225 |
| NSPN0099 | TIBDN | Toronto | Pediatric | 19A | GPSC27 | 42.86 | 2,142,802 | 85 | 66,395 | SAMN48929246 |
| NSPN0056 | TIBDN | Toronto | Pediatric | 3 | GPSC12 | 45.95 | 2,015,027 | 57 | 68,876 | SAMN48929206 |
| NSPN0079 | TIBDN | Toronto | Pediatric | 15B | GPSC4 | 63.92 | 2,062,556 | 65 | 61,695 | SAMN48929227 |
| NSPN0014 | TIBDN | Toronto | Pediatric | 8 | GPSC224 | 50.32 | 2,033,102 | 65 | 66,985 | SAMN48929178 |
| NSPN0025 | TIBDN | Toronto | Pediatric | 4 | GPSC27 | 52.2 | 2,105,187 | 101 | 50,382 | SAMN48929187 |
| NSPN0045 | TIBDN | Toronto | Pediatric | 3 | GPSC12 | 49.86 | 2,023,212 | 36 | 117,118 | SAMN48929197 |
| NSPN0008 | TIBDN | Toronto | Pediatric | 19A | GPSC27 | 52.41 | 2,136,432 | 97 | 39,818 | SAMN48929173 |
| NSPN0022 | TIBDN | Toronto | Pediatric | 18C | GPSC50 | 65.86 | 2,114,656 | 87 | 56,608 | SAMN48929184 |
| NSPN0111 | TIBDN | Toronto | Pediatric | 3 | GPSC12 | 54.45 | 1,984,513 | 106 | 32,139 | SAMN48929258 |
| NSPN0037 | TIBDN | Toronto | Pediatric | 19A | GPSC1 | 53.91 | 2,057,917 | 86 | 56,258 | SAMN48929195 |
| NSPN0044 | TIBDN | Toronto | Pediatric | 7F | GPSC15 | 49.32 | 1,998,708 | 78 | 50,314 | SAMN48929196 |
| NSPN0089 | TIBDN | Toronto | Pediatric | 15B | GPSC11 | 63.44 | 2,099,694 | 112 | 43,810 | SAMN48929237 |
| NSPN0084 | TIBDN | Toronto | Pediatric | 22F | GPSC19 | 60.71 | 2,030,062 | 83 | 46,530 | SAMN48929232 |
| NSPN0123 | TIBDN | Toronto | Pediatric | 7F | GPSC15 | 52.08 | 1,998,328 | 74 | 58,479 | SAMN48929260 |
| NSPN0027 | TIBDN | Toronto | Pediatric | 3 | GPSC83 | 55.54 | 1,980,122 | 33 | 171,505 | SAMN48929189 |
| NSPN0007 | TIBDN | Toronto | Pediatric | 7F | GPSC15 | 32.45 | 1,997,018 | 60 | 70,375 | SAMN48929172 |
| NSPN0122 | TIBDN | Toronto | Pediatric | 19A | GPSC27 | 51.78 | 2,145,836 | 97 | 48,160 | SAMN48929259 |
| NSPN0110 | TIBDN | Toronto | Pediatric | 19A | GPSC10 | 50.67 | 2,073,206 | 102 | 45,189 | SAMN48929257 |
| NSPN0047 | TIBDN | Toronto | Pediatric | 23F | GPSC1 | 58.85 | 2,054,537 | 69 | 60,472 | SAMN48929199 |
| NSPN0083 | TIBDN | Toronto | Pediatric | 7F | GPSC15 | 66.92 | 1,993,228 | 83 | 45,487 | SAMN48929231 |
| NSPN0036 | TIBDN | Toronto | Pediatric | 15A | GPSC4 | 60.31 | 2,119,053 | 104 | 46,078 | SAMN48929194 |
| NSPN0028 | TIBDN | Toronto | Pediatric | 22F | GPSC19 | 49.73 | 2,138,923 | 89 | 54,423 | SAMN48929190 |
| NSPN0055 | TIBDN | Toronto | Pediatric | 19A | GPSC619 | 68.8 | 2,139,157 | 116 | 35,201 | SAMN48929205 |
| NSPN0080 | TIBDN | Toronto | Pediatric | 35F | GPSC323 | 62.1 | 2,085,560 | 86 | 58,934 | SAMN48929228 |
| NSPN0048 | TIBDN | Toronto | Pediatric | 19A | GPSC10 | 55.7 | 2,111,327 | 110 | 37,917 | SAMN48929200 |
| NSPN0061 | TIBDN | Toronto | Pediatric | 19A | GPSC1 | 760.44 | 2,043,106 | 41 | 126,416 | SAMN48929211 |
| NSPN0103 | TIBDN | Toronto | Pediatric | 19A | GPSC4 | 56.09 | 2,146,995 | 63 | 82,596 | SAMN48929250 |
| NSPN0010 | TIBDN | Toronto | Pediatric | 15C | GPSC11 | 57.47 | 2,076,169 | 99 | 32,640 | SAMN48929175 |
| NSPN0088 | TIBDN | Toronto | Pediatric | 19A | GPSC97 | 67.96 | 2,126,162 | 120 | 35,130 | SAMN48929236 |
| NSPN0057 | TIBDN | Toronto | Pediatric | 15C | GPSC11 | 65.4 | 2,089,276 | 104 | 40,468 | SAMN48929207 |
| NSPN0098 | TIBDN | Toronto | Pediatric | 22F | GPSC19 | 55 | 2,072,291 | 149 | 26,161 | SAMN48929245 |
| NSPN0146 | TIBDN | Toronto | Pediatric | 23A | GPSC5 | 43.12 | 2,118,230 | 112 | 40,047 | SAMN48929266 |
| NSPN0052 | TIBDN | Toronto | Pediatric | 7F | GPSC15 | 64.54 | 1,997,630 | 91 | 40,249 | SAMN48929203 |
| NSPN0068 | TIBDN | Toronto | Pediatric | 19A | GPSC1 | 62.07 | 2,051,424 | 125 | 32,756 | SAMN48929218 |
| NSPN0107 | TIBDN | Toronto | Pediatric | 35B | GPSC75 | 56.46 | 2,089,770 | 78 | 55,505 | SAMN48929254 |
| NSPN0092 | TIBDN | Toronto | Pediatric | 19F | GPSC175 | 62.9 | 2,075,812 | 82 | 49,751 | SAMN48929240 |
| NSPN0012 | TIBDN | Toronto | Pediatric | 7F | GPSC15 | 54.25 | 1,995,380 | 66 | 57,955 | SAMN48929177 |
| NSPN0148 | TIBDN | Toronto | Pediatric | 22F | GPSC19 | 40.95 | 2,071,611 | 105 | 45,805 | SAMN48929268 |
| NSPN0156 | TIBDN | Toronto | Pediatric | 19A | GPSC27 | 54.67 | 2,145,797 | 110 | 36,925 | SAMN48929276 |
| NSPN0149 | TIBDN | Toronto | Pediatric | 19A | GPSC97 | 41.48 | 2,130,419 | 82 | 58,583 | SAMN48929269 |
| NSPN0067 | TIBDN | Toronto | Pediatric | 10A | GPSC35 | 40.48 | 2,124,087 | 62 | 63,855 | SAMN48929217 |
| NSPN0011 | TIBDN | Toronto | Pediatric | 19A | GPSC27 | 62.73 | 2,148,991 | 105 | 52,361 | SAMN48929176 |
| NSPN0091 | TIBDN | Toronto | Pediatric | 22F | GPSC19 | 84.01 | 2,038,054 | 81 | 55,144 | SAMN48929239 |
| NSPN0062 | TIBDN | Toronto | Pediatric | 22F | GPSC19 | 48.38 | 2,113,017 | 74 | 48,007 | SAMN48929212 |
| NSPN0069 | TIBDN | Toronto | Pediatric | 31 | GPSC12 | 61.4 | 2,024,205 | 65 | 60,400 | SAMN48929219 |
| NSPN0102 | TIBDN | Toronto | Pediatric | 19A | GPSC1 | 55.48 | 2,054,290 | 58 | 76,655 | SAMN48929249 |
| NSPN0105 | TIBDN | Toronto | Pediatric | 22F | GPSC19 | 55.43 | 2,065,161 | 78 | 50,131 | SAMN48929252 |
| NSPN0053 | TIBDN | Toronto | Pediatric | 7F | GPSC15 | 60.71 | 2,001,074 | 96 | 41,690 | SAMN48929204 |
| NSPN0090 | TIBDN | Toronto | Pediatric | 3 | GPSC12 | 56.36 | 2,042,750 | 35 | 142,747 | SAMN48929238 |
| NSPN0059 | TIBDN | Toronto | Pediatric | 3 | GPSC12 | 36.96 | 2,009,643 | 25 | 178,762 | SAMN48929209 |
| NSPN0060 | TIBDN | Toronto | Pediatric | 3 | GPSC12 | 49.22 | 2,020,537 | 46 | 129,067 | SAMN48929210 |
| NSPN0085 | TIBDN | Toronto | Pediatric | 3 | GPSC12 | 69.01 | 2,023,440 | 66 | 79,683 | SAMN48929233 |
| NSPN0106 | TIBDN | Toronto | Pediatric | 19A | GPSC27 | 50.85 | 2,105,948 | 130 | 33,974 | SAMN48929253 |
| NSPN0066 | TIBDN | Toronto | Pediatric | 19A | GPSC27 | 292.06 | 2,176,034 | 93 | 56,143 | SAMN48929216 |
| NSPN0063 | TIBDN | Toronto | Pediatric | 19F | GPSC1 | 61.99 | 2,052,151 | 75 | 52,987 | SAMN48929213 |
| NSPN0070 | TIBDN | Toronto | Pediatric | 15C | GPSC11 | 67.77 | 2,100,946 | 117 | 36,192 | SAMN48929220 |
| NSPN0108 | TIBDN | Toronto | Pediatric | 9N | GPSC16 | 56.38 | 2,084,702 | 98 | 56,340 | SAMN48929255 |
| NSPN0017 | TIBDN | Toronto | Pediatric | 35B | GPSC75 | 55.81 | 2,101,827 | 118 | 39,333 | SAMN48929180 |
| NSPN0009 | TIBDN | Toronto | Pediatric | 19A | GPSC27 | 51.03 | 2,138,834 | 96 | 55,418 | SAMN48929174 |
| NSPN0021 | TIBDN | Toronto | Pediatric | 19A | GPSC6 | 62.15 | 2,133,924 | 68 | 64,773 | SAMN48929183 |
| NSPN0093 | TIBDN | Toronto | Pediatric | 11A | GPSC3 | 97.56 | 2,020,589 | 74 | 53,981 | SAMN48929241 |
| NSPN0006 | TIBDN | Toronto | Pediatric | 19A | GPSC10 | 62.18 | 2,098,038 | 98 | 60,201 | SAMN48929171 |
| NSPN0035 | TIBDN | Toronto | Pediatric | 19A | GPSC1 | 61 | 2,096,998 | 62 | 67,052 | SAMN48929193 |
| NSPN0081 | TIBDN | Toronto | Pediatric | 6C | GPSC29 | 57.72 | 2,090,123 | 153 | 29,134 | SAMN48929229 |
| NSPN0064 | TIBDN | Toronto | Pediatric | 21 | GPSC99 | 71.12 | 2,128,668 | 90 | 45,691 | SAMN48929214 |
| NSPN0109 | TIBDN | Toronto | Pediatric | 15B | GPSC4 | 48.69 | 2,101,690 | 72 | 52,669 | SAMN48929256 |
| NSPN0026 | TIBDN | Toronto | Pediatric | 22F | GPSC19 | 64.22 | 2,035,359 | 95 | 42,912 | SAMN48929188 |
| NSPN0018 | TIBDN | Toronto | Pediatric | 19A | GPSC1 | 58.94 | 2,080,677 | 88 | 44,626 | SAMN48929181 |
| NSPN0073 | TIBDN | Toronto | Pediatric | 19A | GPSC1 | 66.85 | 2,056,890 | 90 | 44,618 | SAMN48929223 |
| NSPN0158 | TIBDN | Toronto | Pediatric | 15C | GPSC11 | 43.94 | 2,090,558 | 62 | 91,347 | SAMN48929278 |
| NSPN0071 | TIBDN | Toronto | Pediatric | 6C | GPSC76 | 78.96 | 2,113,275 | 79 | 54,075 | SAMN48929221 |
| NSPN0087 | TIBDN | Toronto | Pediatric | 7F | GPSC15 | 51.81 | 1,994,805 | 76 | 47,438 | SAMN48929235 |
| NSPN0016 | TIBDN | Toronto | Pediatric | 23F | GPSC7 | 30.21 | 2,070,013 | 38 | 139,921 | SAMN48929179 |
| NSPN0046 | TIBDN | Toronto | Pediatric | 7F | GPSC15 | 66.81 | 1,996,173 | 81 | 59,111 | SAMN48929198 |
| NSPN0050 | TIBDN | Toronto | Pediatric | 19F | GPSC119 | 52.59 | 2,085,098 | 89 | 46,447 | SAMN48929202 |
| NSPN0002 | TIBDN | Toronto | Pediatric | 10F | GPSCNA | 57.18 | 2,124,022 | 103 | 40,505 | SAMN48929167 |
| NSPN0157 | TIBDN | Toronto | Pediatric | 10A | GPSC35 | 45.47 | 2,073,514 | 44 | 83,350 | SAMN48929277 |
| NSPN0104 | TIBDN | Toronto | Pediatric | 22F | GPSC19 | 53.97 | 2,037,216 | 63 | 55,115 | SAMN48929251 |
| NSPN0086 | TIBDN | Toronto | Pediatric | 19A | GPSC4 | 49.15 | 2,093,424 | 102 | 53,483 | SAMN48929234 |
| NSPN0100 | TIBDN | Toronto | Pediatric | 18C | GPSC107 | 53.07 | 2,114,205 | 52 | 113,686 | SAMN48929247 |
| NSPN0001 | TIBDN | Toronto | Pediatric | 23B | GPSC7 | 50.2 | 2,070,884 | 65 | 79,069 | SAMN48929166 |
| NSPN0082 | TIBDN | Toronto | Pediatric | 7F | GPSC15 | 71.83 | 1,999,153 | 110 | 32,476 | SAMN48929230 |
| NSPN0094 | TIBDN | Toronto | Pediatric | 3 | GPSC12 | 31.51 | 1,968,164 | 23 | 142,799 | SAMN48929242 |
| NSPN0074 | TIBDN | Toronto | Pediatric | 19A | GPSC10 | 71.44 | 2,072,884 | 106 | 52,808 | SAMN48929224 |
| NSPN0095 | TIBDN | Toronto | Pediatric | 19A | GPSC18 | 61.57 | 2,090,066 | 87 | 42,965 | SAMN48929243 |
| NSPN0024 | TIBDN | Toronto | Pediatric | 15B | GPSC229 | 63.37 | 2,036,335 | 89 | 47,982 | SAMN48929186 |
| NSPN0019 | TIBDN | Toronto | Pediatric | 33A | GPSC3 | 44.13 | 2,067,197 | 57 | 103,458 | SAMN48929182 |
| NSPN0004 | TIBDN | Toronto | Pediatric | 7F | GPSC15 | 57.65 | 1,994,535 | 74 | 57,388 | SAMN48929169 |
| NSPN0029 | TIBDN | Toronto | Pediatric | 19A | GPSC27 | 59.04 | 2,180,403 | 144 | 49,230 | SAMN48929191 |
| NSPN0101 | TIBDN | Toronto | Pediatric | 11A | GPSC3 | 39.63 | 2,054,141 | 57 | 75,516 | SAMN48929248 |
| NSPN0124 | TIBDN | Toronto | Pediatric | 15B | GPSC11 | 57.6 | 2,101,475 | 67 | 62,332 | SAMN48929261 |
| NSPN0072 | TIBDN | Toronto | Pediatric | 22F | GPSC19 | 65.09 | 2,051,693 | 74 | 55,493 | SAMN48929222 |
| NSPN0065 | TIBDN | Toronto | Pediatric | 22F | GPSC19 | 61.68 | 2,111,553 | 101 | 36,374 | SAMN48929215 |
| NSPN0058 | TIBDN | Toronto | Pediatric | 33A | GPSC3 | 60.99 | 2,056,630 | 72 | 50,384 | SAMN48929208 |
| NSPN0096 | TIBDN | Toronto | Pediatric | 33A | GPSC3 | 63.04 | 2,068,665 | 28 | 146,777 | SAMN48929244 |
| NSPN0049 | TIBDN | Toronto | Pediatric | 19A | GPSC10 | 60.77 | 2,094,911 | 81 | 55,556 | SAMN48929201 |
| NSPN0003 | TIBDN | Toronto | Pediatric | 15C | GPSC11 | 60.37 | 2,093,036 | 78 | 73,578 | SAMN48929168 |
| NSPN0034 | TIBDN | Toronto | Pediatric | 22F | GPSC19 | 58.19 | 2,035,866 | 77 | 54,648 | SAMN48929192 |
| NSPN0023 | TIBDN | Toronto | Pediatric | 19A | GPSC27 | 57.84 | 2,147,590 | 73 | 50,658 | SAMN48929185 |
| NSPN0188 | TIBDN | Toronto | Pediatric | 22F | GPSC19 | 34.2 | 2,073,535 | 52 | 89,815 | SAMN48929299 |
| NSPN0230 | TIBDN | Toronto | Pediatric | 7F | GPSC15 | 52.35 | 1,996,364 | 126 | 28,948 | SAMN48929334 |
| NSPN0209 | TIBDN | Toronto | Pediatric | 15C | GPSC4 | 59.9 | 2,118,141 | 126 | 35,370 | SAMN48929317 |
| NSPN0204 | TIBDN | Toronto | Pediatric | 7F | GPSC15 | 61.46 | 1,997,244 | 94 | 41,071 | SAMN48929313 |
| NSPN0228 | TIBDN | Toronto | Pediatric | 19A | GPSC27 | 67.79 | 2,101,332 | 72 | 55,253 | SAMN48929332 |
| NSPN0126 | TIBDN | Toronto | Pediatric | 16F | GPSC135 | 53.43 | 2,090,599 | 62 | 80,512 | SAMN48929263 |
| NSPN0197 | TIBDN | Toronto | Pediatric | 7F | GPSC15 | 54.97 | 1,998,623 | 133 | 28,165 | SAMN48929306 |
| NSPN0150 | TIBDN | Toronto | Pediatric | 6C | GPSC111 | 56.02 | 2,055,356 | 44 | 99,210 | SAMN48929270 |
| NSPN0151 | TIBDN | Toronto | Pediatric | 19A | GPSC1 | 47.37 | 2,089,814 | 57 | 71,130 | SAMN48929271 |
| NSPN0270 | TIBDN | Toronto | Pediatric | 15B | GPSC11 | 54.37 | 2,145,972 | 75 | 73,141 | SAMN48929369 |
| NSPN0243 | TIBDN | Toronto | Pediatric | 35B | GPSC59 | 53.36 | 2,007,772 | 72 | 53,624 | SAMN48929346 |
| NSPN0152 | TIBDN | Toronto | Pediatric | 23F | GPSC16 | 32.75 | 2,147,237 | 107 | 41,005 | SAMN48929272 |
| NSPN0168 | TIBDN | Toronto | Pediatric | 19A | GPSC27 | 52.9 | 2,108,077 | 95 | 52,403 | SAMN48929281 |
| NSPN0187 | TIBDN | Toronto | Pediatric | 7A | GPSC15 | 43.52 | 1,999,313 | 48 | 101,599 | SAMN48929298 |
| NSPN0185 | TIBDN | Toronto | Pediatric | 16F | GPSC135 | 57.54 | 2,090,275 | 98 | 54,783 | SAMN48929296 |
| NSPN0242 | TIBDN | Toronto | Pediatric | 7F | GPSC15 | 49.79 | 1,995,566 | 110 | 34,586 | SAMN48929345 |
| NSPN0212 | TIBDN | Toronto | Pediatric | 22F | GPSC19 | 67.34 | 2,041,200 | 78 | 42,114 | SAMN48929320 |
| NSPN0219 | TIBDN | Toronto | Pediatric | 7F | GPSC15 | 60.61 | 1,995,511 | 78 | 56,759 | SAMN48929327 |
| NSPN0175 | TIBDN | Toronto | Pediatric | 19A | GPSC97 | 46.89 | 2,133,538 | 64 | 64,938 | SAMN48929288 |
| NSPN0161 | TIBDN | Toronto | Pediatric | 9N | GPSC16 | 85.74 | 2,050,602 | 38 | 125,418 | SAMN48929279 |
| NSPN0214 | TIBDN | Toronto | Pediatric | 7F | GPSC15 | 74.11 | 1,993,359 | 107 | 46,358 | SAMN48929322 |
| NSPN0218 | TIBDN | Toronto | Pediatric | 7F | GPSC15 | 56.29 | 1,999,197 | 74 | 54,189 | SAMN48929326 |
| NSPN0234 | TIBDN | Toronto | Pediatric | 19A | GPSC27 | 66.5 | 2,171,107 | 125 | 52,029 | SAMN48929338 |
| NSPN0203 | TIBDN | Toronto | Pediatric | 7F | GPSC15 | 61.51 | 2,069,249 | 152 | 29,912 | SAMN48929312 |
| NSPN0193 | TIBDN | Toronto | Pediatric | 19A | GPSC5 | 64.04 | 2,206,541 | 101 | 51,229 | SAMN48929304 |
| NSPN0198 | TIBDN | Toronto | Pediatric | 19A | GPSC27 | 59.31 | 2,147,571 | 123 | 36,565 | SAMN48929307 |
| NSPN0210 | TIBDN | Toronto | Pediatric | 33A | GPSC3 | 77.75 | 2,065,911 | 56 | 85,714 | SAMN48929318 |
| NSPN0178 | TIBDN | Toronto | Pediatric | 19A | GPSC1 | 61.62 | 2,053,485 | 64 | 66,962 | SAMN48929289 |
| NSPN0233 | TIBDN | Toronto | Pediatric | 19A | GPSC4 | 55.44 | 2,143,757 | 112 | 55,762 | SAMN48929337 |
| NSPN0213 | TIBDN | Toronto | Pediatric | 19A | GPSC27 | 61.24 | 2,144,241 | 101 | 47,231 | SAMN48929321 |
| NSPN0181 | TIBDN | Toronto | Pediatric | 19A | GPSC59 | 47.16 | 2,017,099 | 62 | 56,150 | SAMN48929292 |
| NSPN0199 | TIBDN | Toronto | Pediatric | 19A | GPSC1 | 60.49 | 2,054,698 | 92 | 56,705 | SAMN48929308 |
| NSPN0127 | TIBDN | Toronto | Pediatric | 19A | GPSC4 | 53.58 | 2,055,605 | 50 | 122,788 | SAMN48929264 |
| NSPN0191 | TIBDN | Toronto | Pediatric | 15B | GPSC11 | 60.86 | 2,092,818 | 85 | 56,199 | SAMN48929302 |
| NSPN0192 | TIBDN | Toronto | Pediatric | 7F | GPSC15 | 55.34 | 1,999,131 | 118 | 33,687 | SAMN48929303 |
| NSPN0222 | TIBDN | Toronto | Pediatric | 19A | GPSC27 | 68.25 | 2,150,664 | 73 | 63,844 | SAMN48929330 |
| NSPN0250 | TIBDN | Toronto | Pediatric | 3 | GPSC12 | 28.71 | 2,097,749 | 37 | 97,726 | SAMN48929353 |
| NSPN0248 | TIBDN | Toronto | Pediatric | 19A | GPSC932 | 55.31 | 2,090,489 | 60 | 72,432 | SAMN48929351 |
| NSPN0173 | TIBDN | Toronto | Pediatric | 15C | GPSC11 | 55.29 | 2,172,680 | 97 | 49,093 | SAMN48929286 |
| NSPN0182 | TIBDN | Toronto | Pediatric | 7F | GPSC15 | 37.29 | 2,069,710 | 94 | 48,405 | SAMN48929293 |
| NSPN0155 | TIBDN | Toronto | Pediatric | 7F | GPSC15 | 48.35 | 2,000,818 | 70 | 65,393 | SAMN48929275 |
| NSPN0174 | TIBDN | Toronto | Pediatric | 7F | GPSC15 | 51.89 | 1,996,982 | 90 | 47,512 | SAMN48929287 |
| NSPN0196 | TIBDN | Toronto | Pediatric | 7F | GPSC15 | 58.79 | 1,995,757 | 106 | 41,643 | SAMN48929305 |
| NSPN0206 | TIBDN | Toronto | Pediatric | 3 | GPSC12 | 58.68 | 2,024,103 | 71 | 56,744 | SAMN48929315 |
| NSPN0162 | TIBDN | Toronto | Pediatric | 19A | GPSC27 | 46.01 | 2,171,375 | 82 | 60,042 | SAMN48929280 |
| NSPN0183 | TIBDN | Toronto | Pediatric | 7F | GPSC15 | 43.82 | 1,995,896 | 90 | 48,963 | SAMN48929294 |
| NSPN0231 | TIBDN | Toronto | Pediatric | 19A | GPSC4 | 62.59 | 2,145,703 | 114 | 51,418 | SAMN48929335 |
| NSPN0221 | TIBDN | Toronto | Pediatric | 19F | GPSC101 | 63.84 | 2,083,267 | 40 | 81,648 | SAMN48929329 |
| NSPN0189 | TIBDN | Toronto | Pediatric | 19A | GPSC4 | 58.27 | 2,119,991 | 88 | 49,400 | SAMN48929300 |
| NSPN0241 | TIBDN | Toronto | Pediatric | 19A | GPSC1 | 53.37 | 2,055,308 | 135 | 33,845 | SAMN48929344 |
| NSPN0205 | TIBDN | Toronto | Pediatric | 11A | GPSC43 | 55.18 | 2,202,265 | 130 | 48,969 | SAMN48929314 |
| NSPN0220 | TIBDN | Toronto | Pediatric | 23B | GPSC7 | 55 | 2,061,409 | 50 | 73,975 | SAMN48929328 |
| NSPN0128 | TIBDN | Toronto | Pediatric | 19A | GPSC1 | 37.48 | 2,071,379 | 55 | 94,603 | SAMN48929265 |
| NSPN0229 | TIBDN | Toronto | Pediatric | 19A | GPSC27 | 60.78 | 2,184,230 | 142 | 32,733 | SAMN48929333 |
| NSPN0125 | TIBDN | Toronto | Pediatric | 6B | GPSC120 | 50.37 | 2,238,018 | 68 | 73,743 | SAMN48929262 |
| NSPN0232 | TIBDN | Toronto | Pediatric | 21 | GPSC99 | 60.39 | 2,126,478 | 48 | 88,588 | SAMN48929336 |
| NSPN0216 | TIBDN | Toronto | Pediatric | 15C | GPSC11 | 57.6 | 2,090,105 | 79 | 62,332 | SAMN48929324 |
| NSPN0249 | TIBDN | Toronto | Pediatric | 10A | GPSC35 | 47.71 | 2,068,603 | 55 | 66,064 | SAMN48929352 |
| NSPN0202 | TIBDN | Toronto | Pediatric | 19A | GPSC27 | 62.25 | 2,145,445 | 144 | 31,253 | SAMN48929311 |
| NSPN0211 | TIBDN | Toronto | Pediatric | 23A | GPSC5 | 62.77 | 2,115,981 | 122 | 43,855 | SAMN48929319 |
| NSPN0215 | TIBDN | Toronto | Pediatric | 7F | GPSC15 | 57.04 | 1,995,717 | 109 | 36,396 | SAMN48929323 |
| NSPN0186 | TIBDN | Toronto | Pediatric | 19F | GPSC119 | 52.13 | 2,106,247 | 85 | 49,541 | SAMN48929297 |
| NSPN0227 | TIBDN | Toronto | Pediatric | 15B | GPSC4 | 76.23 | 2,082,408 | 76 | 56,124 | SAMN48929331 |
| NSPN0180 | TIBDN | Toronto | Pediatric | 35B | GPSC75 | 59.46 | 2,073,977 | 100 | 45,509 | SAMN48929291 |
| NSPN0200 | TIBDN | Toronto | Pediatric | 35B | GPSC59 | 74.64 | 2,040,877 | 75 | 51,825 | SAMN48929309 |
| NSPN0169 | TIBDN | Toronto | Pediatric | 7F | GPSC15 | 52.82 | 1,997,794 | 67 | 69,490 | SAMN48929282 |
| NSPN0179 | TIBDN | Toronto | Pediatric | 15A | GPSC9 | 57.42 | 2,147,147 | 77 | 67,089 | SAMN48929290 |
| NSPN0208 | TIBDN | Toronto | Pediatric | 23B | GPSC102 | 74.41 | 2,104,843 | 79 | 64,252 | SAMN48929316 |
| NSPN0184 | TIBDN | Toronto | Pediatric | 35B | GPSC59 | 58.13 | 2,008,001 | 51 | 69,966 | SAMN48929295 |
| NSPN0190 | TIBDN | Toronto | Pediatric | 35B | GPSC75 | 56.71 | 2,073,772 | 88 | 45,639 | SAMN48929301 |
| NSPN0171 | TIBDN | Toronto | Pediatric | 6C | GPSC111 | 56.58 | 2,071,187 | 73 | 58,619 | SAMN48929284 |
| NSPN0153 | TIBDN | Toronto | Pediatric | 19A | GPSC4 | 59.33 | 2,057,911 | 83 | 65,272 | SAMN48929273 |
| NSPN0201 | TIBDN | Toronto | Pediatric | 19A | GPSC1 | 62.8 | 2,055,684 | 107 | 48,556 | SAMN48929310 |
| NSPN0217 | TIBDN | Toronto | Pediatric | 19A | GPSC146 | 61.1 | 2,171,105 | 104 | 44,760 | SAMN48929325 |
| NSPN0154 | TIBDN | Toronto | Pediatric | 19A | GPSC18 | 53.22 | 2,042,355 | 80 | 50,723 | SAMN48929274 |
| NSPN0172 | TIBDN | Toronto | Pediatric | 19A | GPSC1 | 47.4 | 2,055,241 | 87 | 56,775 | SAMN48929285 |
| NSPN0170 | TIBDN | Toronto | Pediatric | 22F | GPSC19 | 44.97 | 2,037,318 | 65 | 76,381 | SAMN48929283 |
| NSPN0269 | TIBDN | Toronto | Pediatric | 3 | GPSC83 | 52.05 | 1,979,848 | 62 | 83,081 | SAMN48929368 |
| NSPN0327 | TIBDN | Toronto | Pediatric | 7F | GPSC15 | 63.04 | 1,996,647 | 121 | 41,333 | SAMN48929415 |
| NSPN0314 | TIBDN | Toronto | Pediatric | 19A | GPSC27 | 55.57 | 2,144,806 | 145 | 34,208 | SAMN48929402 |
| NSPN0247 | TIBDN | Toronto | Pediatric | 19A | GPSC27 | 61.75 | 2,169,482 | 138 | 41,461 | SAMN48929350 |
| NSPN0299 | TIBDN | Toronto | Pediatric | 19A | GPSC27 | 62.26 | 2,185,698 | 133 | 33,642 | SAMN48929388 |
| NSPN0258 | TIBDN | Toronto | Pediatric | 22F | GPSC6 | 48.14 | 2,105,286 | 103 | 50,244 | SAMN48929361 |
| NSPN0265 | TIBDN | Toronto | Pediatric | 19A | GPSC27 | 51.56 | 2,146,489 | 177 | 27,527 | SAMN48929364 |
| NSPN0302 | TIBDN | Toronto | Pediatric | 19A | GPSC27 | 51.26 | 2,095,088 | 151 | 37,564 | SAMN48929391 |
| NSPN0328 | TIBDN | Toronto | Pediatric | 35B | GPSC75 | 68.14 | 2,110,922 | 105 | 45,183 | SAMN48929416 |
| NSPN0300 | TIBDN | Toronto | Pediatric | 9V | GPSC6 | 454.94 | 2,103,122 | 79 | 75,574 | SAMN48929389 |
| NSPN0315 | TIBDN | Toronto | Pediatric | 8 | GPSC224 | 67.03 | 1,999,192 | 88 | 62,729 | SAMN48929403 |
| NSPN0288 | TIBDN | Toronto | Pediatric | 22F | GPSC19 | 73.77 | 2,084,308 | 60 | 65,304 | SAMN48929381 |
| NSPN0306 | TIBDN | Toronto | Pediatric | 7F | GPSC15 | 60.89 | 2,071,123 | 71 | 55,340 | SAMN48929395 |
| NSPN0244 | TIBDN | Toronto | Pediatric | 7F | GPSC15 | 50.66 | 2,001,146 | 79 | 56,792 | SAMN48929347 |
| NSPN0277 | TIBDN | Toronto | Pediatric | 3 | GPSC51 | 50.23 | 2,032,267 | 71 | 73,178 | SAMN48929375 |
| NSPN0245 | TIBDN | Toronto | Pediatric | 19A | GPSC27 | 55.08 | 2,171,406 | 131 | 37,337 | SAMN48929348 |
| NSPN0304 | TIBDN | Toronto | Pediatric | 19A | GPSC27 | 46.89 | 2,102,932 | 85 | 57,456 | SAMN48929393 |
| NSPN0281 | TIBDN | Toronto | Pediatric | 19A | GPSC27 | 57.08 | 2,138,930 | 102 | 45,167 | SAMN48929377 |
| NSPN0239 | TIBDN | Toronto | Pediatric | 3 | GPSC12 | 71.23 | 2,033,296 | 71 | 70,015 | SAMN48929342 |
| NSPN0319 | TIBDN | Toronto | Pediatric | 19A | GPSC99 | 61.23 | 2,216,901 | 81 | 64,062 | SAMN48929407 |
| NSPN0254 | TIBDN | Toronto | Pediatric | 24B | GPSC44 | 54.81 | 2,085,993 | 81 | 76,011 | SAMN48929357 |
| NSPN0266 | TIBDN | Toronto | Pediatric | 23B | GPSC7 | 52.75 | 2,044,527 | 167 | 26,973 | SAMN48929365 |
| NSPN0307 | TIBDN | Toronto | Pediatric | 19A | GPSC27 | 50.69 | 2,100,332 | 83 | 41,715 | SAMN48929396 |
| NSPN0292 | TIBDN | Toronto | Pediatric | 19A | GPSC27 | 46.12 | 2,140,690 | 124 | 37,122 | SAMN48929382 |
| NSPN0297 | TIBDN | Toronto | Pediatric | 19A | GPSC135 | 67.75 | 2,054,968 | 77 | 43,614 | SAMN48929386 |
| NSPN0278 | TIBDN | Toronto | Pediatric | 23A | GPSC5 | 60.24 | 2,114,364 | 96 | 51,205 | SAMN48929376 |
| NSPN0268 | TIBDN | Toronto | Pediatric | 19A | GPSC27 | 56.73 | 2,146,390 | 109 | 39,058 | SAMN48929367 |
| NSPN0333 | TIBDN | Toronto | Pediatric | 19A | GPSC27 | 64.46 | 2,186,314 | 105 | 36,856 | SAMN48929420 |
| NSPN0236 | TIBDN | Toronto | Pediatric | 35B | GPSC75 | 67.9 | 2,062,263 | 84 | 49,005 | SAMN48929339 |
| NSPN0318 | TIBDN | Toronto | Pediatric | 6C | GPSC29 | 64.66 | 2,094,387 | 130 | 30,442 | SAMN48929406 |
| NSPN0308 | TIBDN | Toronto | Pediatric | 23A | GPSC5 | 50.49 | 2,076,336 | 68 | 66,954 | SAMN48929397 |
| NSPN0309 | TIBDN | Toronto | Pediatric | 3 | GPSC12 | 64.38 | 2,023,414 | 57 | 94,386 | SAMN48929398 |
| NSPN0240 | TIBDN | Toronto | Pediatric | 15B | GPSC11 | 63.97 | 2,096,576 | 92 | 62,332 | SAMN48929343 |
| NSPN0301 | TIBDN | Toronto | Pediatric | 23A | GPSC5 | 57.67 | 2,113,202 | 90 | 54,842 | SAMN48929390 |
| NSPN0273 | TIBDN | Toronto | Pediatric | 7F | GPSC15 | 66.57 | 1,993,604 | 95 | 43,588 | SAMN48929371 |
| NSPN0276 | TIBDN | Toronto | Pediatric | 7F | GPSC15 | 74.82 | 1,998,254 | 74 | 61,392 | SAMN48929374 |
| NSPN0293 | TIBDN | Toronto | Pediatric | 3 | GPSC12 | 47.01 | 2,020,820 | 52 | 81,503 | SAMN48929383 |
| NSPN0251 | TIBDN | Toronto | Pediatric | 19A | GPSC1 | 50.5 | 2,054,816 | 110 | 47,012 | SAMN48929354 |
| NSPN0287 | TIBDN | Toronto | Pediatric | 19A | GPSC4 | 56.73 | 2,102,549 | 111 | 35,919 | SAMN48929380 |
| NSPN0295 | TIBDN | Toronto | Pediatric | 19A | GPSC27 | 59.2 | 2,140,063 | 130 | 34,921 | SAMN48929385 |
| NSPN0298 | TIBDN | Toronto | Pediatric | 19A | GPSC27 | 62.39 | 2,145,753 | 110 | 44,609 | SAMN48929387 |
| NSPN0313 | TIBDN | Toronto | Pediatric | 19A | GPSC27 | 58.42 | 2,091,679 | 103 | 44,478 | SAMN48929401 |
| NSPN0264 | TIBDN | Toronto | Pediatric | 15B | GPSC11 | 57.73 | 2,093,781 | 88 | 56,941 | SAMN48929363 |
| NSPN0274 | TIBDN | Toronto | Pediatric | 7F | GPSC15 | 62.03 | 2,063,743 | 103 | 42,947 | SAMN48929372 |
| NSPN0255 | TIBDN | Toronto | Pediatric | 19A | GPSC1 | 56.06 | 2,055,948 | 66 | 64,138 | SAMN48929358 |
| NSPN0316 | TIBDN | Toronto | Pediatric | 19A | GPSC27 | 61.69 | 2,101,561 | 102 | 46,684 | SAMN48929404 |
| NSPN0320 | TIBDN | Toronto | Pediatric | 15C | GPSC11 | 62.17 | 2,095,539 | 77 | 62,497 | SAMN48929408 |
| NSPN0275 | TIBDN | Toronto | Pediatric | 7F | GPSC15 | 57.77 | 2,070,874 | 120 | 35,285 | SAMN48929373 |
| NSPN0285 | TIBDN | Toronto | Pediatric | 31 | GPSC12 | 59.01 | 2,043,021 | 96 | 49,620 | SAMN48929378 |
| NSPN0237 | TIBDN | Toronto | Pediatric | 3 | GPSC12 | 57.65 | 1,990,725 | 49 | 105,409 | SAMN48929340 |
| NSPN0253 | TIBDN | Toronto | Pediatric | 15A | GPSC9 | 54.21 | 2,093,120 | 105 | 46,640 | SAMN48929356 |
| NSPN0257 | TIBDN | Toronto | Pediatric | 19A | GPSC1 | 43.81 | 2,025,354 | 105 | 56,869 | SAMN48929360 |
| NSPN0246 | TIBDN | Toronto | Pediatric | 22F | GPSC19 | 60.05 | 2,081,219 | 75 | 51,197 | SAMN48929349 |
| NSPN0272 | TIBDN | Toronto | Pediatric | 22F | GPSC19 | 61.42 | 2,082,008 | 97 | 52,461 | SAMN48929370 |
| NSPN0263 | TIBDN | Toronto | Pediatric | 33A | GPSC3 | 58.97 | 2,065,818 | 49 | 74,712 | SAMN48929362 |
| NSPN0305 | TIBDN | Toronto | Pediatric | 11A | GPSC3 | 65.33 | 1,977,755 | 58 | 53,571 | SAMN48929394 |
| NSPN0267 | TIBDN | Toronto | Pediatric | 7F | GPSC15 | 58.93 | 1,996,080 | 114 | 34,730 | SAMN48929366 |
| NSPN0310 | TIBDN | Toronto | Pediatric | 15C | GPSC4 | 62.67 | 2,088,409 | 155 | 26,789 | SAMN48929399 |
| NSPN0303 | TIBDN | Toronto | Pediatric | 22F | GPSC19 | 57.98 | 2,074,587 | 98 | 47,285 | SAMN48929392 |
| NSPN0317 | TIBDN | Toronto | Pediatric | 23A | GPSC5 | 57.08 | 2,073,422 | 84 | 45,817 | SAMN48929405 |
| NSPN0312 | TIBDN | Toronto | Pediatric | 15B | GPSC4 | 75.37 | 2,086,403 | 79 | 55,166 | SAMN48929400 |
| NSPN0238 | TIBDN | Toronto | Pediatric | 19A | GPSC27 | 75.85 | 2,146,948 | 117 | 42,228 | SAMN48929341 |
| NSPN0294 | TIBDN | Toronto | Pediatric | 12F | GPSC55 | 53.7 | 2,080,387 | 98 | 43,656 | SAMN48929384 |
| NSPN0256 | TIBDN | Toronto | Pediatric | 15B | GPSC11 | 37.65 | 2,101,527 | 57 | 69,248 | SAMN48929359 |
| NSPN0286 | TIBDN | Toronto | Pediatric | 15C | GPSC12 | 63.81 | 2,071,023 | 156 | 58,159 | SAMN48929379 |
| NSPN0252 | TIBDN | Toronto | Pediatric | 16F | GPSC135 | 51.68 | 2,092,930 | 66 | 77,798 | SAMN48929355 |
| NSPN0384 | TIBDN | Toronto | Pediatric | 15B | GPSC11 | 52.11 | 2,092,157 | 93 | 54,541 | SAMN48929455 |
| NSPN0376 | TIBDN | Toronto | Pediatric | 22F | GPSC19 | 56.85 | 2,079,087 | 188 | 23,002 | SAMN48929447 |
| NSPN0322 | TIBDN | Toronto | Pediatric | 31 | GPSC253 | 69.99 | 2,022,796 | 77 | 56,807 | SAMN48929410 |
| NSPN0367 | TIBDN | Toronto | Pediatric | 19A | GPSC324 | 49.38 | 2,066,935 | 135 | 33,236 | SAMN48929439 |
| NSPN0338 | TIBDN | Toronto | Pediatric | 19A | GPSC27 | 60.24 | 2,137,735 | 86 | 46,763 | SAMN48929424 |
| NSPN0362 | TIBDN | Toronto | Pediatric | 6C | GPSC89 | 39.13 | 2,061,981 | 189 | 24,648 | SAMN48929437 |
| NSPN0383 | TIBDN | Toronto | Pediatric | 22F | GPSC19 | 38.87 | 2,060,184 | 95 | 59,495 | SAMN48929454 |
| NSPN0352 | TIBDN | Toronto | Pediatric | 19A | GPSC1 | 39.98 | 2,029,732 | 170 | 29,995 | SAMN48929429 |
| NSPN0389 | TIBDN | Toronto | Pediatric | 15C | GPSC11 | 53.68 | 2,101,119 | 100 | 37,513 | SAMN48929459 |
| NSPN0390 | TIBDN | Toronto | Pediatric | 19A | GPSC27 | 64.9 | 2,102,163 | 111 | 44,443 | SAMN48929460 |
| NSPN0371 | TIBDN | Toronto | Pediatric | 15B | GPSC4 | 47.8 | 2,063,067 | 144 | 33,646 | SAMN48929442 |
| NSPN0351 | TIBDN | Toronto | Pediatric | 6B | GPSC185 | 44.57 | 2,249,850 | 125 | 41,279 | SAMN48929428 |
| NSPN0582 | TIBDN | Toronto | Pediatric | 23A | GPSC5 | 47.97 | 2,070,919 | 128 | 37,934 | SAMN48929602 |
| NSPN0356 | TIBDN | Toronto | Pediatric | 10A | GPSC35 | 52.99 | 2,123,721 | 55 | 86,616 | SAMN48929433 |
| NSPN0391 | TIBDN | Toronto | Pediatric | 19A | GPSC27 | 53.07 | 2,096,150 | 128 | 34,906 | SAMN48929461 |
| NSPN0378 | TIBDN | Toronto | Pediatric | 19A | GPSC1 | 50.16 | 2,088,290 | 95 | 45,644 | SAMN48929449 |
| NSPN0358 | TIBDN | Toronto | Pediatric | 15B | GPSC4 | 54.68 | 2,137,880 | 80 | 72,907 | SAMN48929434 |
| NSPN0329 | TIBDN | Toronto | Pediatric | 19A | GPSC27 | 50.38 | 2,156,243 | 82 | 48,131 | SAMN48929417 |
| NSPN0324 | TIBDN | Toronto | Pediatric | 35B | GPSC59 | 64.39 | 2,010,661 | 77 | 49,947 | SAMN48929412 |
| NSPN0386 | TIBDN | Toronto | Pediatric | 7F | GPSC15 | 52.6 | 1,989,243 | 134 | 33,748 | SAMN48929457 |
| NSPN0580 | TIBDN | Toronto | Pediatric | 19A | GPSC27 | 60.62 | 2,140,965 | 150 | 27,453 | SAMN48929601 |
| NSPN0388 | TIBDN | Toronto | Pediatric | 19A | GPSC4 | 52.73 | 2,187,832 | 59 | 109,696 | SAMN48929458 |
| NSPN0325 | TIBDN | Toronto | Pediatric | 15C | GPSC11 | 43.67 | 2,137,677 | 62 | 79,445 | SAMN48929413 |
| NSPN0370 | TIBDN | Toronto | Pediatric | 3 | GPSC12 | 46.57 | 2,000,087 | 65 | 65,612 | SAMN48929441 |
| NSPN0380 | TIBDN | Toronto | Pediatric | 15B | GPSC11 | 33.38 | 2,101,016 | 38 | 91,778 | SAMN48929451 |
| NSPN0385 | TIBDN | Toronto | Pediatric | 22F | GPSC19 | 23.69 | 2,076,331 | 40 | 77,780 | SAMN48929456 |
| NSPN0374 | TIBDN | Toronto | Pediatric | 6C | GPSC29 | 28.6 | 2,079,713 | 97 | 46,662 | SAMN48929445 |
| NSPN0392 | TIBDN | Toronto | Pediatric | 7F | GPSC15 | 54.22 | 1,998,200 | 116 | 33,704 | SAMN48929462 |
| NSPN0355 | TIBDN | Toronto | Pediatric | 19A | GPSC4 | 27.37 | 2,110,448 | 159 | 29,440 | SAMN48929432 |
| NSPN0403 | TIBDN | Toronto | Pediatric | 19A | GPSC4 | 62.68 | 2,143,853 | 134 | 41,903 | SAMN48929470 |
| NSPN0339 | TIBDN | Toronto | Pediatric | 19A | GPSC27 | 26.36 | 2,092,400 | 114 | 39,805 | SAMN48929425 |
| NSPN0354 | TIBDN | Toronto | Pediatric | 19A | GPSC27 | 45.17 | 2,109,281 | 139 | 33,973 | SAMN48929431 |
| NSPN0360 | TIBDN | Toronto | Pediatric | 19A | GPSC27 | 55.37 | 2,146,072 | 109 | 36,823 | SAMN48929436 |
| NSPN0373 | TIBDN | Toronto | Pediatric | 19A | GPSC27 | 64.13 | 2,104,763 | 124 | 31,996 | SAMN48929444 |
| NSPN0340 | TIBDN | Toronto | Pediatric | 19A | GPSC97 | 60.26 | 2,127,640 | 183 | 26,566 | SAMN48929426 |
| NSPN0359 | TIBDN | Toronto | Pediatric | 7F | GPSC15 | 50.01 | 1,996,763 | 99 | 34,741 | SAMN48929435 |
| NSPN0335 | TIBDN | Toronto | Pediatric | 19A | GPSC27 | 43.06 | 2,167,219 | 106 | 45,031 | SAMN48929422 |
| NSPN0577 | TIBDN | Toronto | Pediatric | 19A | GPSC4 | 55.04 | 2,067,390 | 48 | 87,666 | SAMN48929599 |
| NSPN0372 | TIBDN | Toronto | Pediatric | 19A | GPSC27 | 32.14 | 2,131,739 | 92 | 56,656 | SAMN48929443 |
| NSPN0365 | TIBDN | Toronto | Pediatric | 19A | GPSC4 | 45.4 | 2,067,366 | 75 | 59,269 | SAMN48929438 |
| NSPN0350 | TIBDN | Toronto | Pediatric | 19A | GPSC27 | 300.92 | 2,135,424 | 80 | 53,595 | SAMN48929427 |
| NSPN0331 | TIBDN | Toronto | Pediatric | 22F | GPSC19 | 46.38 | 2,100,665 | 77 | 58,573 | SAMN48929419 |
| NSPN0321 | TIBDN | Toronto | Pediatric | 23A | GPSC5 | 61.83 | 2,118,688 | 78 | 56,564 | SAMN48929409 |
| NSPN0375 | TIBDN | Toronto | Pediatric | 22F | GPSC19 | 53.71 | 2,037,845 | 83 | 45,780 | SAMN48929446 |
| NSPN0353 | TIBDN | Toronto | Pediatric | 15B | GPSC11 | 39.25 | 2,097,264 | 69 | 59,943 | SAMN48929430 |
| NSPN0330 | TIBDN | Toronto | Pediatric | 11A | GPSC3 | 57.7 | 2,054,614 | 84 | 61,788 | SAMN48929418 |
| NSPN0578 | TIBDN | Toronto | Pediatric | 15C | GPSC4 | 59.26 | 2,135,660 | 110 | 37,474 | SAMN48929600 |
| NSPN0379 | TIBDN | Toronto | Pediatric | 23B | GPSC5 | 49.95 | 2,136,140 | 151 | 27,388 | SAMN48929450 |
| NSPN0323 | TIBDN | Toronto | Pediatric | 19A | GPSC27 | 60.45 | 2,203,709 | 143 | 44,430 | SAMN48929411 |
| NSPN0402 | TIBDN | Toronto | Pediatric | 38 | GPSC38 | 35.47 | 2,159,356 | 83 | 50,927 | SAMN48929469 |
| NSPN0382 | TIBDN | Toronto | Pediatric | 38 | GPSC38 | 61.73 | 2,115,111 | 137 | 34,782 | SAMN48929453 |
| NSPN0334 | TIBDN | Toronto | Pediatric | 15C | GPSC11 | 38.01 | 2,086,030 | 177 | 22,936 | SAMN48929421 |
| NSPN0369 | TIBDN | Toronto | Pediatric | 34 | GPSC45 | 51.79 | 2,114,368 | 95 | 46,700 | SAMN48929440 |
| NSPN0381 | TIBDN | Toronto | Pediatric | 23B | GPSC7 | 58.95 | 2,024,913 | 120 | 37,857 | SAMN48929452 |
| NSPN0377 | TIBDN | Toronto | Pediatric | 33A | GPSC3 | 37.53 | 2,056,270 | 62 | 67,686 | SAMN48929448 |
| NSPN0326 | TIBDN | Toronto | Pediatric | 7F | GPSC15 | 43.09 | 1,989,109 | 130 | 31,981 | SAMN48929414 |
| NSPN0336 | TIBDN | Toronto | Pediatric | 38 | GPSC38 | 64.2 | 2,126,454 | 97 | 51,027 | SAMN48929423 |
| NSPN0432 | TIBDN | Toronto | Pediatric | 19A | GPSC27 | 45.97 | 2,107,202 | 49 | 135,812 | SAMN48929487 |
| NSPN0425 | TIBDN | Toronto | Pediatric | 3 | GPSC12 | 36.41 | 2,019,918 | 21 | 199,278 | SAMN48929482 |
| NSPN0437 | TIBDN | Toronto | Pediatric | 19A | GPSC932 | 52 | 2,090,919 | 43 | 122,045 | SAMN48929492 |
| NSPN0466 | TIBDN | Toronto | Pediatric | 10A | GPSC35 | 76.55 | 2,070,993 | 84 | 47,612 | SAMN48929518 |
| NSPN0441 | TIBDN | Toronto | Pediatric | 15C | GPSC11 | 71.01 | 2,096,770 | 61 | 74,150 | SAMN48929496 |
| NSPN0446 | TIBDN | Toronto | Pediatric | 23A | GPSC7 | 48.68 | 2,082,470 | 52 | 72,665 | SAMN48929501 |
| NSPN0422 | TIBDN | Toronto | Pediatric | 19A | GPSC27 | 50.87 | 2,144,654 | 86 | 50,156 | SAMN48929479 |
| NSPN0465 | TIBDN | Toronto | Pediatric | 3 | GPSC12 | 63.57 | 2,042,929 | 63 | 68,862 | SAMN48929517 |
| NSPN0573 | TIBDN | Toronto | Pediatric | 3 | GPSC12 | 56.94 | 1,990,226 | 51 | 93,474 | SAMN48929597 |
| NSPN0444 | TIBDN | Toronto | Pediatric | 9N | GPSC16 | 61.66 | 2,083,272 | 52 | 65,532 | SAMN48929499 |
| NSPN0428 | TIBDN | Toronto | Pediatric | 8 | GPSC98 | 51.26 | 1,993,642 | 73 | 47,085 | SAMN48929484 |
| NSPN0431 | TIBDN | Toronto | Pediatric | 22F | GPSC19 | 64.1 | 2,081,212 | 80 | 45,553 | SAMN48929486 |
| NSPN0408 | TIBDN | Toronto | Pediatric | 38 | GPSC38 | 41.99 | 2,160,448 | 90 | 55,551 | SAMN48929475 |
| NSPN0396 | TIBDN | Toronto | Pediatric | 35F | GPSC36 | 43.97 | 2,064,356 | 39 | 89,175 | SAMN48929466 |
| NSPN0464 | TIBDN | Toronto | Pediatric | 10A | GPSC35 | 76.07 | 2,066,871 | 76 | 49,633 | SAMN48929516 |
| NSPN0572 | TIBDN | Toronto | Pediatric | 22F | GPSC19 | 66.94 | 2,082,720 | 106 | 39,833 | SAMN48929596 |
| NSPN0438 | TIBDN | Toronto | Pediatric | 35B | GPSC59 | 66.04 | 2,006,766 | 43 | 86,155 | SAMN48929493 |
| NSPN0435 | TIBDN | Toronto | Pediatric | 22F | GPSC19 | 55.75 | 2,078,629 | 63 | 78,048 | SAMN48929490 |
| NSPN0404 | TIBDN | Toronto | Pediatric | 5 | GPSC8 | 43.74 | 2,125,311 | 71 | 65,469 | SAMN48929471 |
| NSPN0434 | TIBDN | Toronto | Pediatric | 22F | GPSC19 | 61.45 | 2,108,547 | 94 | 39,201 | SAMN48929489 |
| NSPN0419 | TIBDN | Toronto | Pediatric | 38 | GPSC38 | 247.55 | 2,109,859 | 83 | 55,555 | SAMN48929477 |
| NSPN0455 | TIBDN | Toronto | Pediatric | 15C | GPSC11 | 51.54 | 2,101,472 | 54 | 86,936 | SAMN48929507 |
| NSPN0395 | TIBDN | Toronto | Pediatric | 19A | GPSC1 | 32.72 | 2,031,363 | 40 | 75,324 | SAMN48929465 |
| NSPN0420 | TIBDN | Toronto | Pediatric | 15B | GPSC11 | 33.03 | 2,099,234 | 48 | 91,396 | SAMN48929478 |
| NSPN0443 | TIBDN | Toronto | Pediatric | 19A | GPSC4 | 67.18 | 2,145,643 | 87 | 63,874 | SAMN48929498 |
| NSPN0463 | TIBDN | Toronto | Pediatric | 3 | GPSC12 | 51.6 | 1,981,074 | 36 | 111,021 | SAMN48929515 |
| NSPN0442 | TIBDN | Toronto | Pediatric | 3 | GPSC12 | 89.61 | 2,024,079 | 79 | 75,859 | SAMN48929497 |
| NSPN0439 | TIBDN | Toronto | Pediatric | 15B | GPSC25 | 54.67 | 2,073,832 | 86 | 53,740 | SAMN48929494 |
| NSPN0445 | TIBDN | Toronto | Pediatric | 7F | GPSC15 | 42.17 | 2,068,869 | 109 | 44,858 | SAMN48929500 |
| NSPN0397 | TIBDN | Toronto | Pediatric | 35B | GPSC75 | 51.88 | 2,070,347 | 101 | 51,072 | SAMN48929467 |
| NSPN0423 | TIBDN | Toronto | Pediatric | 38 | GPSC38 | 38.96 | 2,145,997 | 85 | 48,906 | SAMN48929480 |
| NSPN0424 | TIBDN | Toronto | Pediatric | 22F | GPSC19 | 465.35 | 2,115,154 | 65 | 75,351 | SAMN48929481 |
| NSPN0440 | TIBDN | Toronto | Pediatric | 23B | GPSC7 | 69.79 | 2,072,471 | 74 | 55,243 | SAMN48929495 |
| NSPN0398 | TIBDN | Toronto | Pediatric | 15B | GPSC11 | 49.97 | 2,121,470 | 125 | 30,435 | SAMN48929468 |
| NSPN0394 | TIBDN | Toronto | Pediatric | 15B | GPSC11 | 53.71 | 2,097,454 | 129 | 33,118 | SAMN48929464 |
| NSPN0436 | TIBDN | Toronto | Pediatric | NT | GPSC99 | 70.45 | 2,127,911 | 75 | 58,485 | SAMN48929491 |
| NSPN0406 | TIBDN | Toronto | Pediatric | 34 | GPSC45 | 50.49 | 2,112,595 | 69 | 84,400 | SAMN48929473 |
| NSPN0433 | TIBDN | Toronto | Pediatric | 15C | GPSC11 | 71.32 | 2,085,523 | 91 | 46,772 | SAMN48929488 |
| NSPN0407 | TIBDN | Toronto | Pediatric | 35B | GPSC75 | 53.85 | 2,070,420 | 104 | 49,734 | SAMN48929474 |
| NSPN0429 | TIBDN | Toronto | Pediatric | 19A | GPSC4 | 44.79 | 2,144,806 | 40 | 166,173 | SAMN48929485 |
| NSPN0427 | TIBDN | Toronto | Pediatric | 19F | GPSC1 | 37.3 | 2,053,366 | 37 | 129,574 | SAMN48929483 |
| NSPN0447 | TIBDN | Toronto | Pediatric | 15B | GPSC4 | 55.47 | 2,090,893 | 53 | 96,348 | SAMN48929502 |
| NSPN0418 | TIBDN | Toronto | Pediatric | 23A | GPSC11 | 70 | 2,101,521 | 79 | 78,006 | SAMN48929476 |
| NSPN0576 | TIBDN | Toronto | Pediatric | 11A | GPSC3 | 65.08 | 2,018,806 | 74 | 66,668 | SAMN48929598 |
| NSPN0393 | TIBDN | Toronto | Pediatric | 23A | GPSC5 | 50.9 | 2,103,728 | 61 | 69,532 | SAMN48929463 |
| NSPN0405 | TIBDN | Toronto | Pediatric | 15C | GPSC4 | 53.82 | 2,066,882 | 89 | 47,938 | SAMN48929472 |
| NSPN0492 | TIBDN | Toronto | Pediatric | 19A | GPSC27 | 53.02 | 2,107,903 | 100 | 39,436 | SAMN48929538 |
| NSPN0505 | TIBDN | Toronto | Pediatric | 31 | GPSC57 | 49.75 | 2,046,489 | 57 | 67,916 | SAMN48929548 |
| NSPN0493 | TIBDN | Toronto | Pediatric | 11A | GPSC3 | 54.51 | 2,013,069 | 89 | 62,811 | SAMN48929539 |
| NSPN0562 | TIBDN | Toronto | Pediatric | 15B | GPSC11 | 63.65 | 2,128,644 | 82 | 59,734 | SAMN48929589 |
| NSPN0508 | TIBDN | Toronto | Pediatric | 23B | GPSC7 | 54.9 | 2,069,769 | 104 | 39,503 | SAMN48929551 |
| NSPN0512 | TIBDN | Toronto | Pediatric | 19A | GPSC27 | 56.96 | 2,147,245 | 83 | 56,841 | SAMN48929554 |
| NSPN0515 | TIBDN | Toronto | Pediatric | 22F | GPSC19 | 39.81 | 2,036,071 | 79 | 55,117 | SAMN48929557 |
| NSPN0470 | TIBDN | Toronto | Pediatric | 6C | GPSC76 | 71.33 | 2,116,229 | 62 | 71,225 | SAMN48929522 |
| NSPN0469 | TIBDN | Toronto | Pediatric | 15B | GPSC11 | 53.86 | 2,173,338 | 83 | 54,107 | SAMN48929521 |
| NSPN0516 | TIBDN | Toronto | Pediatric | 19A | GPSC27 | 411.94 | 2,136,763 | 90 | 54,177 | SAMN48929558 |
| NSPN0481 | TIBDN | Toronto | Pediatric | 9V | GPSC43 | 55.64 | 2,013,899 | 75 | 51,398 | SAMN48929528 |
| NSPN0486 | TIBDN | Toronto | Pediatric | 9N | GPSC16 | 51.81 | 2,123,226 | 97 | 39,479 | SAMN48929532 |
| NSPN0500 | TIBDN | Toronto | Pediatric | 15A | GPSC11 | 49.03 | 2,122,994 | 117 | 39,365 | SAMN48929543 |
| NSPN0452 | TIBDN | Toronto | Pediatric | 10A | GPSC35 | 62.86 | 2,059,035 | 58 | 65,132 | SAMN48929506 |
| NSPN0471 | TIBDN | Toronto | Pediatric | 19A | GPSC27 | 71.98 | 2,183,616 | 99 | 55,824 | SAMN48929523 |
| NSPN0517 | TIBDN | Toronto | Pediatric | 15A | GPSC9 | 49.44 | 2,072,229 | 67 | 57,609 | SAMN48929559 |
| NSPN0506 | TIBDN | Toronto | Pediatric | 19A | GPSC27 | 57.96 | 2,146,426 | 145 | 35,116 | SAMN48929549 |
| NSPN0487 | TIBDN | Toronto | Pediatric | 28A | GPSC191 | 46.64 | 2,041,957 | 40 | 97,343 | SAMN48929533 |
| NSPN0490 | TIBDN | Toronto | Pediatric | 11A | GPSC642 | 43.94 | 2,107,570 | 158 | 36,453 | SAMN48929536 |
| NSPN0457 | TIBDN | Toronto | Pediatric | NT | GPSC3 | 51.8 | 2,028,539 | 57 | 70,872 | SAMN48929509 |
| NSPN0482 | TIBDN | Toronto | Pediatric | 3 | GPSC12 | 45.81 | 2,022,238 | 53 | 76,119 | SAMN48929529 |
| NSPN0513 | TIBDN | Toronto | Pediatric | 22F | GPSC19 | 40.38 | 2,035,408 | 56 | 77,609 | SAMN48929555 |
| NSPN0478 | TIBDN | Toronto | Pediatric | 19A | GPSC27 | 56.77 | 2,169,377 | 129 | 34,472 | SAMN48929526 |
| NSPN0451 | TIBDN | Toronto | Pediatric | 19A | GPSC1 | 66.93 | 2,053,343 | 62 | 57,240 | SAMN48929505 |
| NSPN0502 | TIBDN | Toronto | Pediatric | 15C | GPSC11 | 55.4 | 2,102,490 | 150 | 28,359 | SAMN48929545 |
| NSPN0460 | TIBDN | Toronto | Pediatric | 19A | GPSC27 | 55.69 | 2,149,216 | 104 | 43,945 | SAMN48929512 |
| NSPN0507 | TIBDN | Toronto | Pediatric | 19A | GPSC1 | 63.98 | 2,055,391 | 88 | 45,929 | SAMN48929550 |
| NSPN0488 | TIBDN | Toronto | Pediatric | 3 | GPSC12 | 37.84 | 2,017,278 | 63 | 73,588 | SAMN48929534 |
| NSPN0563 | TIBDN | Toronto | Pediatric | 23B | GPSC7 | 69.84 | 2,068,278 | 73 | 59,271 | SAMN48929590 |
| NSPN0489 | TIBDN | Toronto | Pediatric | 19A | GPSC27 | 66.63 | 2,158,071 | 111 | 47,480 | SAMN48929535 |
| NSPN0514 | TIBDN | Toronto | Pediatric | 19A | GPSC27 | 51.2 | 2,146,583 | 135 | 30,525 | SAMN48929556 |
| NSPN0496 | TIBDN | Toronto | Pediatric | 19A | GPSC27 | 52.84 | 2,148,695 | 97 | 47,299 | SAMN48929541 |
| NSPN0449 | TIBDN | Toronto | Pediatric | 19A | GPSC1 | 62.04 | 2,056,418 | 62 | 68,132 | SAMN48929503 |
| NSPN0450 | TIBDN | Toronto | Pediatric | 15A | GPSC6 | 61.6 | 2,089,189 | 76 | 61,259 | SAMN48929504 |
| NSPN0509 | TIBDN | Toronto | Pediatric | 22F | GPSC19 | 56.52 | 2,080,727 | 140 | 29,709 | SAMN48929552 |
| NSPN0494 | TIBDN | Toronto | Pediatric | 16F | GPSC156 | 62.3 | 2,069,937 | 104 | 60,274 | SAMN48929540 |
| NSPN0462 | TIBDN | Toronto | Pediatric | 22F | GPSC19 | 70.65 | 2,116,071 | 102 | 42,479 | SAMN48929514 |
| NSPN0479 | TIBDN | Toronto | Pediatric | 19A | GPSC4 | 64.13 | 2,184,194 | 107 | 46,503 | SAMN48929527 |
| NSPN0467 | TIBDN | Toronto | Pediatric | 15C | GPSC4 | 66.01 | 2,063,228 | 63 | 57,348 | SAMN48929519 |
| NSPN0501 | TIBDN | Toronto | Pediatric | 17F | GPSC49 | 62.27 | 2,089,687 | 92 | 51,391 | SAMN48929544 |
| NSPN0456 | TIBDN | Toronto | Pediatric | 38 | GPSC38 | 62.98 | 2,164,383 | 86 | 50,910 | SAMN48929508 |
| NSPN0459 | TIBDN | Toronto | Pediatric | 3 | GPSC12 | 69.96 | 2,024,961 | 53 | 84,737 | SAMN48929511 |
| NSPN0483 | TIBDN | Toronto | Pediatric | 23B | GPSC7 | 63.96 | 2,070,742 | 104 | 53,322 | SAMN48929530 |
| NSPN0461 | TIBDN | Toronto | Pediatric | 19A | GPSC5 | 50.57 | 2,113,942 | 97 | 52,390 | SAMN48929513 |
| NSPN0491 | TIBDN | Toronto | Pediatric | 11A | GPSC3 | 44.46 | 2,031,191 | 68 | 69,743 | SAMN48929537 |
| NSPN0504 | TIBDN | Toronto | Pediatric | 23B | GPSC7 | 48.65 | 2,071,430 | 60 | 89,134 | SAMN48929547 |
| NSPN0473 | TIBDN | Toronto | Pediatric | 33A | GPSC3 | 57.54 | 2,061,542 | 31 | 146,767 | SAMN48929525 |
| NSPN0458 | TIBDN | Toronto | Pediatric | 15B | GPSC11 | 42.33 | 2,105,896 | 35 | 127,957 | SAMN48929510 |
| NSPN0503 | TIBDN | Toronto | Pediatric | 21 | GPSC4 | 44.48 | 2,090,712 | 55 | 92,400 | SAMN48929546 |
| NSPN0472 | TIBDN | Toronto | Pediatric | 10A | GPSC36 | 63.88 | 2,070,607 | 49 | 82,818 | SAMN48929524 |
| NSPN0484 | TIBDN | Toronto | Pediatric | 3 | GPSC12 | 50.49 | 1,973,310 | 44 | 69,801 | SAMN48929531 |
| NSPN0468 | TIBDN | Toronto | Pediatric | 15C | GPSC4 | 74.08 | 2,156,608 | 120 | 38,695 | SAMN48929520 |
| NSPN0499 | TIBDN | Toronto | Pediatric | 23A | GPSC5 | 51.64 | 2,097,966 | 122 | 40,505 | SAMN48929542 |
| NSPN0510 | TIBDN | Toronto | Pediatric | 15A | GPSC6 | 55.66 | 2,087,952 | 98 | 49,855 | SAMN48929553 |
| NSPN0561 | TIBDN | Toronto | Pediatric | 8 | GPSC98 | 72.31 | 1,989,782 | 81 | 48,428 | SAMN48929588 |
| NSPN0523 | TIBDN | Toronto | Pediatric | 15B | GPSC11 | 36.27 | 2,156,580 | 96 | 46,730 | SAMN48929564 |
| NSPN0542 | TIBDN | Toronto | Pediatric | 22F | GPSC19 | 62.11 | 2,066,083 | 124 | 31,684 | SAMN48929576 |
| NSPN0524 | TIBDN | Toronto | Pediatric | 19A | GPSC1 | 49.39 | 2,080,251 | 85 | 71,277 | SAMN48929565 |
| NSPN0525 | TIBDN | Toronto | Pediatric | 19A | GPSC27 | 54.11 | 2,176,373 | 145 | 36,387 | SAMN48929566 |
| NSPN0520 | TIBDN | Toronto | Pediatric | 22F | GPSC19 | 49.67 | 2,104,390 | 95 | 35,272 | SAMN48929561 |
| NSPN0557 | TIBDN | Toronto | Pediatric | 22F | GPSC19 | 61.5 | 2,074,743 | 94 | 44,970 | SAMN48929584 |
| NSPN0540 | TIBDN | Toronto | Pediatric | 3 | GPSC12 | 63.55 | 1,985,268 | 74 | 58,843 | SAMN48929574 |
| NSPN0601 | TIBDN | Toronto | Pediatric | 19A | GPSC27 | 55.18 | 2,107,370 | 138 | 33,544 | SAMN48929617 |
| NSPN0539 | TIBDN | Toronto | Pediatric | 19A | GPSC27 | 62.91 | 2,136,268 | 121 | 40,232 | SAMN48929573 |
| NSPN0559 | TIBDN | Toronto | Pediatric | 22F | GPSC19 | 49.63 | 2,101,074 | 90 | 43,136 | SAMN48929586 |
| NSPN0571 | TIBDN | Toronto | Pediatric | 3 | GPSC51 | 58.14 | 2,031,322 | 132 | 34,724 | SAMN48929595 |
| NSPN0599 | TIBDN | Toronto | Pediatric | 19A | GPSC27 | 70.41 | 2,176,375 | 178 | 29,683 | SAMN48929615 |
| NSPN0544 | TIBDN | Toronto | Pediatric | 8 | GPSC3 | 87.25 | 1,986,346 | 27 | 131,405 | SAMN48929578 |
| NSPN0556 | TIBDN | Toronto | Pediatric | 24 | GPSC44 | 45.9 | 2,135,804 | 122 | 38,996 | SAMN48929583 |
| NSPN0532 | TIBDN | Toronto | Pediatric | 3 | GPSC51 | 61.19 | 2,028,440 | 79 | 55,753 | SAMN48929572 |
| NSPN0560 | TIBDN | Toronto | Pediatric | 15B | GPSC11 | 47.69 | 2,100,317 | 75 | 65,056 | SAMN48929587 |
| NSPN0545 | TIBDN | Toronto | Pediatric | 35F | GPSC45 | 59.5 | 2,114,750 | 76 | 56,831 | SAMN48929579 |
| NSPN0558 | TIBDN | Toronto | Pediatric | 7F | GPSC15 | 64.61 | 1,996,473 | 111 | 36,918 | SAMN48929585 |
| NSPN0528 | TIBDN | Toronto | Pediatric | 19A | GPSC4 | 500.2 | 2,144,566 | 66 | 80,434 | SAMN48929569 |
| NSPN0526 | TIBDN | Toronto | Pediatric | 23B | GPSC7 | 54.02 | 2,110,724 | 113 | 42,848 | SAMN48929567 |
| NSPN0519 | TIBDN | Toronto | Pediatric | 15C | GPSC11 | 45.16 | 2,087,398 | 125 | 37,685 | SAMN48929560 |
| NSPN0522 | TIBDN | Toronto | Pediatric | 19A | GPSC27 | 47.6 | 2,146,828 | 129 | 38,777 | SAMN48929563 |
| NSPN0564 | TIBDN | Toronto | Pediatric | 15B | GPSC11 | 69.42 | 2,100,489 | 109 | 54,704 | SAMN48929591 |
| NSPN0527 | TIBDN | Toronto | Pediatric | 3 | GPSC12 | 48.85 | 1,981,002 | 77 | 46,489 | SAMN48929568 |
| NSPN0602 | TIBDN | Toronto | Pediatric | 35B | GPSC59 | 64.25 | 2,012,632 | 84 | 45,853 | SAMN48929618 |
| NSPN0529 | TIBDN | Toronto | Pediatric | 9N | GPSC16 | 72.37 | 2,054,658 | 66 | 54,613 | SAMN48929570 |
| NSPN0566 | TIBDN | Toronto | Pediatric | 22F | GPSC19 | 55.53 | 2,036,423 | 62 | 93,025 | SAMN48929593 |
| NSPN0543 | TIBDN | Toronto | Pediatric | 3 | GPSC371 | 67.54 | 2,007,378 | 68 | 45,692 | SAMN48929577 |
| NSPN0555 | TIBDN | Toronto | Pediatric | 15B | GPSC11 | 64.53 | 2,089,335 | 59 | 87,904 | SAMN48929582 |
| NSPN0531 | TIBDN | Toronto | Pediatric | 15C | GPSC11 | 76.34 | 2,092,191 | 81 | 66,300 | SAMN48929571 |
| NSPN0632 | TIBDN | Toronto | Pediatric | 15C | GPSC4 | 58.12 | 2,071,692 | 95 | 49,034 | SAMN48929644 |
| NSPN0567 | TIBDN | Toronto | Pediatric | 23A | GPSC7 | 69.83 | 2,057,875 | 50 | 75,547 | SAMN48929594 |
| NSPN0521 | TIBDN | Toronto | Pediatric | 23A | GPSC7 | 47.22 | 2,061,046 | 124 | 32,783 | SAMN48929562 |
| NSPN0631 | TIBDN | Toronto | Pediatric | 22F | GPSC19 | 63.09 | 2,115,214 | 124 | 37,860 | SAMN48929643 |
| NSPN0554 | TIBDN | Toronto | Pediatric | 23A | GPSC5 | 64.46 | 2,077,290 | 115 | 40,885 | SAMN48929581 |
| NSPN0600 | TIBDN | Toronto | Pediatric | 23B | GPSC5 | 56.18 | 2,137,920 | 151 | 32,485 | SAMN48929616 |
| NSPN0553 | TIBDN | Toronto | Pediatric | 15C | GPSC11 | 43.97 | 2,084,159 | 78 | 67,558 | SAMN48929580 |
| NSPN0565 | TIBDN | Toronto | Pediatric | 38 | GPSC38 | 68.51 | 2,163,509 | 118 | 44,303 | SAMN48929592 |
| NSPN0633 | TIBDN | Toronto | Pediatric | 9N | GPSC16 | 58.83 | 2,050,753 | 64 | 69,566 | SAMN48929645 |
| NSPN0541 | TIBDN | Toronto | Pediatric | 35B | GPSC59 | 59.29 | 2,010,176 | 41 | 89,032 | SAMN48929575 |
| NSPN0597 | TIBDN | Toronto | Pediatric | 9N | GPSC16 | 75.27 | 2,046,395 | 101 | 42,971 | SAMN48929613 |
| NSPN0622 | TIBDN | Toronto | Pediatric | 19A | GPSC27 | 62.84 | 2,106,990 | 140 | 34,419 | SAMN48929635 |
| NSPN0616 | TIBDN | Toronto | Pediatric | 38 | GPSC38 | 53.56 | 2,114,182 | 154 | 32,820 | SAMN48929630 |
| NSPN0584 | TIBDN | Toronto | Pediatric | 15C | GPSC11 | 51.42 | 2,172,163 | 112 | 42,720 | SAMN48929604 |
| NSPN0607 | TIBDN | Toronto | Pediatric | 22F | GPSC19 | 85.22 | 2,121,057 | 50 | 75,876 | SAMN48929623 |
| NSPN0627 | TIBDN | Toronto | Pediatric | 7C | GPSC44 | 56.04 | 2,122,857 | 158 | 27,127 | SAMN48929639 |
| NSPN0611 | TIBDN | Toronto | Pediatric | 10A | GPSC10 | 63.89 | 2,075,590 | 75 | 69,799 | SAMN48929625 |
| NSPN0623 | TIBDN | Toronto | Pediatric | 9N | GPSC16 | 53.39 | 2,130,219 | 176 | 27,644 | SAMN48929636 |
| NSPN0593 | TIBDN | Toronto | Pediatric | 31 | GPSC10 | 71.55 | 2,088,317 | 124 | 43,313 | SAMN48929609 |
| NSPN0617 | TIBDN | Toronto | Pediatric | 23A | GPSC5 | 53.56 | 2,073,644 | 74 | 61,588 | SAMN48929631 |
| NSPN0604 | TIBDN | Toronto | Pediatric | 15C | GPSC11 | 63.82 | 2,137,335 | 89 | 58,970 | SAMN48929620 |
| NSPN0583 | TIBDN | Toronto | Pediatric | 22F | GPSC19 | 51.35 | 2,100,202 | 51 | 65,375 | SAMN48929603 |
| NSPN0598 | TIBDN | Toronto | Pediatric | 3 | GPSC12 | 63.96 | 1,974,027 | 82 | 42,964 | SAMN48929614 |
| NSPN0603 | TIBDN | Toronto | Pediatric | 10A | GPSC36 | 60.29 | 2,068,275 | 83 | 64,990 | SAMN48929619 |
| NSPN0624 | TIBDN | Toronto | Pediatric | 38 | GPSC38 | 58.62 | 2,169,574 | 148 | 28,094 | SAMN48929637 |
| NSPN0629 | TIBDN | Toronto | Pediatric | 15C | GPSC11 | 55.33 | 2,090,906 | 116 | 35,623 | SAMN48929641 |
| NSPN0630 | TIBDN | Toronto | Pediatric | 15A | GPSC6 | 39.57 | 2,090,659 | 125 | 32,355 | SAMN48929642 |
| NSPN0618 | TIBDN | Toronto | Pediatric | 15B | GPSC11 | 47.76 | 2,101,232 | 128 | 47,652 | SAMN48929632 |
| NSPN0594 | TIBDN | Toronto | Pediatric | 33A | GPSC3 | 53.05 | 2,058,842 | 117 | 42,495 | SAMN48929610 |
| NSPN0590 | TIBDN | Toronto | Pediatric | 19A | GPSC27 | 65.12 | 2,148,491 | 101 | 42,836 | SAMN48929606 |
| NSPN0625 | TIBDN | Toronto | Pediatric | 3 | GPSC12 | 59.5 | 2,010,258 | 63 | 65,193 | SAMN48929638 |
| NSPN0628 | TIBDN | Toronto | Pediatric | 35F | GPSC36 | 50.54 | 2,097,679 | 107 | 49,075 | SAMN48929640 |
| NSPN0595 | TIBDN | Toronto | Pediatric | 3 | GPSC12 | 43.25 | 1,986,665 | 56 | 62,351 | SAMN48929611 |
| NSPN0605 | TIBDN | Toronto | Pediatric | 23B | GPSC7 | 45.09 | 2,120,339 | 115 | 39,699 | SAMN48929621 |
| NSPN0592 | TIBDN | Toronto | Pediatric | 3 | GPSC12 | 54.69 | 2,021,908 | 85 | 47,878 | SAMN48929608 |
| NSPN0621 | TIBDN | Toronto | Pediatric | 15C | GPSC11 | 64.75 | 2,155,166 | 123 | 38,123 | SAMN48929634 |
| NSPN0614 | TIBDN | Toronto | Pediatric | 15B | GPSC10 | 74.52 | 2,084,507 | 66 | 60,902 | SAMN48929628 |
| NSPN0612 | TIBDN | Toronto | Pediatric | 15C | GPSC4 | 63.21 | 2,106,143 | 119 | 38,637 | SAMN48929626 |
| NSPN0615 | TIBDN | Toronto | Pediatric | 15C | GPSC11 | 63.28 | 2,140,215 | 114 | 43,230 | SAMN48929629 |
| NSPN0613 | TIBDN | Toronto | Pediatric | 8 | GPSC3 | 50.97 | 1,983,096 | 44 | 81,681 | SAMN48929627 |
| NSPN0606 | TIBDN | Toronto | Pediatric | 15C | GPSC11 | 47.31 | 2,094,021 | 143 | 39,620 | SAMN48929622 |
| NSPN0608 | TIBDN | Toronto | Pediatric | 22F | GPSC19 | 60.79 | 2,077,382 | 66 | 50,408 | SAMN48929624 |
| NSPN0620 | TIBDN | Toronto | Pediatric | 35A | GPSC6 | 45.67 | 2,069,921 | 93 | 49,649 | SAMN48929633 |
| NSPN0589 | TIBDN | Toronto | Pediatric | 19A | GPSC27 | 46.56 | 2,186,975 | 129 | 34,523 | SAMN48929605 |
| NSPN0596 | TIBDN | Toronto | Pediatric | 38 | GPSC38 | 57.74 | 2,164,194 | 176 | 27,416 | SAMN48929612 |
| NSPN0591 | TIBDN | Toronto | Pediatric | 15B | GPSC11 | 52.97 | 2,094,866 | 83 | 56,925 | SAMN48929607 |
| SP154 | PHO | Ontario | Adult | 8 | GPSC98 | 483.46 | 2,043,965 | 51 | 91,895 | SRR3211722 |
| SP183 | PHO | Ontario | Adult | 8 | GPSC3 | 197.97 | 2,076,504 | 69 | 71,674 | SRR3211725 |
| SP186 | PHO | Ontario | Adult | 8 | GPSC98 | 104.28 | 1,992,682 | 61 | 78,500 | SRR3211726 |
| SP187 | PHO | Ontario | Adult | 34 | GPSC45 | 91.25 | 2,111,213 | 67 | 74,787 | SRR5011737 |
| SP165 | PHO | Ontario | Adult | 11A | GPSC3 | 144.75 | 2,017,049 | 54 | 65,011 | SRR5011704 |
| SP162 | PHO | Ontario | Adult | 11A | GPSC3 | 377.29 | 2,017,586 | 47 | 75,935 | SRR5011807 |
| SP166 | PHO | Ontario | Adult | 11A | GPSC3 | 271.13 | 2,013,690 | 43 | 93,603 | SRR5011721 |
| SP155 | PHO | Ontario | Adult | 11A | GPSC3 | 420.38 | 2,054,828 | 56 | 72,272 | SRR5011738 |
| SP179 | PHO | Ontario | Adult | 12F | GPSC32 | 176.11 | 1,988,889 | 103 | 47,678 | SRR5011822 |
| SP160 | PHO | Ontario | Adult | 12F | GPSC32 | 53.81 | 1,988,050 | 75 | 50,394 | SRR5011715 |
| SP178 | PHO | Ontario | Adult | 15A | GPSC9 | 187.33 | 2,072,695 | 84 | 56,464 | SRR5011717 |
| SP192 | PHO | Ontario | Adult | 15A | GPSC9 | 151.92 | 2,074,584 | 57 | 73,631 | SRR3211693 |
| SP161 | PHO | Ontario | Adult | 15B | GPSC11 | 275.71 | 2,106,674 | 67 | 76,623 | SRR5011781 |
| SP189 | PHO | Ontario | Adult | 16F | GPSC156 | 269.05 | 2,119,183 | 41 | 93,617 | SRR5011680 |
| SP188 | PHO | Ontario | Adult | 22F | GPSC19 | 173.55 | 2,079,884 | 108 | 44,068 | SRR3211718 |
| SP174 | PHO | Ontario | Adult | 22F | GPSC19 | 266.91 | 2,066,593 | 74 | 64,945 | SRR3211716 |
| SP168 | PHO | Ontario | Adult | 22F | GPSC19 | 182.74 | 2,034,494 | 82 | 56,311 | SRR3211715 |
| SP185 | PHO | Ontario | Adult | 22F | GPSC19 | 247.74 | 2,037,131 | 39 | 91,647 | SRR3211717 |
| SP184 | PHO | Ontario | Adult | 23A | GPSC7 | 163.37 | 2,058,020 | 52 | 101,168 | SRR5011810 |
| SP163 | PHO | Ontario | Adult | 23A | GPSC5 | 127.74 | 2,058,770 | 68 | 70,606 | SRR5011763 |
| SP190 | PHO | Ontario | Adult | 33F | GPSC3 | 56.76 | 2,055,380 | 24 | 171,094 | SRR5011739 |
| SP167 | PHO | Ontario | Adult | 33F | GPSC3 | 106.21 | 2,061,298 | 41 | 90,820 | SRR5011669 |
| SP173 | PHO | Ontario | Adult | 35B | GPSC75 | 216.28 | 2,075,517 | 96 | 51,162 | SRR5011768 |
| SP176 | PHO | Ontario | Adult | 9N | GPSC32 | 55.07 | 2,004,809 | 53 | 72,213 | SRR5011806 |
| SP169 | PHO | Ontario | Adult | 9N | GPSC16 | 308.29 | 2,092,519 | 48 | 79,184 | SRR5011686 |
| SP191 | PHO | Ontario | Adult | 7F | GPSC15 | 183.89 | 2,002,399 | 68 | 60,915 | SRR5011755 |
| SP158 | PHO | Ontario | Adult | 7F | GPSC15 | 276.56 | 1,994,288 | 58 | 69,501 | SRR5011826 |
| SP181 | PHO | Ontario | Adult | 3 | GPSC12 | 158.55 | 2,026,619 | 57 | 92,074 | SRR5011720 |
| SP172 | PHO | Ontario | Adult | 3 | GPSC12 | 250.17 | 2,037,076 | 70 | 85,670 | SRR5011723 |
| SP180 | PHO | Ontario | Adult | 19A | GPSC4 | 172.31 | 2,138,339 | 104 | 48,522 | SRR5011767 |
| SP164 | PHO | Ontario | Adult | 19A | GPSC4 | 301.27 | 2,129,330 | 53 | 108,168 | SRR5011745 |
| SP157 | PHO | Ontario | Adult | 6A | GPSC13 | 231.08 | 2,073,605 | 87 | 59,421 | SRR5011817 |
| SP170 | PHO | Ontario | Adult | 6A | GPSC111 | 220 | 2,071,986 | 81 | 68,390 | SRR5011685 |
| SP177 | PHO | Ontario | Adult | 19F | GPSC119 | 122.77 | 2,165,598 | 104 | 57,589 | SRR5011753 |
| SP156 | PHO | Ontario | Adult | 19F | GPSC11 | 262.56 | 2,092,285 | 78 | 82,407 | SRR5011711 |
| SP175 | PHO | Ontario | Adult | 23F | GPSC7 | 250.02 | 2,077,987 | 50 | 84,799 | SRR5011727 |
| SP171 | PHO | Ontario | Adult | 9V | GPSC6 | 315.3 | 2,136,096 | 55 | 82,062 | SRR5011678 |
| SP159 | PHO | Ontario | Adult | 9V | GPSC6 | 172.18 | 2,111,658 | 58 | 78,549 | SRR5011663 |
| SP122 | PHO | Ontario | Adult | 8 | GPSC3 | 311.93 | 1,985,249 | 83 | 68,667 | SRR3211711 |
| SP137 | PHO | Ontario | Adult | 10A | GPSC36 | 195.24 | 2,064,985 | 57 | 78,289 | SRR5011829 |
| SP119 | PHO | Ontario | Adult | 11A | GPSC3 | 81.4 | 2,057,223 | 39 | 88,572 | SRR5011703 |
| SP112 | PHO | Ontario | Adult | 12F | GPSC32 | 41.23 | 1,989,659 | 53 | 77,290 | SRR5011709 |
| SP123 | PHO | Ontario | Adult | 12F | GPSC32 | 265.02 | 1,987,332 | 112 | 47,400 | SRR5011676 |
| SP98 | PHO | Ontario | Adult | 15A | GPSC9 | 191.65 | 2,073,505 | 57 | 75,175 | SRR3211691 |
| SP117 | PHO | Ontario | Adult | 15A | GPSC9 | 118.59 | 2,091,529 | 62 | 74,086 | SRR3211692 |
| SP138 | PHO | Ontario | Adult | 15B | GPSC11 | 376.82 | 2,092,529 | 45 | 116,752 | SRR5011690 |
| SP111 | PHO | Ontario | Adult | 16F | GPSC156 | 141.18 | 2,077,184 | 54 | 86,399 | SRR5011801 |
| SP109 | PHO | Ontario | Adult | 22F | GPSC19 | 189.79 | 2,080,797 | 112 | 46,002 | SRR3211709 |
| SP136 | PHO | Ontario | Adult | 22F | GPSC19 | 106.56 | 2,072,197 | 63 | 55,638 | SRR3211713 |
| SP100 | PHO | Ontario | Adult | 22F | GPSC19 | 79.09 | 2,118,019 | 107 | 45,743 | SRR3211708 |
| SP143 | PHO | Ontario | Adult | 22F | GPSC19 | 287.46 | 2,111,749 | 46 | 99,705 | SRR3211714 |
| SP128 | PHO | Ontario | Adult | 23A | GPSC5 | 187.09 | 2,119,879 | 41 | 97,537 | SRR5011809 |
| SP142 | PHO | Ontario | Adult | 23B | GPSC7 | 204.35 | 2,078,516 | 115 | 47,797 | SRR5011784 |
| SP129 | PHO | Ontario | Adult | 33F | GPSC3 | 126.03 | 2,068,788 | 61 | 72,253 | SRR5011793 |
| SP120 | PHO | Ontario | Adult | 35B | GPSC59 | 79.42 | 2,011,965 | 32 | 116,728 | SRR5011683 |
| SP121 | PHO | Ontario | Adult | 35F | GPSC36 | 409.19 | 2,059,411 | 114 | 46,922 | SRR5011832 |
| SP105 | PHO | Ontario | Adult | 9N | GPSC16 | 223.26 | 2,047,878 | 74 | 58,708 | SRR5011811 |
| SP135 | PHO | Ontario | Adult | 9N | GPSC16 | 113.02 | 2,048,884 | 49 | 81,572 | SRR5011710 |
| SP102 | PHO | Ontario | Adult | 7F | GPSC15 | 172.74 | 1,998,106 | 85 | 56,203 | SRR5011796 |
| SP133 | PHO | Ontario | Adult | 7F | GPSC15 | 106.43 | 1,998,354 | 73 | 58,886 | SRR5011830 |
| SP134 | PHO | Ontario | Adult | 7F | GPSC15 | 158.34 | 1,995,099 | 82 | 59,330 | SRR5011708 |
| SP132 | PHO | Ontario | Adult | 7F | GPSC15 | 189.9 | 1,994,039 | 68 | 65,446 | SRR5011758 |
| SP127 | PHO | Ontario | Adult | 7F | GPSC15 | 120.52 | 1,994,445 | 112 | 40,779 | SRR5011779 |
| SP108 | PHO | Ontario | Adult | 7F | GPSC15 | 224.53 | 2,012,510 | 115 | 57,914 | SRR5011841 |
| SP116 | PHO | Ontario | Adult | 7F | GPSC15 | 217.74 | 1,997,709 | 79 | 63,469 | SRR5011700 |
| SP101 | PHO | Ontario | Adult | 3 | GPSC12 | 112.26 | 2,024,246 | 54 | 97,196 | SRR5011823 |
| SP118 | PHO | Ontario | Adult | 3 | GPSC12 | 137.23 | 1,975,859 | 43 | 89,370 | SRR5011750 |
| SP130 | PHO | Ontario | Adult | 3 | GPSC12 | 350.75 | 1,991,711 | 111 | 72,327 | SRR5011785 |
| SP107 | PHO | Ontario | Adult | 19A | GPSC4 | 159.5 | 2,218,856 | 68 | 73,452 | SRR5011688 |
| SP144 | PHO | Ontario | Adult | 19A | GPSC27 | 206.96 | 2,149,532 | 62 | 72,015 | SRR5011795 |
| SP104 | PHO | Ontario | Adult | 19A | GPSC27 | 224.48 | 2,112,017 | 36 | 88,891 | SRR5011696 |
| SP131 | PHO | Ontario | Adult | 19A | GPSC4 | 131.41 | 2,070,552 | 66 | 86,395 | SRR5011803 |
| SP113 | PHO | Ontario | Adult | 19A | GPSC4 | 101.49 | 2,068,590 | 62 | 69,118 | SRR5011673 |
| SP139 | PHO | Ontario | Adult | 19A | GPSC4 | 229.04 | 2,114,243 | 79 | 62,686 | SRR5011756 |
| SP110 | PHO | Ontario | Adult | 6A | GPSC13 | 198.17 | 2,073,253 | 55 | 81,014 | SRR5011821 |
| SP99 | PHO | Ontario | Adult | 6A | GPSC13 | 93.4 | 2,070,030 | 57 | 79,169 | SRR5011792 |
| SP140 | PHO | Ontario | Adult | 4 | GPSC27 | 345.52 | 2,113,073 | 78 | 55,923 | SRR5011692 |
| SP106 | PHO | Ontario | Adult | 14 | GPSC18 | 262.92 | 2,073,488 | 106 | 49,173 | SRR5011657 |
| SP124 | PHO | Ontario | Adult | 18C | GPSC3 | 339.57 | 2,126,568 | 55 | 117,486 | SRR5011730 |
| SP114 | PHO | Ontario | Adult | 19F | GPSC119 | 207.49 | 2,089,126 | 109 | 47,772 | SRR5011844 |
| SP125 | PHO | Ontario | Adult | 23F | GPSC7 | 168.23 | 2,065,344 | 68 | 84,817 | SRR5011774 |
| SP97 | PHO | Ontario | Adult | 9V | GPSC6 | 191.81 | 2,116,610 | 68 | 73,358 | SRR5011733 |
| SP103 | PHO | Ontario | Adult | 23A | GPSC7 | 135.21 | 2,048,513 | 35 | 106,539 | SRR5011681 |
| SP141 | PHO | Ontario | Adult | 9N | GPSC16 | 228.31 | 2,054,905 | 69 | 58,316 | SRR5011748 |
| SP115 | PHO | Ontario | Adult | 19A | GPSC4 | 179.03 | 2,147,883 | 95 | 49,943 | SRR5011741 |
| SP71 | PHO | Ontario | Adult | 8 | GPSC98 | 238.33 | 1,990,180 | 53 | 85,514 | SRR3211699 |
| SP67 | PHO | Ontario | Adult | 8 | GPSC98 | 74.28 | 1,989,714 | 41 | 97,752 | SRR3211688 |
| SP60 | PHO | Ontario | Adult | 29 | GPSC75 | 246.85 | 2,054,559 | 89 | 52,562 | SRR5011665 |
| SP77 | PHO | Ontario | Adult | 34 | GPSC45 | 419.07 | 2,113,411 | 46 | 113,856 | SRR5011746 |
| SP64 | PHO | Ontario | Adult | 34 | GPSC45 | 129.46 | 2,105,464 | 86 | 66,731 | SRR5011802 |
| SP83 | PHO | Ontario | Adult | 11A | GPSC3 | 41.99 | 2,049,456 | 44 | 71,722 | SRR5011705 |
| SP56 | PHO | Ontario | Adult | 12F | GPSC32 | 103.11 | 1,996,257 | 66 | 77,287 | SRR5011759 |
| SP55 | PHO | Ontario | Adult | 12F | GPSC32 | 166.95 | 2,002,990 | 76 | 54,825 | SRR5011716 |
| SP66 | PHO | Ontario | Adult | 15A | GPSC9 | 279.89 | 2,064,463 | 45 | 74,165 | SRR3211690 |
| SP74 | PHO | Ontario | Adult | 15B | GPSC11 | 95.67 | 2,081,366 | 60 | 75,299 | SRR5011740 |
| SP84 | PHO | Ontario | Adult | 17F | GPSC50 | 74.53 | 2,137,463 | 65 | 77,768 | SRR5011831 |
| SP80 | PHO | Ontario | Adult | 22F | GPSC19 | 61.06 | 2,070,958 | 67 | 69,228 | SRR3211707 |
| SP62 | PHO | Ontario | Adult | 22F | GPSC19 | 139.26 | 2,072,802 | 72 | 47,648 | SRR3211704 |
| SP76 | PHO | Ontario | Adult | 22F | GPSC19 | 198.96 | 2,031,127 | 48 | 89,332 | SRR3211705 |
| SP78 | PHO | Ontario | Adult | 22F | GPSC19 | 130.7 | 2,063,756 | 53 | 63,523 | SRR3211706 |
| SP72 | PHO | Ontario | Adult | 23A | GPSC7 | 151.69 | 2,078,016 | 54 | 76,432 | SRR5011744 |
| SP69 | PHO | Ontario | Adult | 33A | GPSC3 | 258.88 | 2,054,037 | 34 | 148,099 | SRR5011735 |
| SP54 | PHO | Ontario | Adult | 35B | GPSC75 | 258.37 | 2,086,570 | 76 | 73,386 | SRR5011786 |
| SP52 | PHO | Ontario | Adult | 35F | GPSC36 | 190.76 | 2,048,014 | 58 | 70,710 | SRR5011846 |
| SP94 | PHO | Ontario | Adult | 6C | GPSC29 | 358.9 | 2,085,782 | 61 | 74,519 | SRR5011761 |
| SP53 | PHO | Ontario | Adult | 7C | GPSC18 | 246.36 | 2,077,774 | 62 | 60,015 | SRR5011707 |
| SP91 | PHO | Ontario | Adult | 9N | GPSC16 | 328.53 | 2,041,054 | 48 | 95,163 | SRR5011743 |
| SP85 | PHO | Ontario | Adult | 9N | GPSC16 | 163.71 | 2,046,654 | 60 | 81,004 | SRR5011702 |
| SP59 | PHO | Ontario | Adult | 5 | GPSC8 | 312.99 | 2,082,899 | 89 | 66,432 | SRR5011834 |
| SP51 | PHO | Ontario | Adult | 7F | GPSC15 | 102.06 | 1,985,887 | 89 | 57,812 | SRR5011794 |
| SP63 | PHO | Ontario | Adult | 7F | GPSC15 | 252.95 | 1,989,152 | 96 | 59,587 | SRR5011697 |
| SP70 | PHO | Ontario | Adult | 7F | GPSC15 | 199.91 | 1,990,716 | 80 | 59,061 | SRR5011695 |
| SP95 | PHO | Ontario | Adult | 7F | GPSC15 | 224.78 | 1,991,681 | 50 | 71,287 | SRR5011804 |
| SP93 | PHO | Ontario | Adult | 7F | GPSC15 | 277.86 | 1,990,683 | 69 | 55,021 | SRR5011816 |
| SP75 | PHO | Ontario | Adult | 7F | GPSC15 | 144.79 | 1,991,064 | 80 | 50,180 | SRR5011667 |
| SP50 | PHO | Ontario | Adult | 3 | GPSC12 | 207.56 | 2,037,858 | 66 | 73,426 | SRR5011654 |
| SP61 | PHO | Ontario | Adult | 3 | GPSC12 | 408.06 | 2,016,884 | 38 | 187,058 | SRR5011845 |
| SP68 | PHO | Ontario | Adult | 19A | GPSC27 | 249.87 | 2,103,927 | 73 | 50,050 | SRR5011782 |
| SP92 | PHO | Ontario | Adult | 19A | GPSC27 | 279.82 | 2,102,296 | 46 | 121,452 | SRR5011749 |
| SP89 | PHO | Ontario | Adult | 19A | GPSC27 | 191.45 | 2,135,331 | 67 | 57,473 | SRR5011729 |
| SP96 | PHO | Ontario | Adult | 19A | GPSC4 | 108.49 | 2,051,790 | 61 | 73,401 | SRR5011797 |
| SP82 | PHO | Ontario | Adult | 4 | GPSC27 | 138.46 | 2,100,198 | 104 | 47,137 | SRR5011772 |
| SP90 | PHO | Ontario | Adult | 14 | GPSC4 | 85.62 | 2,085,131 | 57 | 58,600 | SRR5011724 |
| SP57 | PHO | Ontario | Adult | 18C | GPSC3 | 204.09 | 2,012,331 | 43 | 90,543 | SRR5011679 |
| SP58 | PHO | Ontario | Adult | 19F | GPSC119 | 317.59 | 2,096,503 | 94 | 46,408 | SRR5011731 |
| SP4 | PHO | Ontario | Adult | 8 | GPSC3 | 339.12 | 1,992,168 | 34 | 118,447 | SRR3211687 |
| SP5 | PHO | Ontario | Adult | 20 | GPSC124 | 370.68 | 2,080,376 | 32 | 159,450 | SRR5011780 |
| SP6 | PHO | Ontario | Adult | 34 | GPSC45 | 45.98 | 2,087,330 | 38 | 142,991 | SRR5011833 |
| SP27 | PHO | Ontario | Adult | 38 | GPSC38 | 308.69 | 2,164,021 | 64 | 86,995 | SRR5011656 |
| SP39 | PHO | Ontario | Adult | 11A | GPSC3 | 224.25 | 2,004,428 | 45 | 78,165 | SRR5011825 |
| SP37 | PHO | Ontario | Adult | 15A | GPSC9 | 125.59 | 2,064,481 | 55 | 84,338 | SRR3211689 |
| SP7 | PHO | Ontario | Adult | 15A | GPSC9 | 247.87 | 2,105,943 | 51 | 90,724 | SRR5011689 |
| SP35 | PHO | Ontario | Adult | 16F | GPSC135 | 174.55 | 2,052,371 | 56 | 80,849 | SRR5011828 |
| SP45 | PHO | Ontario | Adult | 16F | GPSC156 | 200.9 | 2,147,452 | 36 | 131,770 | SRR5011754 |
| SP17 | PHO | Ontario | Adult | 17F | GPSC50 | 153.65 | 2,136,262 | 42 | 101,125 | SRR5011677 |
| SP21 | PHO | Ontario | Adult | 22F | GPSC19 | 176.44 | 2,097,497 | 51 | 99,507 | SRR3211697 |
| SP18 | PHO | Ontario | Adult | 22F | GPSC19 | 158.11 | 2,130,335 | 52 | 77,494 | SRR3211696 |
| SP31 | PHO | Ontario | Adult | 22F | GPSC19 | 308.73 | 2,064,841 | 48 | 79,334 | SRR3211702 |
| SP30 | PHO | Ontario | Adult | 22F | GPSC19 | 345.28 | 2,042,180 | 48 | 77,862 | SRR3211701 |
| SP42 | PHO | Ontario | Adult | 22F | GPSC19 | 202.91 | 2,071,822 | 70 | 61,961 | SRR3211703 |
| SP26 | PHO | Ontario | Adult | 22F | GPSC19 | 204 | 2,064,526 | 42 | 79,342 | SRR3211698 |
| SP20 | PHO | Ontario | Adult | 23A | GPSC7 | 122.98 | 2,053,436 | 37 | 173,731 | SRR5011687 |
| SP24 | PHO | Ontario | Adult | 23A | GPSC7 | 111.13 | 2,053,047 | 40 | 92,672 | SRR5011706 |
| SP34 | PHO | Ontario | Adult | 23A | GPSC7 | 292.43 | 2,067,625 | 53 | 82,210 | SRR5011771 |
| SP15 | PHO | Ontario | Adult | 33F | GPSC3 | 193.01 | 2,052,472 | 36 | 84,545 | SRR5011670 |
| SP12 | PHO | Ontario | Adult | 35B | GPSC15 | 181.62 | 1,988,974 | 80 | 59,524 | SRR5011757 |
| SP48 | PHO | Ontario | Adult | 6C | GPSC76 | 326.32 | 2,124,044 | 44 | 78,328 | SRR5011712 |
| SP43 | PHO | Ontario | Adult | 9L | GPSC16 | 261.25 | 2,064,563 | 74 | 57,017 | SRR5011719 |
| SP44 | PHO | Ontario | Adult | 9N | GPSC16 | 237.28 | 2,091,755 | 63 | 73,977 | SRR5011734 |
| SP32 | PHO | Ontario | Adult | 9N | GPSC16 | 204.71 | 2,040,470 | 63 | 74,554 | SRR5011698 |
| SP13 | PHO | Ontario | Adult | 7F | GPSC15 | 240.85 | 2,062,748 | 74 | 60,360 | SRR5011662 |
| SP40 | PHO | Ontario | Adult | 7F | GPSC15 | 244.08 | 1,994,483 | 76 | 64,917 | SRR5011783 |
| SP28 | PHO | Ontario | Adult | 7F | GPSC15 | 302.74 | 1,992,241 | 48 | 86,777 | SRR5011726 |
| SP11 | PHO | Ontario | Adult | 7F | GPSC15 | 210.5 | 1,993,283 | 75 | 57,894 | SRR5011714 |
| SP1 | PHO | Ontario | Adult | 3 | GPSC12 | 214.62 | 2,020,186 | 37 | 97,525 | SRR5011732 |
| SP41 | PHO | Ontario | Adult | 3 | GPSC12 | 229.81 | 1,985,173 | 41 | 91,228 | SRR5011694 |
| SP2 | PHO | Ontario | Adult | 3 | GPSC12 | 114.8 | 2,017,846 | 24 | 277,984 | SRR5011808 |
| SP38 | PHO | Ontario | Adult | 3 | GPSC12 | 208.94 | 1,984,814 | 32 | 122,487 | SRR5011693 |
| SP23 | PHO | Ontario | Adult | 19A | GPSC4 | 190.35 | 2,069,614 | 32 | 175,906 | SRR5011836 |
| SP22 | PHO | Ontario | Adult | 19A | GPSC27 | 217.76 | 2,142,453 | 48 | 98,506 | SRR5011661 |
| SP8 | PHO | Ontario | Adult | 19A | GPSC18 | 188.65 | 2,085,377 | 45 | 106,601 | SRR5011815 |
| SP19 | PHO | Ontario | Adult | 19A | GPSC4 | 187.04 | 2,084,678 | 51 | 95,155 | SRR5011800 |
| SP9 | PHO | Ontario | Adult | 6A | GPSC13 | 99.82 | 2,067,113 | 65 | 63,381 | SRR5011840 |
| SP3 | PHO | Ontario | Adult | 4 | GPSC27 | 259.59 | 2,106,535 | 65 | 67,002 | SRR5011653 |
| SP16 | PHO | Ontario | Adult | 18C | GPSC3 | 171.24 | 2,085,703 | 22 | 152,786 | SRR5011789 |
| SP14 | PHO | Ontario | Adult | 6B | GPSC23 | 274.41 | 2,010,389 | 37 | 81,181 | SRR5011838 |
| SP25 | PHO | Ontario | Adult | 9V | GPSC6 | 194.13 | 2,076,227 | 53 | 84,269 | SRR5011777 |
| SP219 | PHO | Ontario | Adult | 8 | GPSC98 | 168.67 | 2,000,806 | 48 | 86,922 | SRR3211728 |
| SP239 | PHO | Ontario | Adult | 8 | GPSC98 | 142.77 | 1,994,186 | 29 | 123,275 | SRR3211729 |
| SP218 | PHO | Ontario | Adult | 31 | GPSC57 | 122.69 | 2,021,389 | 55 | 67,532 | SRR5011752 |
| SP220 | PHO | Ontario | Adult | 38 | GPSC38 | 46.38 | 2,129,594 | 68 | 56,926 | SRR5011664 |
| SP222 | PHO | Ontario | Adult | 10A | GPSC36 | 146.08 | 2,098,156 | 50 | 93,022 | SRR5011742 |
| SP201 | PHO | Ontario | Adult | 11A | GPSC4 | 106.22 | 2,149,440 | 37 | 127,509 | SRR5011701 |
| SP223 | PHO | Ontario | Adult | 15A | GPSC9 | 269.54 | 2,076,814 | 63 | 58,239 | SRR3211694 |
| SP230 | PHO | Ontario | Adult | 15A | GPSC9 | 193.27 | 2,076,226 | 45 | 74,310 | SRR3211695 |
| SP227 | PHO | Ontario | Adult | 15A | GPSC6 | 356.67 | 2,094,276 | 63 | 83,632 | SRR5011843 |
| SP202 | PHO | Ontario | Adult | 15B | GPSC6 | 110.82 | 2,132,675 | 46 | 82,090 | SRR5011728 |
| SP210 | PHO | Ontario | Adult | 22F | GPSC19 | 101.3 | 2,094,345 | 63 | 61,536 | SRR3211721 |
| SP215 | PHO | Ontario | Adult | 22F | GPSC19 | 98.41 | 2,081,696 | 65 | 70,015 | SRR3211723 |
| SP216 | PHO | Ontario | Adult | 22F | GPSC19 | 132.95 | 2,074,110 | 78 | 58,830 | SRR5011658 |
| SP226 | PHO | Ontario | Adult | 22F | GPSC19 | 301.82 | 2,074,799 | 76 | 59,621 | SRR3211724 |
| SP194 | PHO | Ontario | Adult | 22F | GPSC19 | 53.66 | 2,038,594 | 52 | 87,616 | SRR3211719 |
| SP203 | PHO | Ontario | Adult | 22F | GPSC19 | 90.1 | 2,078,868 | 41 | 90,203 | SRR3211720 |
| SP195 | PHO | Ontario | Adult | 23A | GPSC7 | 83.15 | 2,062,466 | 26 | 293,293 | SRR5011778 |
| SP214 | PHO | Ontario | Adult | 23A | GPSC7 | 65.28 | 2,093,010 | 34 | 197,104 | SRR5011672 |
| SP221 | PHO | Ontario | Adult | 33F | GPSC3 | 46.52 | 2,055,533 | 24 | 159,986 | SRR5011813 |
| SP234 | PHO | Ontario | Adult | 33F | GPSC3 | 48.72 | 1,981,870 | 29 | 110,158 | SRR5011820 |
| SP213 | PHO | Ontario | Adult | 35B | GPSC75 | 106.35 | 2,115,958 | 61 | 65,542 | SRR5011835 |
| SP198 | PHO | Ontario | Adult | 35F | GPSC36 | 64.07 | 2,062,156 | 52 | 76,911 | SRR5011787 |
| SP238 | PHO | Ontario | Adult | 6C | GPSC29 | 92.62 | 2,094,225 | 54 | 78,805 | SRR5011675 |
| SP217 | PHO | Ontario | Adult | 9N | GPSC16 | 105.31 | 2,074,424 | 34 | 143,273 | SRR5011751 |
| SP235 | PHO | Ontario | Adult | 9N | GPSC16 | 153.78 | 2,086,926 | 32 | 124,943 | SRR5011668 |
| SP208 | PHO | Ontario | Adult | 9N | GPSC16 | 111.17 | 2,056,039 | 47 | 97,422 | SRR5011788 |
| SP236 | PHO | Ontario | Adult | 7F | GPSC15 | 181.65 | 2,072,286 | 107 | 47,652 | SRR5011770 |
| SP206 | PHO | Ontario | Adult | 7F | GPSC15 | 108.22 | 2,077,324 | 65 | 65,128 | SRR5011819 |
| SP232 | PHO | Ontario | Adult | 7F | GPSC15 | 181.32 | 2,000,354 | 67 | 69,552 | SRR5011736 |
| SP209 | PHO | Ontario | Adult | 7F | GPSC15 | 75.95 | 2,004,157 | 80 | 59,702 | SRR5011691 |
| SP240 | PHO | Ontario | Adult | 7F | GPSC15 | 187.34 | 2,003,382 | 62 | 58,849 | SRR5011684 |
| SP204 | PHO | Ontario | Adult | 3 | GPSC12 | 132.48 | 2,045,612 | 41 | 90,094 | SRR5011824 |
| SP229 | PHO | Ontario | Adult | 3 | GPSC12 | 173.24 | 2,028,243 | 28 | 149,760 | SRR5011682 |
| SP225 | PHO | Ontario | Adult | 3 | GPSC12 | 329.05 | 2,024,557 | 46 | 126,053 | SRR5011762 |
| SP237 | PHO | Ontario | Adult | 3 | GPSC83 | 286.9 | 1,996,971 | 37 | 148,854 | SRR5011699 |
| SP197 | PHO | Ontario | Adult | 19A | GPSC9 | 63.58 | 2,116,611 | 40 | 70,701 | SRR5011766 |
| SP228 | PHO | Ontario | Adult | 19A | GPSC27 | 183 | 2,129,518 | 109 | 64,025 | SRR5011660 |
| SP231 | PHO | Ontario | Adult | 19A | GPSC27 | 203.3 | 2,120,495 | 64 | 63,461 | SRR5011674 |
| SP212 | PHO | Ontario | Adult | 19A | GPSC1 | 97.34 | 2,060,272 | 56 | 72,060 | SRR5011765 |
| SP233 | PHO | Ontario | Adult | 4 | GPSC27 | 159.58 | 2,131,960 | 44 | 90,096 | SRR5011791 |
| SP224 | PHO | Ontario | Adult | 6B | GPSC24 | 101.13 | 2,170,813 | 63 | 82,574 | SRR5011722 |

^a^ GPSC: Global Pneumococcal Sequence Cluster, as defined by the Global Pneumococcal Sequencing Project.

^b^ DOC: Depth of coverage, calculated as the average number of times each base in the genome was sequenced, assuming an average pneumococcal genome size of approximately 2.1 Mbp

^c^ Number of contigs: Total number of contiguous sequences in the genome assembly.

^d^ N50: The length of the shortest contig at 50% of the total genome assembly length, used as a measure of assembly quality.

^e^ SRA accession number: Sequence Read Archive (SRA) identifier corresponding to the raw sequencing data for each isolate.

^f^ NT. Untypable isolate.

^g^ GPSCNA. GPSC assignment not available.
