# Supplementary material for: Evolving genomic landscape of pediatric pneumococcus in two Canadian urban centers following conjugate vaccination
Source: Front Microbiol. 2025 Aug 18;16:1642658. doi: 10.3389/fmicb.2025.1642658 (PMC12400966; doi:10.3389/fmicb.2025.1642658)
Supplement: Supplementary file 3 [file Table_3.DOCX]

**Supplementary Table 3. Serotype composition of GPSCs with more than one pediatric IPD isolate from Calgary.**

| GPSC ^a^ | Serotype | Number of Genomes |
| --- | --- | --- |
| GPSC1 | 19A | 5 |
|  | 19F | 2 |
| GPSC3 | 11A | 4 |
|  | 18B | 1 |
|  | 18C | 19 |
|  | 33A | 1 |
|  | 33F | 10 |
|  | 8 | 1 |
| GPSC4 | 15C | 3 |
|  | 19A | 11 |
|  | 19F | 5 |
| GPSC5 | 19A | 1 |
|  | 23B | 5 |
| GPSC6 | 14 | 1 |
|  | 15A | 1 |
|  | 15B | 2 |
|  | 15C | 1 |
|  | 24A | 1 |
|  | 9V | 9 |
| GPSC7 | 23A | 2 |
|  | 23B | 4 |
|  | 23F | 9 |
|  | 3 | 1 |
| GPSC8 | 5 | 4 |
| GPSC9 | 14 | 1 |
|  | 15A | 5 |
|  | 19A | 5 |
| GPSC11 | 15B | 1 |
|  | 19F | 1 |
|  | 21 | 1 |
|  | 23B | 2 |
| GPSC12 | 3 | 20 |
| GPSC14 | 23F | 2 |
|  | 6A | 1 |
| GPSC15 | 7F | 9 |
| GPSC16 | 14 | 1 |
|  | 9N | 1 |
| GPSC18 | 14 | 6 |
| GPSC19 | 22F | 19 |
| GPSC24 | 6A | 3 |
|  | 6B | 15 |
| GPSC27 | 19A | 6 |
|  | 4 | 8 |
| GPSC29 | 6A | 1 |
|  | 6C | 1 |
| GPSC32 | 12F | 2 |
| GPSC36 | 10A | 2 |
|  | 35F | 1 |
| GPSC38 | 38 | 6 |
| GPSC39 | 14 | 44 |
| GPSC45 | 34 | 3 |
| GPSC47 | 6B | 3 |
| GPSC48 | 15C | 2 |
| GPSC49 | 17F | 2 |
| GPSC50 | 1 | 1 |
|  | 18B | 1 |
|  | 18C | 5 |
| GPSC64 | 6A | 5 |
| GPSC75 | 29 | 1 |
|  | 35B | 3 |
|  | 35F | 1 |
| GPSC98 | 19F | 1 |
|  | 8 | 3 |
| GPSC99 | 19A | 2 |
|  | 21 | 1 |
| GPSC119 | 19F | 13 |
| TOTAL |  | 314 |
