# Supplementary material for: Evolving genomic landscape of pediatric pneumococcus in two Canadian urban centers following conjugate vaccination
Source: Front Microbiol. 2025 Aug 18;16:1642658. doi: 10.3389/fmicb.2025.1642658 (PMC12400966; doi:10.3389/fmicb.2025.1642658)
Supplement: Supplementary file 4 [file Table_4.DOCX]

**Supplementary Table 4. Temporal changes in VT isolate proportions per GPSC among pediatric IPD cases in Calgary.**

| **GPSC ^a^** | **Nº of isolates** | **P1 ^b^ –**  **% VT ^c^ (Nº of isolates)** | **P2^d^- %**  **VT (Nº of isolates)** | **P3 ^e^ - %**  **VT (Nº of isolates)** |
| --- | --- | --- | --- | --- |
| GPSC1 | 7 | - | 100% (4) | 100% (3) |
| GPSC3 | 36 | 92% (11) | 62% (13) | 0% (11) |
| GPSC4 | 19 | 86% (7) | 89% (9) | 66% (3) |
| GPSC5 | 6 | - | 0% (2) | 25% (4) |
| GPSC6 | 15 | 100% (7) | 100% (3) | 0% (5) |
| GPSC7 | 16 | 82% (11) | 33% (3) | 0% (2) |
| GPSC8 | 4 | - | 100% (4) | - |
| GPSC9 | 11 | 100% (1) | 100% (4) | 17% (6) |
| GPSC10 | 1 | - | 100% (1) | - |
| GPSC11 | 5 | 100% (1) | - | 0% (4) |
| GPSC12 | 20 | 100% (2) | 100% (9) | 100% (9) |
| GPSC13 | 1 | - | 100% (1) | - |
| GPSC14 | 3 | 100% (2) | - | 100% (1) |
| GPSC15 | 9 | 100% (1) | 100% (2) | 100% (6) |
| GPSC16 | 2 | 50% (2) | - | - |
| GPSC18 | 6 | 100% (6) | - | - |
| GPSC19 | 19 | 0% (3) | 0% (5) | 0% (11) |
| GPSC23 | 1 | 100% (1) | - | - |
| GPSC24 | 18 | 100% (9) | 100% (9) | - |
| GPSC27 | 14 | 100% (6) | 100% (3) | 100% (5) |
| GPSC29 | 2 | - | 100% (1) | 0% (1) |
| GPSC31 | 1 | 100% (1) | - | - |
| GPSC32 | 2 | - | - | 0% (2) |
| GPSC36 | 3 | - | 0% (1) | 0% (2) |
| GPSC38 | 6 | 0% (2) | 0% (2) | 0% (2) |
| GPSC39 | 44 | 100% (34) | 100% (10) | - |
| GPSC43 | 1 | 100% (1) | - | - |
| GPSC45 | 3 | 0% (1) | - | 0% (2) |
| GPSC47 | 3 | 100% (3) | - | - |
| GPSC48 | 2 | - | - | 0% (2) |
| GPSC49 | 2 | 0% (1) | 0% (1) | - |
| GPSC50 | 7 | 80% (5) | 100% (2) | - |
| GPSC51 | 1 | 100% (1) | - | - |
| GPSC57 | 1 | - | - | 0% (1) |
| GPSC59 | 1 | - | 0% (1) | - |
| GPSC64 | 5 | 100% (5) | - | - |
| GPSC75 | 5 | - | - | 0% (5) |
| GPSC76 | 1 | - | 100% (1) | - |
| GPSC81 | 1 | - | - | 0% (1) |
| GPSC89 | 1 | - | - | 0% (1) |
| GPSC94 | 1 | - | - | 100% (1) |
| GPSC98 | 4 | - | 0% (1) | 33% (3) |
| GPSC99 | 3 | - | 100% (1) | 50% (2) |
| GPSC107 | 1 | 100% (1) | - | - |
| GPSC109 | 1 | - | 100% (1) | - |
| GPSC119 | 13 | 100% (8) | 100% (4) | 100% (1) |
| GPSC124 | 1 | - | - | 0% (1) |
| GPSC135 | 1 | - | 0% (1) | - |
| GPSC140 | 1 | - | 0% (1) | - |
| GPSC162 | 1 | 100% (1) | - | - |
| GPSC196 | 1 | - | - | 100% (1) |
| GPSC323 | 1 | - | 0% (1) | - |
| GPSC324 | 1 | - | 0% (1) | - |
| GPSC698 | 1 | - | - | 0% (1) |
| NA ^f^ | 2 | 100% (1) | 0% (1) | - |
| Total | 338 |  |  |  |

^a^ GPSC. Global Pneumococcal Sequence Cluster, as defined by the Global Pneumococcal Sequencing Project.

^b^ P1, or the pre-vaccine era is defined as the time before January 1st of the year after the universal introduction of the first PCV vaccine. In Alberta this is before January 1, 2003.

^c^ VT. Vaccine-type serotype, defined as a serotype included in PCV7 or PCV13.

^d^ P2, or the PCV7-era, spans from January 1 of the year after the universal introduction of PCV7, until January 1 of the year after the introduction of PCV13. In Alberta, this is January 1, 2003 - December 31, 2010.

^e^ P3, or the PCV13-era is defined from January 1 of the year after the universal introduction of PCV13, until the end of the collection period. In Alberta, this is January 1, 2011 - December 31, 2016.

^f^ Isolates for which GPSC assignment was not available.
