# Supplementary material for: Evolving genomic landscape of pediatric pneumococcus in two Canadian urban centers following conjugate vaccination
Source: Front Microbiol. 2025 Aug 18;16:1642658. doi: 10.3389/fmicb.2025.1642658 (PMC12400966; doi:10.3389/fmicb.2025.1642658)
Supplement: Supplementary file 5 [file Table_5.DOCX]

**Supplementary Table 5. Serotype distribution of pediatric IPD isolates in Calgary and Toronto during P3 ^a^.**

| **Serotype ^b^** | **Nº of isolates, Calgary (%) ^c^** | | **Nº of isolates, Toronto (%) ^c^** | | ***p*-value ^d^** |
| --- | --- | --- | --- | --- | --- |
| 3 | 9 | (9.1) | 25 | (8.3) | 0.815 |
| 5 | 0 | (0) | 1 | (0.3) | - |
| 6B | 0 | (0) | 1 | (0.3) | - |
| 6C | 2 | (2) | 4 | (1.3) | - |
| 7C | 1 | (1) | 1 | (0.3) | - |
| 7F | 6 | (6.1) | 14 | (4.7) | 0.598 |
| 8 | 2 | (2) | 5 | (1.7) | - |
| 9N | 1 | (1) | 6 | (2) | - |
| 9V | 0 | (0) | 2 | (0.7) | - |
| 10A | 1 | (1) | 7 | (2.3) | - |
| 11A | 3 | (3) | 6 | (2) | - |
| 12F | 2 | (2) | 1 | (0.3) | - |
| 15A | 6 | (6.1) | 6 | (2) | 0.081 |
| 15B | 3 | (3) | 24 | (8) | - |
| 15C | 4 | (4) | 25 | (8.3) | - |
| 16F | 0 | (0) | 2 | (0.7) | - |
| 17F | 0 | (0) | 1 | (0.3) | - |
| 19A | 11 | (11.1) | 76 | (25.3) | 0.003 |
| 19F | 4 | (4) | 1 | (0.3) | - |
| 21 | 2 | (2) | 1 | (0.3) | - |
| 22F | 11 | (11.1) | 28 | (9.3) | 0.606 |
| 23A | 0 | (0) | 14 | (4.7) | - |
| 23B | 7 | (7.1) | 11 | (3.7) | 0.167 |
| 23F | 3 | (3) | 0 | (0) | - |
| 24 | 0 | (0) | 1 | (0.3) | - |
| 24A | 1 | (1) | 0 | (0) | - |
| 24B | 0 | (0) | 1 | (0.3) | - |
| 28A | 0 | (0) | 1 | (0.3) | - |
| 29 | 1 | (1) | 0 | (0) | - |
| 31 | 1 | (1) | 4 | (1.3) | - |
| 33A | 0 | (0) | 4 | (1.3) | - |
| 33F | 8 | (8.1) | 0 | (0) | - |
| 34 | 2 | (2) | 2 | (0.7) | - |
| 35A | 0 | (0) | 1 | (0.3) | - |
| 35B | 3 | (3) | 8 | (2.7) | - |
| 35F | 2 | (2) | 3 | (1) | - |
| 38 | 2 | (2) | 11 | (3.7) | - |
| NT ^e^ | 1 | (1) | 2 | (0.7) | - |
| Total | 99 (100) | | 300 (100) | |  |

^a^ P3, or the PCV13-era, spans from January 1 of the year after the universal introduction of PCV13, until the end of the collection period. In Alberta and Ontario, this is January 1, 2011 - December 31, 2016.

^b^ Vaccine serotypes circulating in P3 are highlighted in light blue.

^c^ Percentages have been rounded and may not total 100%.

^d^ Chi-square test for statistically significant difference between Calgary and Toronto. Tests were performed only where all cells >5.

^e^ NT. Non-typable isolate.
