# Supplementary material for: Evolving genomic landscape of pediatric pneumococcus in two Canadian urban centers following conjugate vaccination
Source: Front Microbiol. 2025 Aug 18;16:1642658. doi: 10.3389/fmicb.2025.1642658 (PMC12400966; doi:10.3389/fmicb.2025.1642658)
Supplement: Supplementary file 6 [file Table_6.DOCX]

**Detailed Supplementary Methods.**

**Bacterial culture and DNA extraction.** Standard laboratory methods were used to identify *S. pneumoniae* isolates by CASPER and TIBDN, where bacterial isolates were maintained. Isolates were revived from frozen stocks and grown overnight at 37°C with 5% CO_2_ on Columbia agar with 5% sheep blood. Bacterial isolates were cultured in 5mL liquid THY broth (Sigma-Alrich, MO, USA) for 4 hours, then centrifuged at 3500rpm for 10 minutes and re-suspended in 300ul TE buffer with 250 units of mutanolysin (Sigma-Aldrich, MO, USA) and 2.5 mg lysozyme (Sigma-Aldrich, MO, USA), and incubated at 37°C for 1 hour. Next, 125uL of 10% SDS (Thermo Fisher Scientific, CA, USA) and 0.25 mg proteinase K (Sigma-Aldrich, MO, USA) were added and incubated at 56°C for 1 hour. DNA was extracted from 200uL of this pretreated sample using Qiagen QiaAMP Blood kit (Qiagen, Germany), according to manufacturer’s directions. Quantification of extracted DNA was performed with the dsDNA Broad Range Assay on a Qubit 2.0 Fluorometer (Thermo Scientific, CA, USA).

**Whole-genome sequencing.** Genomes were prepared using Nextera XT Library Prep Kit (Illumina, CA, USA) at half volume. Fragment length of sequencing libraries was estimated using the Agilent High Sensitivity DNA kit (Agilent, CA, USA). Genomes were sequenced as multiplexed libraries on the Illumina HiSeq4000 platform as paired-end 125-bp reads by the Centre d’expertise et du services at Génome Québec (Montreal, QC, Canada). Strains were de-multiplexed using onboard software.

**Sequence read processing.** Illumina short-reads were stripped of adaptor sequence with BBDuk (<https://jgi.doe.gov/data-and-tools/software-tools/bbtools/bb-tools-user-guide/bbduk-guide/>) and de novo assembled with the A5-miseq pipeline (Coil et al., 2015). The species identification was confirmed with Kraken [v.2] (Wood et al., 2019). Sequence typing was performed with MOST (Tewolde et al., 2016). Sequences were discarded for failure to scaffold (a maximum of 200 contigs was used as the upper threshold), or for having high minor allele frequency (a maximum MAF cutoff of 0.2 was used). Of the CASPER specimens, 14 sequences were discarded: 4 adult sequences due to high MAF; 5 pediatric sequences due to high MAF; 4 pediatric sequences due to excess contigs; 1 pediatric sequence failure. This resulted in a total of 599 CASPER sequences used in this study (338 pediatric, 261 adult). Of the TIBDN pediatric specimens, 9 sequences were discarded: 5 sequences due to high MAF; 2 which had excess contigs; 2 specimens which were unavailable for sequencing. This resulted in a total of 480 pediatric TIBDN sequences used in this study.

The mean number of reads per strain was 2,608,023 (minimum 405,640; maximum 15,627,868 reads). This is an approximate average coverage of 116X (minimum 19X; maximum 760X). The mean de novo assembled genome size was 2,075,205 (minimum 1,968,164; maximum 2,249,850). The mean number of scaffolds was 85 (minimum 21; maximum 198).

**PopPUNK phylogeny and cluster assignment.** A phylogenetic tree was created for all 1,287 genomes using PopPUNK, from de novo sequences. The PopPUNK database was created with a k-mer size of 13, and the model was fit with DBSCAN and refined. All 1,287 sequences were assigned to a Global Pneumococcal Sequencing Project sequencing cluster, according to the v9 database at <https://www.pneumogen.net/gps/#/training#gpsc-assignment>. The databases were downloaded and run locally. Two strains were assigned to novel clusters (see Table S2). The newick tree generated by PopPUNK was subset to the 338 pediatric isolates from Calgary in R (v. 3.6.0) using the ggtree package. This phylogenetic tree is based on pairwise core and accessory genome distances and is used to visualize genetic relationships and cluster structure; it does not represent a phylogeny inferred from sequence alignment and does not include statistical support values for branches.

**Roary core genome phylogeny.** De novo assemblies for all 1,287 sequences were annotated with Prokka v.1.12 (Seemann, 2014), and the gff files were used to calculate the pan-genome using Roary (Page et al., 2015). The total size of the pangenome was 13,552 genes, and the core genome (99%-100%) included 1,060 genes. This core genome was trimmed to remove gaps with TrimAl (v.1.2rev59), to a final trimmed core genome size of 694,401 bp. To calculate a pairwise SNP matrix of each of these 1,287 genomes, we used snp-dist (v.0.6). The pairwise matrix was subset to the 338 pediatric isolates from Calgary, and SNP distance calculations were performed in R. FastTree (Price et al., 2010) was run with a generalized time reversible (GTR) model and a Gamma distribution of rate heterogeneity among sites. While FastTree does not perform traditional bootstrap resampling, it computes local support values using the Shimodaira–Hasegawa (SH)-like approximate likelihood ratio test. These values provide a computationally efficient estimate of branch reliability widely used in bacterial phylogenomics. Phylogenetic trees constructed by FastTreeMP were visualized with the ggtree package of R.

**Supplementary Methods References**

Coil, D., Jospin, G., & Darling, A. E. (2015). A5-miseq: an updated pipeline to assemble microbial genomes from Illumina MiSeq data. Bioinformatics (Oxford, England), 31(4), 587–589. <https://doi.org/10.1093/bioinformatics/btu661>

Page, A. J., Cummins, C. A., Hunt, M., Wong, V. K., Reuter, S., Holden, M. T., Fookes, M., Falush, D., Keane, J. A., & Parkhill, J. (2015). Roary: rapid large-scale prokaryote pan genome analysis. Bioinformatics (Oxford, England), 31(22), 3691–3693. <https://doi.org/10.1093/bioinformatics/btv421>

Price, M. N., Dehal, P. S., & Arkin, A. P. (2010). FastTree 2--approximately maximum-likelihood trees for large alignments. PloS one, 5(3), e9490. <https://doi.org/10.1371/journal.pone.0009490>

Seemann T. (2014). Prokka: rapid prokaryotic genome annotation. Bioinformatics (Oxford, England), 30(14), 2068–2069. <https://doi.org/10.1093/bioinformatics/btu153>

Tewolde, R., Dallman, T., Schaefer, U., Sheppard, C. L., Ashton, P., Pichon, B., Ellington, M., Swift, C., Green, J., & Underwood, A. (2016). MOST: a modified MLST typing tool based on short read sequencing. PeerJ, 4, e2308. <https://doi.org/10.7717/peerj.2308>

Wood, D. E., Lu, J., & Langmead, B. (2019). Improved metagenomic analysis with Kraken 2. Genome biology, 20(1), 257. <https://doi.org/10.1186/s13059-019-1891-0>
