# Supplementary figures and images for: Evolving genomic landscape of pediatric pneumococcus in two Canadian urban centers following conjugate vaccination
Source: Front Microbiol. 2025 Aug 18;16:1642658. doi: 10.3389/fmicb.2025.1642658 (PMC12400966; doi:10.3389/fmicb.2025.1642658)

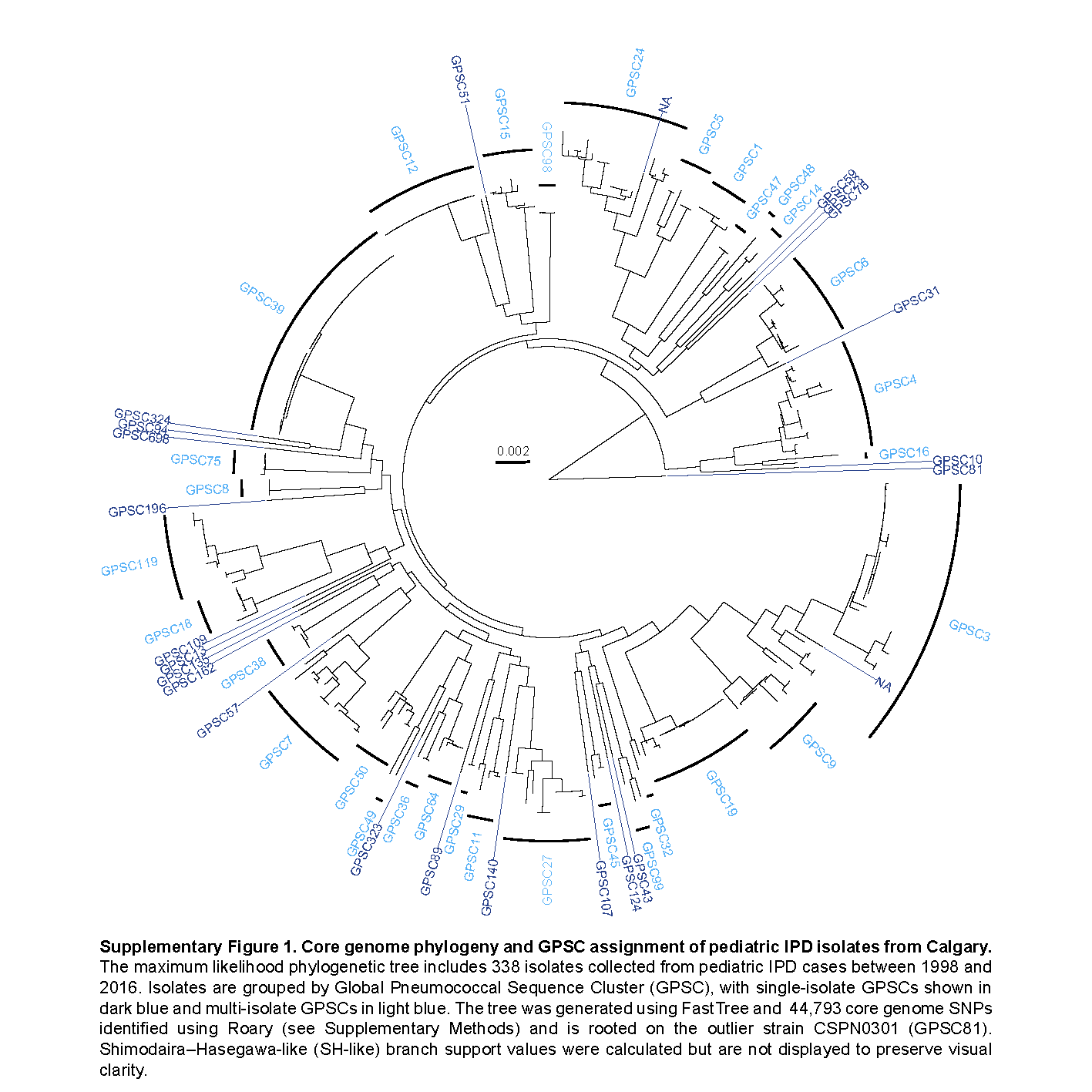

Supplement: Supplementary file 7 [file Image_1.TIFF]

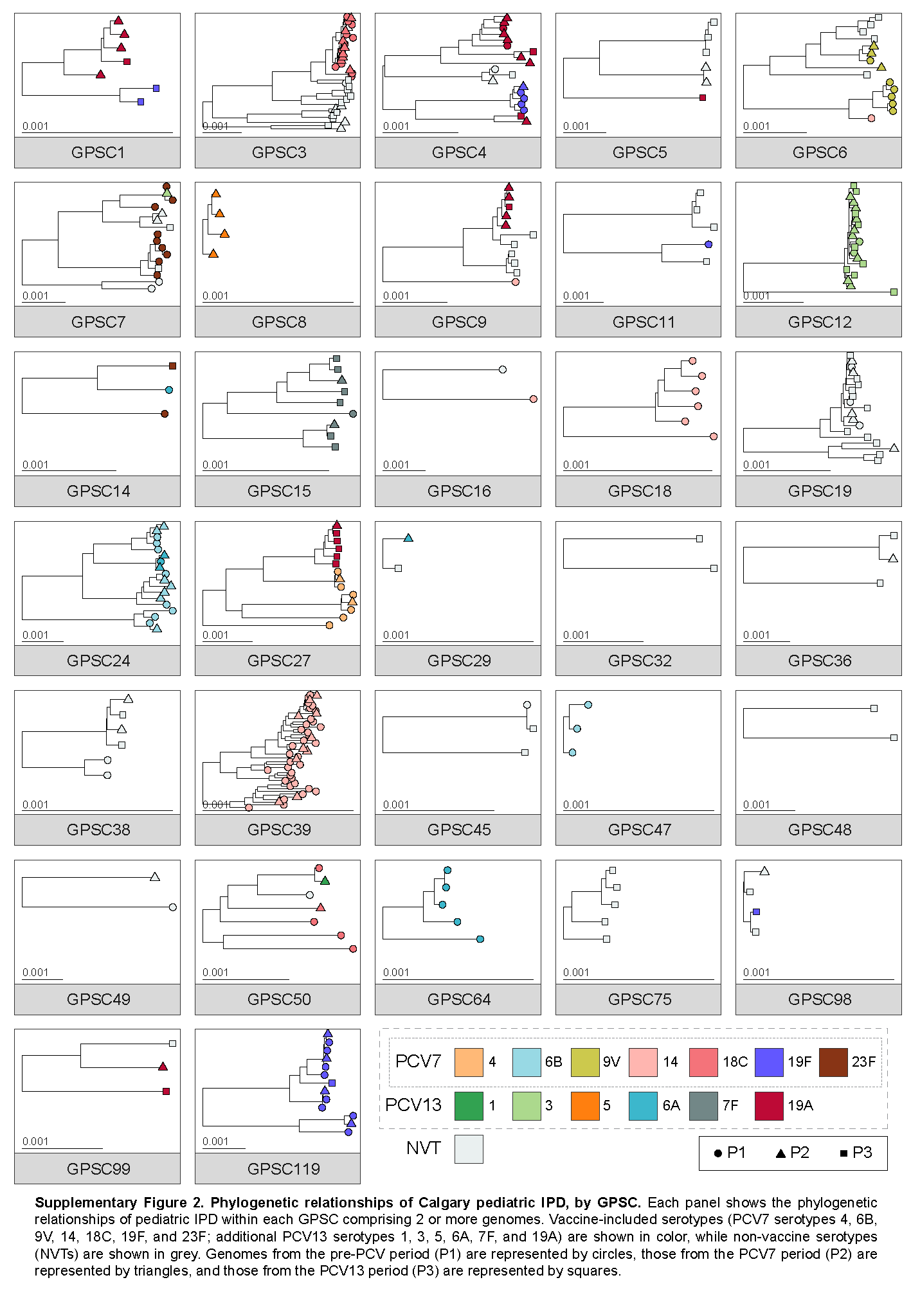

Supplement: Supplementary file 8 [file Image_2.TIFF]

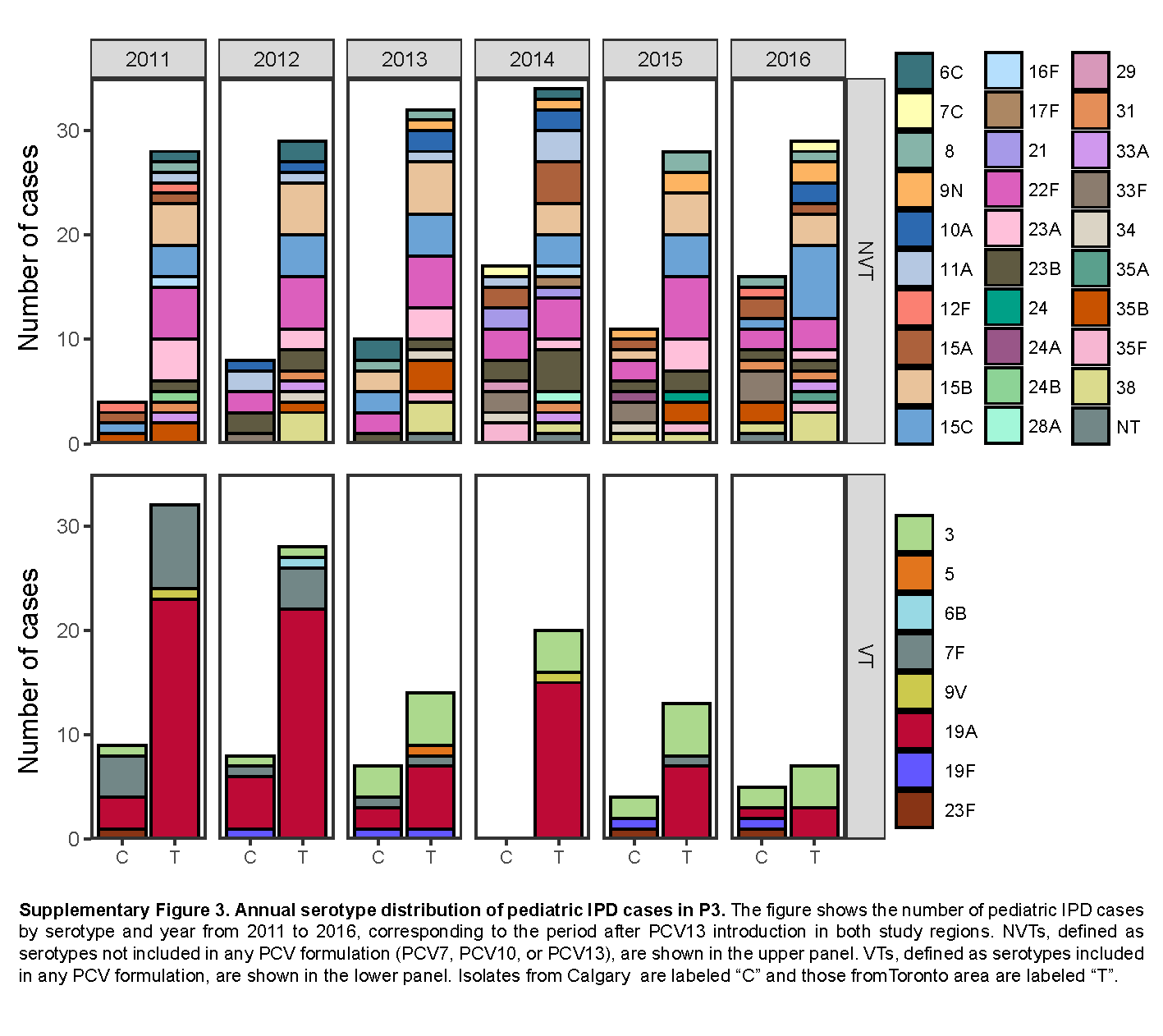

Supplement: Supplementary file 9 [file Image_3.TIFF]

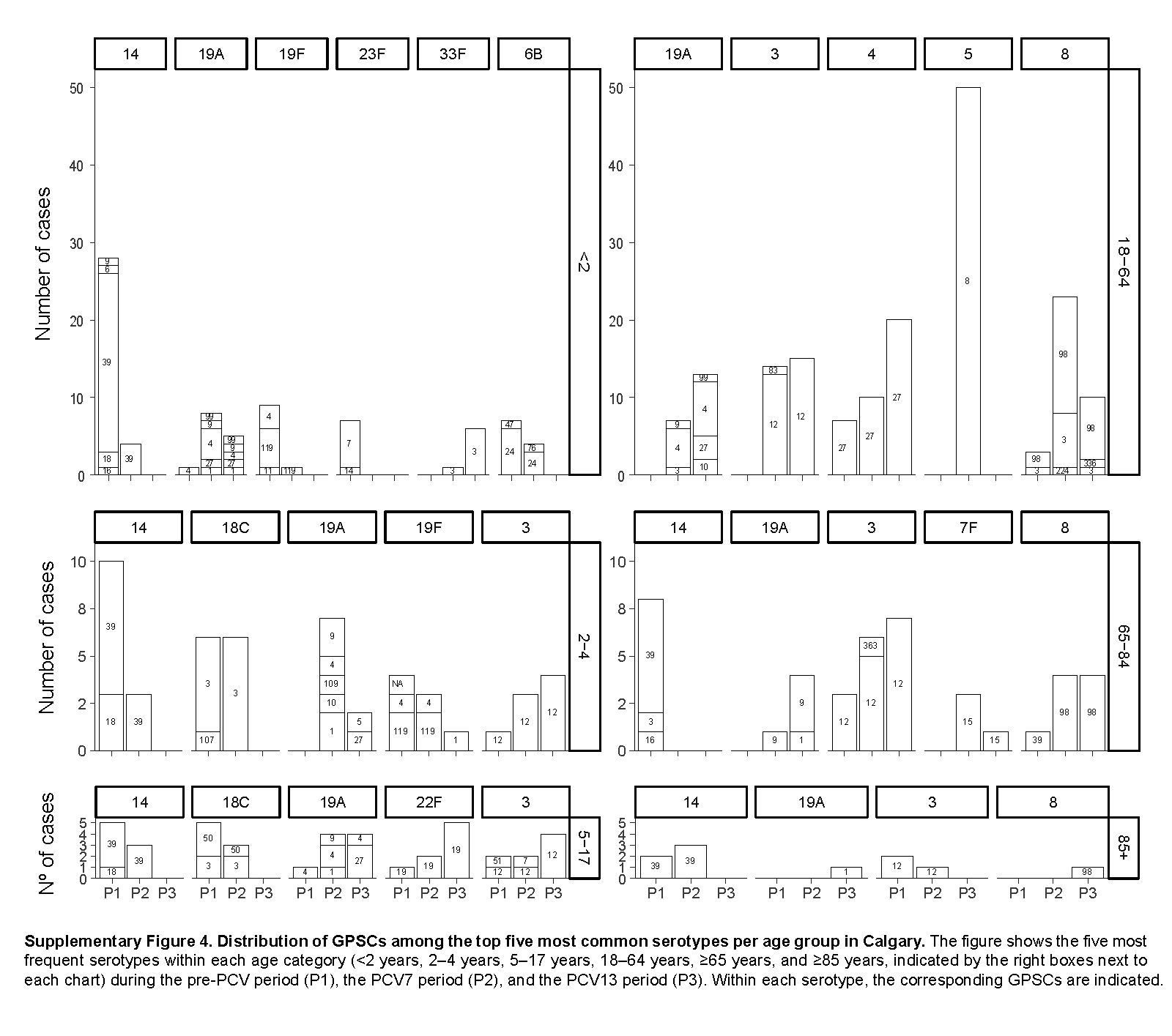

Supplement: Supplementary file 10 [file Image_4.TIFF]

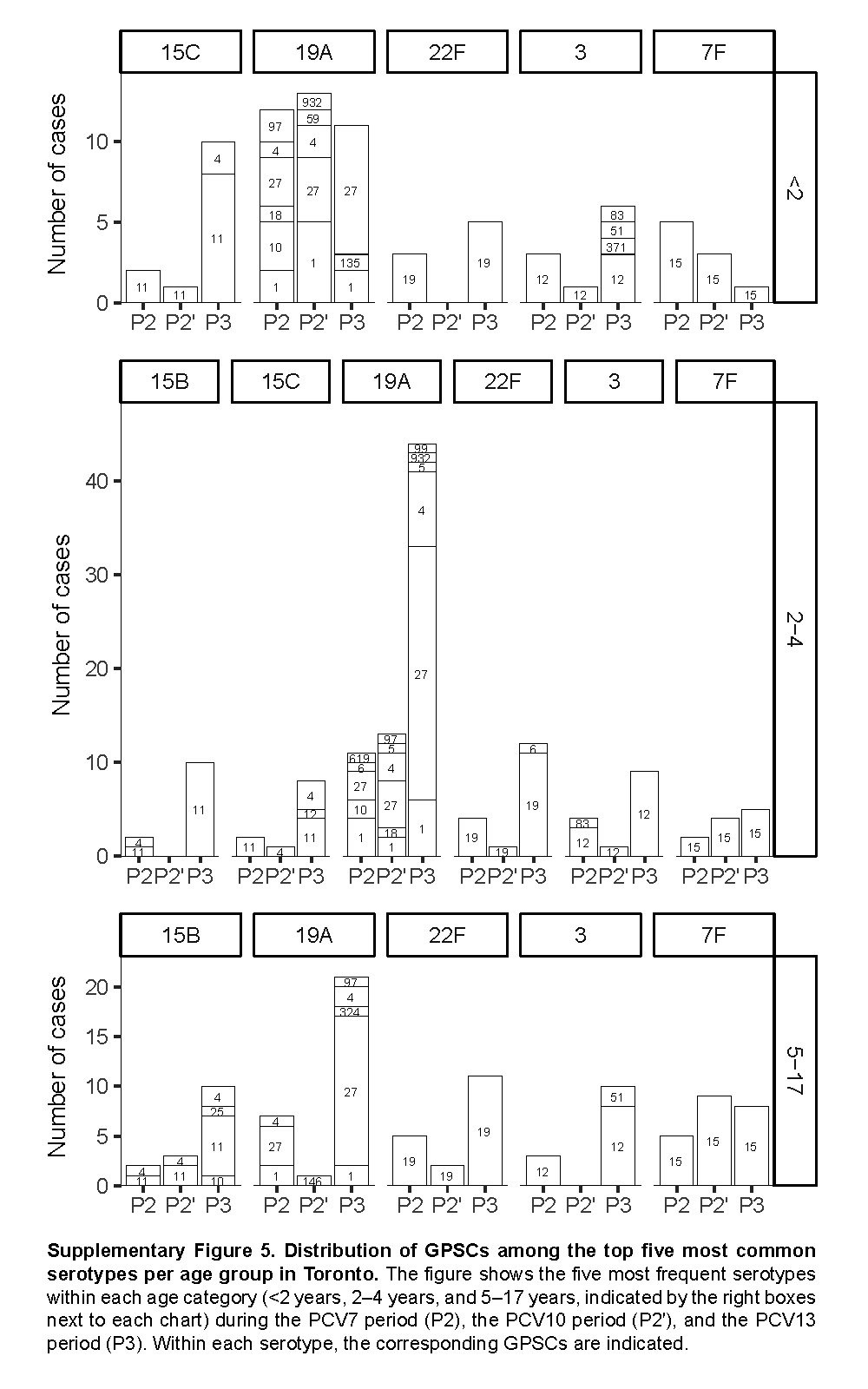

Supplement: Supplementary file 11 [file Image_5.TIFF]
